# Supplementary material for: Structure and function of the geldanamycin amide synthase from Streptomyces hygroscopicus
Source: Nat Commun. 2025 Mar 12;16:2464. doi: 10.1038/s41467-025-57013-3 (PMC11903869; doi:10.1038/s41467-025-57013-3)

Supporting Information

1. Materials and Methods (protein expression, crystallization and assays)
   - 1.1 Expression of *Sh*GdmF
   - 1.2 Simulated structures of *Sh*GdmF and NAT1
   - 1.3 Parameter of refinement
   - 1.4 Binding affinity measurements of co-substrates
   - 1.5 Polder omit maps for the cocyrstallized ligands
   - 1.6 Transformation of thioesters by *Sh*GdmF
   - 1.7 Binding assay for affinity estimation
   - 1.8 Comparison of loop modelling approaches

2. Materials and Methods (Synthesis)
3. Experimental Procedures (Synthesis)
   - 3.1 Truncated thioesters
   - 3.2 3-Hydroxyanilines
   - 3.3 Total synthesis of seco-acid derivatives **26**, **28** and **30**

4. Biotransformation of SNAC ester **27b**
5. References (Supporting Information)

Attachment: Copies of ^{1}H- and ^{13}C-NMR spectra

1. Materials and Methods (protein expression, crystallization and assays)

1.1 Expression of *Sh*GdmF

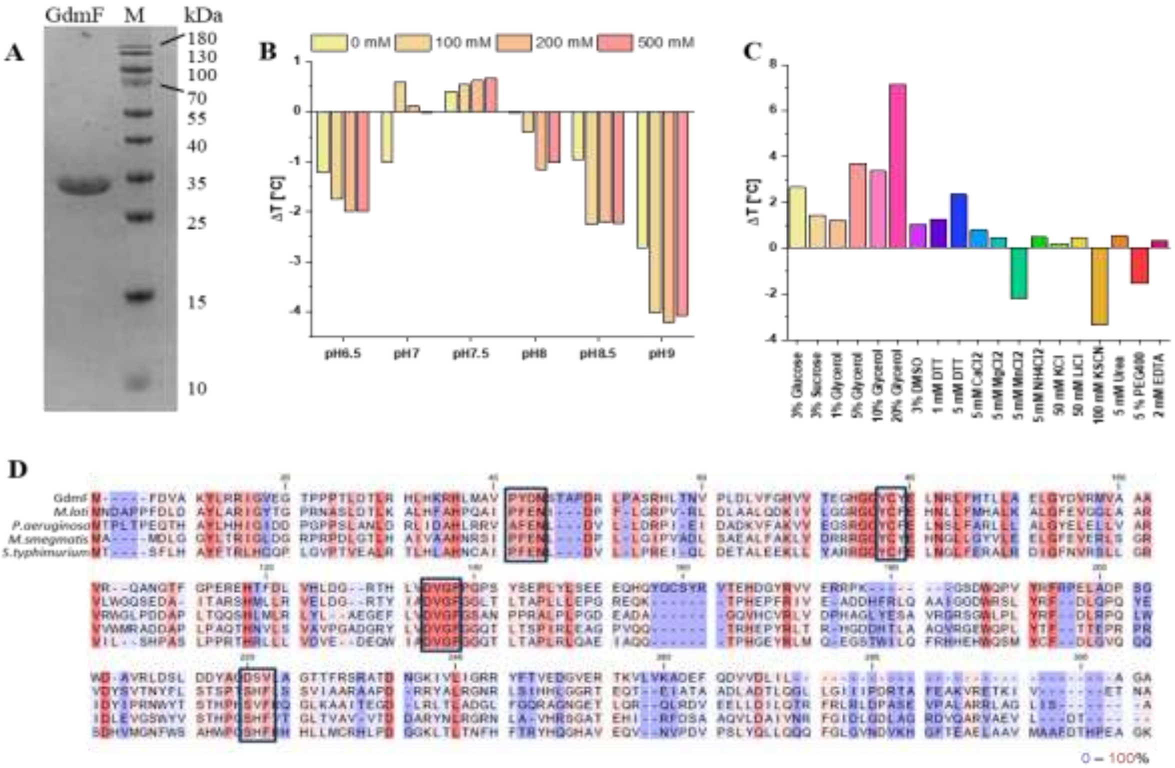

Figure S1: (**A**) SDS-PAGE gel of the purified full-length *Sh*GdmF after gel filtration. The band corresponds to the monomeric *Sh*GdmF with a size of approximately 31 kDa. Marker: PageRuler Plus Pre-stained Protein Ladder. (**B**) Increase or decrease of the protein stability, measured as the difference temperature compared to the determined, inital melting temperature of 38.6 °C (20 mM Tris-HCl buffer, pH 8.0, 150 mM NaCl, and 1 mM DTT) of *Sh*GdmF at different buffer pH and NaCl concentrations. (**C**) Additive test of *Sh*GdmF on the protein stability, measured as the difference temperature compared to the determined, inital melting temperature of 38.6 °C of *Sh*GdmF. (**D**) Sequence alignment of *Sh*GdmF and related NATs. Amino acid conservation is color coded from 0% (blue) to 100% (red).Source data are provided as a Source Data file.

1.2 Simulated structures of *Sh*GdmF and NAT1

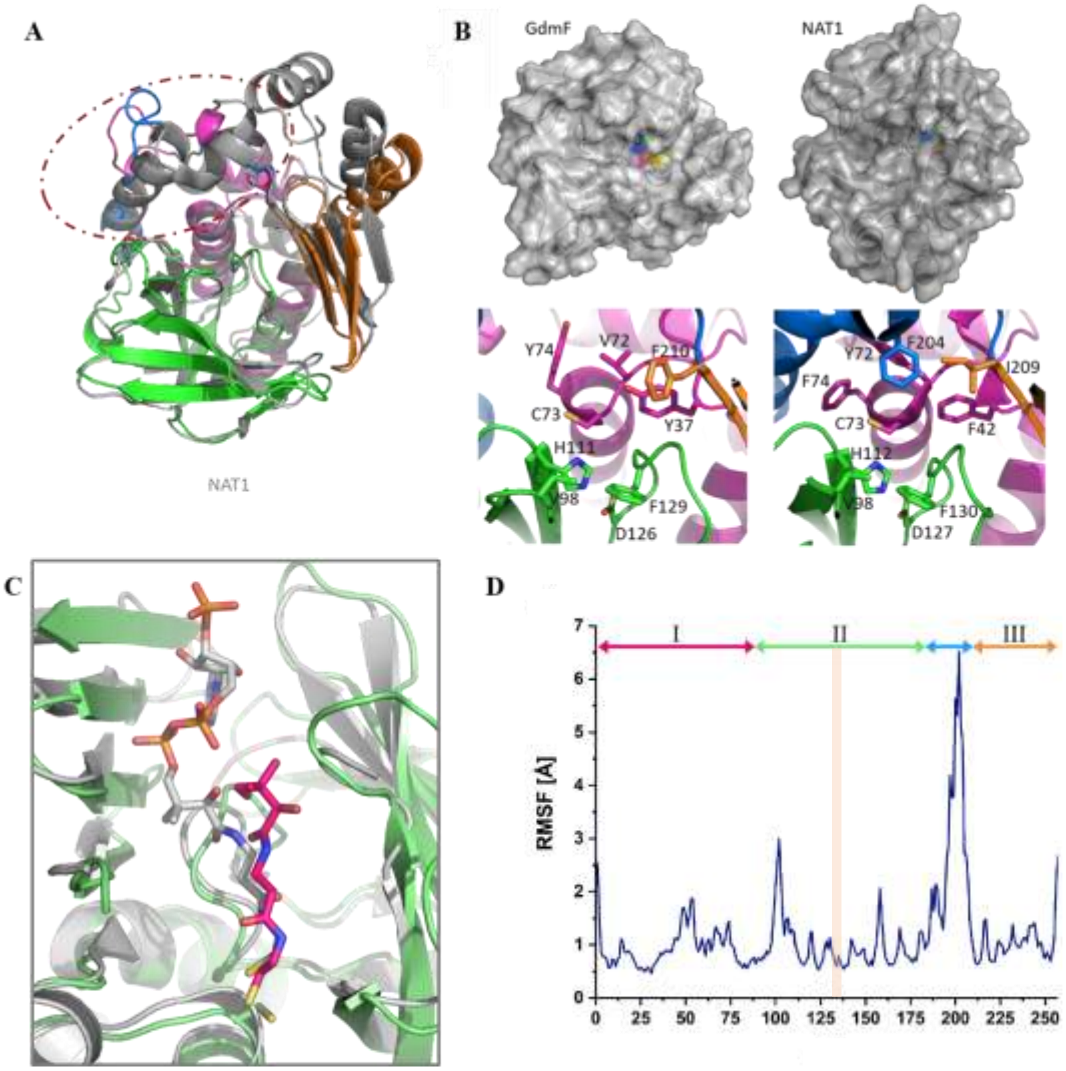

Figure S2: (**A**) Structure comparison of *Sh*GdmF (colored) superposed on *Ml*NAT1 (grey). The largest differences between the two structures can be observed in the interdomain region (blue) and the C-terminal α/β-lid (orange). (**B**) Surface representations (upper panels) and close-up views on the active sites (lower panels) of *Sh*GdmF and *Ml*NAT1. Note the widened active site cleft in GdmF. The catalytic triad is highlighted. (**C**) *Sh*Gdmf (green) in complex pantetheine (purple) superimposed on the structure of *Ml*NAT1 (grey) with bound CoA (pdb: 4nv7). (**D**) Root mean square fluctuations (RMSF) along the 150 ns MD simulations reveal a drastically reduced flexibility of the P-loop (orange) in *Sh*GdmF as compared to *Ml*NAT1, allowing the P-loop to block the CoA binding site. Source data are provided as a Source Data file.

1.3 Parameter of refinement

Table S1: Data collection and refinement statistics.

	**apo *Sh*GdMF**<br>**8btm**	***Sh*GdmF · 13b**<br>**8oom**	***Sh*GdmF · 11b**<br>**8osv**
**Data collection**			
Beamline	Soleil PX-2A	DESY P13	Soleil PX-2A
Space group	C2221	C2221	C2221
Wavelength [Å]	0.98	0.98	0.98
Cell parameters			
a, b, c [Å]	90.93, 144.22, 156.94	72.87, 96.18, 86.75	74.30, 95.29, 86.59
α, β, γ [°]	90, 90, 90	90, 90, 90	90, 90, 90
Rmerge [%]	0.055 (1.139)	0.086 (0.765)	0.060 (1.141)
I / σI	25.56 (2.00)	13.04 (2.11)	19.70 (1.89)
CC1/2	1.00 (0.825)	0.998 (0.72)	0.999 (0.825)
Completeness (%)	99.94 (99.88)	99.29 (99.49)	99.96 (99.92)
**Refinement**			
Resolution [Å]	48.28 – 1.40 (1.45 – 1.40)	48.27 – 1.82 (1.89 – 1.82)	43.30 – 1.28 (1.33 – 1.28)
No. reflections	60043 (5906)	27484 (2723)	79128 (7821)
Rwork / Rfree	17.27 / 18.42	17.02 / 19.60	16.54 / 18.02
No. atoms	2259	2121	2292
Protein	2084	2019	2058
Ligand/ion	16	12	29
Water	159	90	205
B-factors [Å]	23.94	27.42	23.50
Protein	23.14	27.09	22.46
Ligand/ion	46.62	45.63	43.74
Water	32.20	32.29	31.07
R.M.S. deviations			
Bond lengths [Å]	0.010	0.015	0.016
Bond angles [°]	1.19	1.35	1.42

1.4 Binding affinity measurements of co-substrates

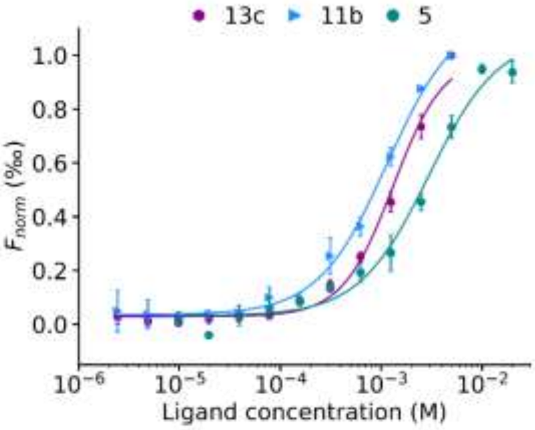

Figure S3: Binding affinity of co-substrates to GdmF as determined using microscale thermophoresis. SNAC co-substrate **11b** showed the highest binding affinity to GdmF with a K_d of 1.16 mM. A comparable affinity was determined for the pantetheine co-substrate **13c** with a K_d of 1.32 mM. Acetyl-CoA (**5**) showed a reduced binding affinity to GdmF with a K_d of 2.96 mM. Data are represented as mean ± SD (n = 3 independent experiments). Source data are provided as a Source Data file.

1.5 Polder omit maps for the cocyrstallized ligands

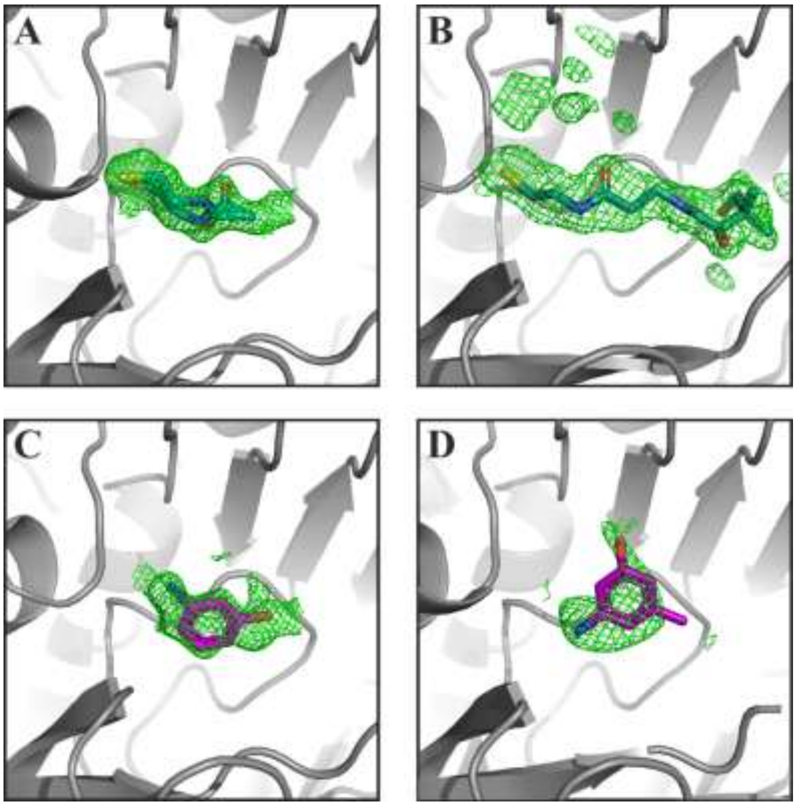

Figure S4: Polder omit maps (green), contoured at 3σ clearly shows electron density for the co-crystallized **(A)** SNAC ligand, **(B)** pantetheine prosthetic arm, **(C)** 3-aminophenol **32**, and **(D)** aminophenol **33**. The electron density for the aminophenols is weak and does not cover the entire ligands.

1.6 Transformation of thioesters by *Sh*GdmF

Table S2: Kinetic parameters for the substrate conversion of thioesters **11a-c** and **13a-c**, and aminophenols **32** and **33** by *Sh*GdmF as obtained from photometric steady-state assays using Ellman's reagent.

Compound	+ aminophenol **32**		
	K_M [µM]	k_{cat} [s^{-1}]	kcat/ K_M [s^{-1} M^{-1}]
**11a**	858.1 ± 232.7	0.0016 ± 1.9·10-4	1.87
**11b**	2735.0 ± 610.3	0.0071 ± 7.0·10-4	2.60
**11c**	2070.2 ± 476.7	0.0087 ± 1.2·10-3	4.20
**13a**	6413.6 ± 2070.4	0.0164 ± 3.9·10-3	2.56
**13b**	1136.8 ± 136.9	0.0027 ± 1.3·10-4	2.37
**13c**	3497.5 ± 1289.9	0.0016 ± 3.1·10-4	0.46
	+ aminophenol **33**		
**11a**	1865.1 ± 253.7	0.0032 ± 2.6·10-4	1.72
**11b**	6902.4 ± 4142.6	0.0120 ± 5.8·10-3	1.74
**11c**	3669.8 ± 1533.3	0.0228 ± 5.8·10-3	6.21
**13a**	3648.3 ± 2812.7	0.0075 ± 5.1·10-3	2.06
**13b**	8267.7 ± 1610.7	0.0084 ± 1.3·10-3	1.02
**13c**	1712.8 ± 475.5	0.0013 ± 1.7·10-4	0.76

1.7 Binding assay for affinity estimation

Although progeldanamycin (**2**) is the cyclization product of *Sh*GdmF, we assumed that the more accessible and structurally similar geldanamycin (**3**) would also provide information on binding with ShGdmF, as is known to be the case with its target protein Hsp90, where ATP binding is the target site. The heat map (Figure S4; supporting information) reveals that Geldanamycin-FITC binds to GdmF and Hsp90 (Figure S4a, A, B, control), and ATP-Cy5 binds to Hsp90 (Figure S4a, C, control; supporting information).

Neither ATP nor geldanamycin bind to Hsp90 in the presence of the known Hsp-inibitor radicicol **35** (Figure S4, B2, C2; supporting information), whereas the binding of geldanamycin-FITC to ShGdmF appears to be slightly increased. The displacement experiments show that thioesters **11a-c, 13b** (Figure S4a) are differentially able to displace geldanamycin-FITC from *Sh*GdmF, indicating that **11a, c** and little or no displacement activity with **11b**, **13b** and radicicol **34**, while **10a** enhances Gda-FITC fluorescence. Thioesters **11a-c** showed little displacement activity on Hsp90. However, dose-response activities were not confirmed for aminophenols **32, 33**, although the activity identified in Figure S3a and acetyl-CoA **34** with ATP-Cy5 on Hsp90 or with Gda-FITC on *Sh*GdmF tended to displace Gda-FITC only at high concentrations >300 µM. Note that the fluorescence intensities were normalized to the control value, while the comparison of intensities between Hsp90a and *Sh*GdmF showed a 50% higher binding activity of Gda-FITC to Hsp90a. The lower susceptibility of *Sh*GdmF could be due to a different open state or accessibility for the speckled protein. However, MST measurements with His tag

labelled *Sh*GdmF gave only for **2** and **11c** stable dose-dependent activities with IC50 values of 819.21 ± 233.16 µM and 423.48 ± 227.14 µM, respectively, (Figure S4b). A comparison of the KM and Kd values for **11c** shows that the Michaelis constant is greater than the dissociation constant indicating that the substrate is rapidly converted into a product. Stable binding data could only be obtained for **11c** and **2** suggesting that the other enzyme-substrate complexes are not stable enough for MST or microarray measurements. Interestingly, the difference of one CH_2 group, which is the difference between **11b** and **11c**, is sufficient for a stable enzyme-substrate complex.

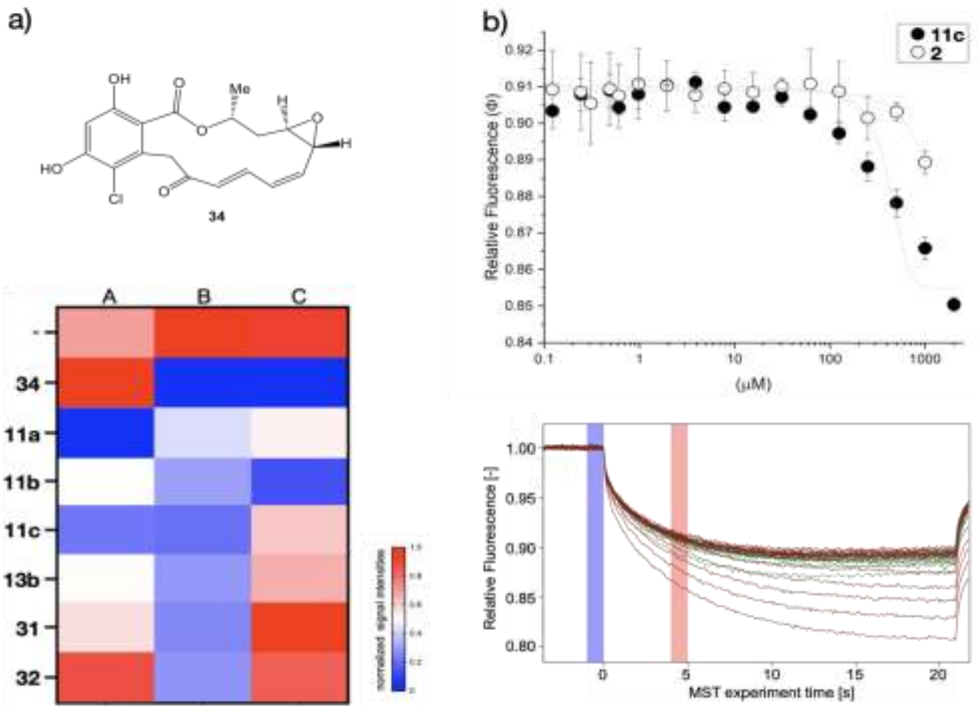

Figure S5: Microarray-based displacement activity on *Sh*GdmF and Hsp90a and MST on *Sh*GdmF. a) Binding of Gda-FITC (1 µM) on spotted *Sh*GdmF (A) and Hsp90a (B) or binding of ATP-Cy5 on Hsp90a (C) analyzed with radicicol **35** (1 µM) or compounds **11a-c**, **13b**, **32**, **33** (10 µM) or without (-). b) Dose-responsive binding activity of **2** and **11c** on Cy5 His-tag labeled *Sh*GdmF by typical MST (lower panel) with corresponding typical MST traces of compound **2** (green) and **11c** (red)**,** respectively. MST-traces are displayed in the mode of Thermophoresis + T-jump. The color of blue and red bars in the figure represents Fcold and Fhot respectively, which show the positions of data collection.

1.8 Electron densities of the GdmF crystal structures

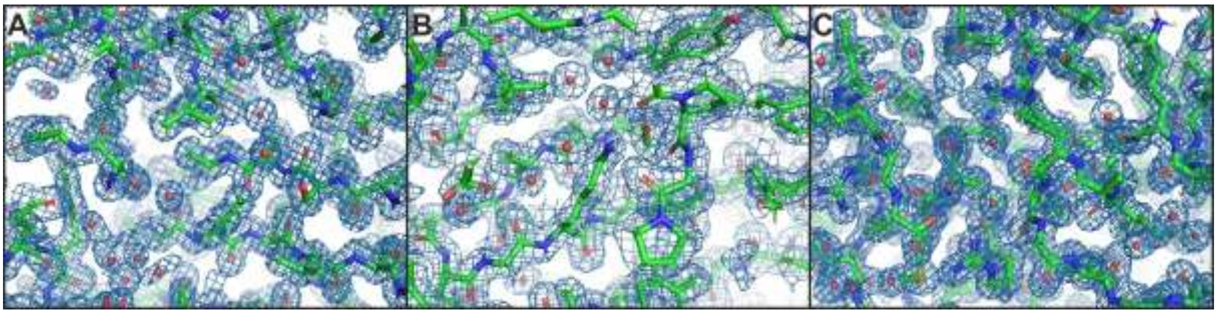

Figure S6: $2F_o$-F_c electron densities maps of the three crystal structures of ligand-free GdmF (**A**, pdb 2btm), GdmF in complex with **13b** (**B**, pdb 8oom), and GdmF in complex with **11b** (**C**, pdb 8osv), countoured at 1σ).

1.9 Comparison of loop modelling approaches

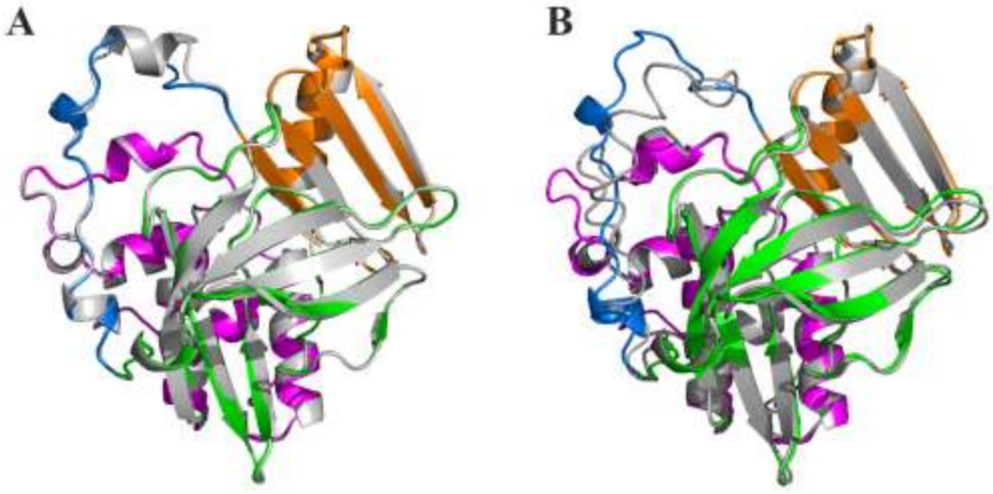

Figure S7: Comparison of different loop modelling strategies. (A) The missing interdomain loop was modelled using comparative modelling and refinement through restraint MD simulations with Modeller (colored protein). The conformation of the modelled interdomain region (blue) correlates well with knowledge-based loop modelling using Yasara (grey). (B) Comparison of the comparative modelled interdomain loop with the best machine learning generated model using Alphafold (grey). The interdomain loop shows a slightly different conformation.

2. Materials and Methods (Synthesis)

Reagents and solvents: All non-aqueous reactions were carried out under an inert atmosphere (argon) with dried glassware, using standard techniques. Anhydrous solvents (such as MeCN, CH_2Cl_2) were obtained from a MB solvent purification system (MBRAUN) or commercial solvents were used. Petroleum ether (60 °C) and THF were distilled before application and triethylamine was dried over KOH and distilled as well. Commercial reagents were used as supplied.

Thin layer chromatography (TLC): Analytical thin-layer chromatography was performed on precoated aluminium-backed silica gel plates with a layer thickness of 0.2 mm. Visualization of the developed chromatogram was performed by UV absorbance (254 nm) and/or stained with aqueous potassium permanganate solution with subsequent heat treatment.

Flash column chromatography: Flash column chromatography was performed using mesh silica (grain size 40-63 μm), with the indicated solvent system according to the standard techniques. Alternatively, a BÜCHI purification system was applied containing two pump modules (C-605), a UV-Vis detector (C-630), a fraction collector (C-660) and the control unit C-620. The separation was performed with a Cartridge PP 12/150 column and a FC60 (60 x 20 mL) rack. The system was controlled via Sepacore® control software.

Nuclear magnetic resonance (NMR) spectroscopy: NMR spectra were recorded on a BRUKER Ultrashield 500 MHz with Avance-III HD console, an Ascend 400 MHz with Avance-III console, an Ascend 400 MHz with Avance-III HD console, an Ultrashield 400 MHz with Avance-I console and an Ascend 600 MHz with Avance Neo console.

Chemical shifts for ^{1}H-NMR spectra are recorded in parts per million from tetramethylsilane with the residual protic solvent resonance as the internal standard ($CDCl_3$: δ 7.26 ppm, CD_3OD: δ 3.31 ppm, $(CD_3)_2SO$: δ 2.50 ppm, C_6D_6: δ 7.16 ppm, D_2O: δ 4.79 ppm, CD_3CN: δ 1.94 ppm). Data are reported as follows: chemical shift (multiplicity [s = singlet, bs = broad singlet, d = doublet, dd = doublet of

doublets, t = triplet, q = quartet, quin = quintet, oct = octet and m = multiplet], coupling constant (in Hz), integration and assignment). All multiplet signals were quoted over a chemical shift range.
^{13}C-NMR spectra are recorded with complete proton decoupling. Chemical shifts are reported in parts per million from tetramethylsilane with the solvent resonance as the internal standard ($CDCl_3$: δ 77.00 ppm, CD_3OD: δ 49.00 ppm, $(CD_3)_2SO$: δ 39.52 ppm, C_6D^6: δ 128.06 ppm, CD_3CN: δ 1.32 ppm, 118.26 ppm). The multiplicities are corresponding to the non-decoupled spectra and are described as follows: p = primary, s = secondary, t = tertiary, q = quaternary.
Assignments of ^{1}H- and ^{13}C-spectra were based upon the analysis of δ- and J-values, as well as COSY, HMBC, HSQC and adequate experiments where appropriate.
Mass spectrometry (MS): High resolution mass spectrometry (HRMS) was measured with a Micromass LCT with lockspray source. The injection proceeded in loop-mode with a HPLC system by WATERS (Alliance 2695). Alternatively, mass spectra were recorded with a Acquity-UPLC system by WATERS in combination with a QTOF Premier mass spectrometer by WATERS in lockspray mode. The ionization happened by electrospray ionization (ESI) or by chemical ionization at atmospheric pressure (APCI). The calculated and found mass are reported.
High performance liquid chromatography (HPLC): Semi-preparative HPLC was performed using an Alliance 2695 HPLC-system by WATERS with a WATERS 996 diode array detector (λ = 200-350 nm) and a Nucleodur C18 HTec column (5 μm, 250 mm, Ø 8 mm) by MACHEREY-NAGEL. Mass detection was conducted with a WATERS Quattro micro API mass spectrometer in negative ionization mode.
Preparative HPLC was performed using a GILSON HPLC-system (pump 331/332) with additional MERCK HITACHI Split-Pump (L-6200A, UV-Vis detector L-4250) and a MACHEREY-NAGEL Nucleodur C18 ISIS column (5 μm, 250 mm, Ø 21 mm with guard cartridge, 40 mm, Ø 21 mm). Mass detection was conducted with a WATERS Micromass ZQ mass spectrometer in negative ionisation mode. Operating conditions and retention times (tR) are reported in the experimental details.
Melting points: Melting points were determined on a SRS OptiMelt apparatus and are not corrected.
Optical Rotation: Specific optical rotation values $[\alpha]t$D were measured in a quartz cuvette on a polarimeter 341 by PERKINELMER at a wavelength of 589 nm (D) and given temperature t. Concentrations c are given in g/100 mL solvent.
Freeze-pump-thaw-technique (fpt): Degassed solvents were prepared by the fpt technique. For this, the appropriate dry solvent was placed under an argon atmosphere in a SCHLENK flask being connected to the SCHLENK line. The solvent was frozen in the flask using liquid nitrogen. Then the stopcock was opened to vacuum and the atmosphere was evaporated for 5 minutes. The flask was sealed and thawed until the solvent melted using an acetone bath being replaced by the cooling bath in order to repeat these steps until a gas evolution at the solution was no longer seen. A minimum of three cycles was needed. Subsequently, the flask was filled with argon gas and sealed. The solvent was ready to use.

3. Experimental Procedures (Synthesis)

3.1 Truncated thioesters

3.1.1 Pantetheine thioesters

Pantetheine dimethylketal (S1)

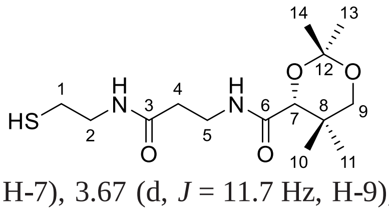

S1

The pantetheine dimethyl ketal **S1**Fehler! Verweisquelle konnte nicht gefunden werden. was prepared according procedure published protocol reported by Townsend *et al.*[S1] in 87 % yield (1.45 g, 4.57 mmol). ^{1}H-NMR (400 MHz, $CDCl_3$, $CHCl_3$ = 7.26 ppm): δ 7.07 (s, 1H, NH), 6.62 (s, 1H, NH), 4.08 (s, 1H, H-7), 3.67 (d, J = 11.7 Hz, H-9), 3.61 – 3.36 (m, 4H, H-2, H-5) 2.27 (d, J = 11.7 Hz, H-9'), 2.66 (q,

J = 6.8 Hz, 2H, H-1), 2.51 (t, J = 6.0 Hz, 2H, H-4), 1.45 (s, 3H, Me-12/13), 1.41 (s, 3H, Me-12/13), 1.03 (s, 3H, Me-10/11), 0.96 (s, 3H, Me-10/11); ^{13}C-NMR (400 MHz, $CDCl_3$ = 77.16 ppm): δ 171.2 (s, C-3), 170.4 (s, C-6), 99.2 (s, C-12), 77.3 (d, C-7), 71.5 (t, C-9), 42.6 (t, C-2), 36.2 (t, C-4), 35.0 (t, C-5), 33.1 (s, C-8), 29.6 (q, C-13/14), 24.6 (t, C-1), 22.2 (q, C-10/11), 19.0 (q, C-10/11), 18.8 (q, C-13/14); m.p. 100 °C, ref. [S1] 99-101 °C; $[\alpha]_D^{24}$ +45.0 (c = 0.9, $CHCl_3$), ref. [S1] $[\alpha]_D^{24}$ +48.0 (c = 1.0, $CHCl_3$); HRMS-ESI *m/z* for $C_{14}H_{27}N_2O_5S$ [M+H]+ calc. 319.1692, found: 319.1692.

General procedure for the synthesis of acyl pantetheine dimethylketals

Pantetheine dimethyl ketal (0.155 g, 0.49 mmol, 1.0 eq.) was dissolved in CH_2Cl_2 (3 mL) and the respective carboxylic acid (0.49 mmol, 1.0 eq.) was added. The mixture was cooled to 0 °C and DMAP (0.048 g, 0.39 mmol, 0.8 eq.) and EDC·HCl (0.187 g, 0.98 mmol, 2.0 eq.) were added subsequently. The mixture was then allowed to reach room temperature and stirred for 2 h. The reaction was terminated by addition of a 2 M HCl solution (8 mL), the layers were separated and the aqueous phase was extracted with CH_2Cl_2 (3 x 3 mL). The combined organic layers were dried over $MgSO_4$, filtered and the solvent was removed under reduced pressure. The crude product was purified by flash column chromatography (100 % ethyl acetate).

Acetyl pantetheine dimethylketal (S2 Fehler! Verweisquelle konnte nicht gefunden werden.**)**

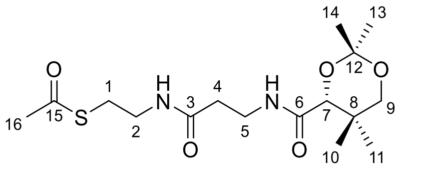

S2

Acetyl pantetheine dimethylketal (**S2**Fehler! Verweisquelle konnte nicht gefunden werden., 0.122 g, 0.38 mmol, 78 %) was obtained as a colorless oil. ^{1}H-NMR (400 MHz, CDCl3, $CHCl_3$ = 7.26 ppm): δ 7.02 (t, J = 6.3 Hz, 1H, NH), 6.60 (bs, 1H, NH), 4.03 (s, 1H, H-7), 3.63 (d, J = 11.7 Hz, 1H, H-9), 3.56 – 3.42 (m, 2H, H-5), 3.42 – 3.30 (m, 2H, H-2) 3.22 (d, J = 11.7 Hz, 1H, H-9'), 2.96 (t, J = 6.6 Hz, 2H, H-1), 2.40 (t, J = 6.2 Hz, 2H, H-4), 2.30 (s, 3H, Me-16), 1.41 (s, 3H, Me-13/14), 1.37 (s, 3H, Me-12/13), 0.98 (s, 3H, Me-10/11), 0.92 (s, 3H, Me-10/11) ppm; ^{13}C-NMR (400 MHz, $CDCl_3$ = 77.16 ppm): δ 196.1 (s, C-15), 171.4 (s, C-3), 170.3 (s, C-6), 99.1 (s, C-12), 77.2 (d, C-7), 71.4 (t, C-9), 39.4 (t, C-2), 35.9 (t, C-4), 34.9 (t, C-5), 33.0 (s, C-8), 30.7 (q, C-16), 29.5 (q, C-13/14), 28.7 (t, C-1), 22.2 (q, C-10/11), 20.0 (q, C-10/11), 18.7 (q, C-13/14); HRMS-ESI *m/z* for $C_{16}H_{28}N_2O_5SNa$ [M+Na]+ calc. 383.1617, found: 383.1616.

(*E*)-Pent-2-enoyl pantetheine dimethylketal (S3)

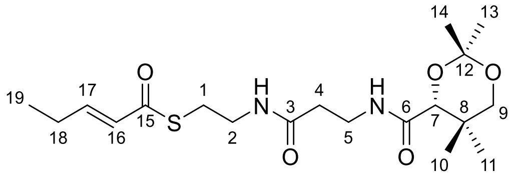

S3

(*E*)-Pent-2-enoyl Pantetheine dimethylketal (**S3**, 0.125 g, 0.31 mmol, 64 %) was obtained as a colorless oil. ^{1}H-NMR (400 MHz, MeOD-d4, MeOH = 3.31 ppm): δ 8.22 (bs, 1H, NH), 7.65 (bs, 1H, NH), 6.98 (dt, J = 15.4, 6.5 Hz, 1H, H-17), 6.16 (dt, J = 15.5, 1.7 Hz, 1H, H-16), 4.12 (s, 1H, H-7), 3.73 (d, J = 11.7 Hz, 1H, H-9), 3.48 – 3.43 (m, 2H, H-2), 3.35 (t, J = 6.6 Hz, 2H, H-5), 3.26 (d, J = 11.6 Hz, 1H, H-9'), 3.06 (t, J = 6.6 Hz, 2H, H-1), 2.40 (t, J = 6.6 Hz, 2H, H-4), 2.28 – 2.21 (m, 2H, H-18), 1.45 (s, 3H, Me-13/14), 1.44 (s, 3H, Me-13/14), 1.07 (t, J = 7.4 Hz, 3H, H-19), 0.99 (s, 3H, Me-10/11), 0.97 (s, 3H, Me-10/11) ppm; ^{13}C-NMR (400 MHz, MeOD = 49.00 ppm): δ 191.0 (s, C-15), 173.8 (s, C-3), 172.1 (s, C-6), 148.8 (d, C-17), 128.6 (d, C-16), 100.4 (s, C-12), 78.4 (d, C-7), 72.3 (t, C-9), 40.2 (t, C-2), 36.3 (t, C-4), 36.1 (t, C-5), 34.0 (s, C-8), 29.7 (q, C-13/14), 28.9 (t, C-1), 26.2 (t, C-18), 22.4 (q, C-10/11), 19.4 (q, C-10/11), 19.0 (q, C-13/14), 12.5 (q, C-19); HRMS-ESI *m/z* for $C_{19}H_{32}N_2O_5SNa$ [M+Na]+ calc. 423.1921, found: 423.1920.

(*E*)-2-Methylbut-2-enoyl pantetheine dimethylketal (S4)

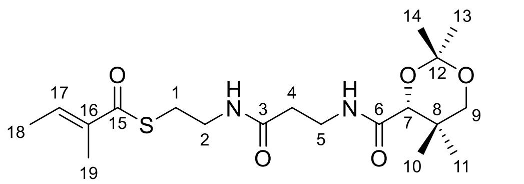

(*E*)-2-Methylbut-2-enoyl Pantetheine Dimethyl Ketal (**S4**, 0.143 g, 0.40 mmol, 81 %) was obtained as a colorless oil. The analytical data are consistent with those reported in the literature.[S2] ^{1}H-NMR (400 MHz, $CDCl_3$, $CHCl_3$ = 7.26 ppm): δ 7.02 (t, *J* = 5.7 Hz, 1H, NH), 6.81 – 6.76 (m, 1H, H-17), 6.64 (bs, 1H, NH), 4.01 (s, 1H, H-7), 3.61 (d, *J* = 11.6 Hz, 1H, H-9), 3.49 – 3.36 (m, 4H, H-2, H-5), 3.20 (d, *J* = 11.6 Hz, 1H, H-9'), 2.98 (t, *J* = 6.5 Hz, 2H, H-1), 2.37 (t, *J* = 6.2 Hz, 2H, H-4), 1.79 (s, 3H, H-19), 1.76 (d, *J* = 7.1 Hz, 3H, H-18), 1.38 (s, 3H, Me-13/14), 1.35 (s, 3H, Me-13/14), 0.95 (s, 3H, Me-10/11), 0.90 (s, 3H, Me-10/11) ppm; ^{13}C-NMR (400 MHz, $CDCl_3$ = 77.16 ppm): δ 193.5 (s, C-15), 171.4 (s, C-3), 170.2 (s, C-6), 136.8 (d, C-17), 136.8 (q, C-16), 99.1 (s, C-12), 77.1 (d, C-7), 71.4 (t, C-9), 39.6 (t, C-2), 35.8 (t, C-4), 34.9 (t, C-5), 32.9 (s, C-8), 29.4 (q, C-13/14), 28.3 (t, C-1), 22.1 (q, C-10/11), 18.9 (q, C-10/11), 18.7 (q, C-13/14), 14.5 (q, C-18), 12.1 (q, C-19); HRMS-ESI *m/z* for $C_{19}H_{32}N_2O_5SNa$ [M+Na]+ calc. 423.1921, found: 423.1919.

General procedure for the $InCl_3$-mediated acetonide deprotection of acyl pantetheine dimethylketals to furnish acyl pantetheines[S3]

Acyl pantetheine dimethylketal (1.0 eq.), $InCl_3$ (2.0 eq.) and H_2O (4.0 eq.) were dissolved in MeCN (2 mL) and the mixture was stirred at r.t. for 4 h. The reaction was terminated by the addition of water and CH_2Cl_2 (10 mL) was added. The layers were separated and the aqueous layer was extracted with CH_2Cl_2 (2x10 mL). The combined organic phases were dried over $MgSO_4$, filtered and concentrated. The crude product was purified by column chromatography (silica gel, CH_2Cl_2/MeOH = 30:1 → 15:1).

Acetyl Pantetheine (13a)

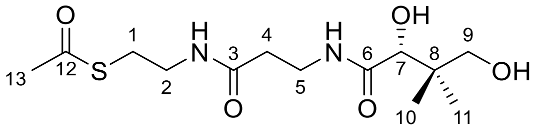

Acetyl pantetheine (**13a**, 25.0 mg, 0.078 mmol, 50 %) was obtained as a colorless oil. The analytical data are consistent with those reported in the literature.[S4] ^{1}H-NMR (400 MHz, MeOD-d4, MeOH = 3.31 ppm): δ 3.89 (s, 1H, H-7), 3.50 – 3.33 (m, 6H, H-2, H-5, H-9), 3.00 (t, *J* = 6.7 Hz, 2H, H-1), 2.41 (t, *J* = 6.7 Hz, 2H, H-4), 2.33 (s, 3H, Me-13), 0.92 (s, 6H, Me-10, Me-11) ppm; ^{13}C-NMR (400 MHz, MeOD-d4 = 49.00 ppm): δ 197.0 (s, C-12), 176.1 (s, C-6), 173.9 (s, C-3), 77.3 (d, C-7), 70.3 (t, C-9), 40.4 (s, C-2), 40.0 (t, C-8), 36.4 (t, C-4), 36.3 (t, C-5), 30.5 (q, C-13), 29.4 (t, C-1), 21.3 (q, C-10/11), 20.9 (q, C-10/11); $[\alpha]_{D}^{20}$ = + 19.2 (*c* = 0.8, MeOH), ref. [S4] $[\alpha]_{D}^{20}$ = + 22.0 (*c* = 1.0, $CHCl_3$); HRMS-ESI *m/z* for $C_{13}H_{24}N_2O_5SNa$ $[M+Na]^+$ calc. 343.1304, found: 343.1303.

(*E*)-Pent-2-enoyl pantetheine (13b)

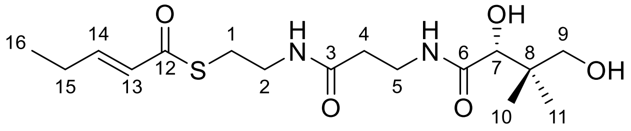

(*E*)-Pent-2-enoyl pantetheine (**13b**, 23.1 mg, 0.064 mmol, 32 %) was obtained as a colorless oil. ^{1}H-NMR (400 MHz, MeOD-d4, MeOH = 3.31 ppm): δ 6.89 (dt, *J* = 15.4, 6.5 Hz, 1H, H-14), 6.16 (dt, *J* = 15.5, 1.7 Hz, 1H, H-13), 3.89 (s, 1H, H-7), 3.53 – 3.33 (m, 6H, H-2, H-5, H-9), 3.06 (t, *J* = 6.7 Hz, 2H, H-1), 2.41 (t, *J* = 6.7 Hz, 2H, H-2), 2.29 – 2.21 (m, 2H, H-15), 1.08 (t, *J* = 7.4 Hz, 3H, H-16), 0.92 (s, 6H, H-10, H-11) ppm; ^{13}C-NMR (400 MHz, MeOD-d4 = 49.00 ppm): δ 191.2 (s, C-12), 176.0 (s, C-6), 173.9 (s, C-3), 148.8 (d, C-14), 128.6 (d, C-13), 77.3 (d, C-7), 70.4 (t, C-9), 40.4 (t, C-8), 40.2 (s, C-2), 36.4 (t, C-4), 36.4 (t, C-5), 28.9 (t, C-1), 26.2 (t, C-15), 21.4 (q, C-10/11), 20.9 (q, C-10/11), 12.5 (q, C-16);

$[\alpha]_D^{20}$ = + 22.7 (c = 2.3, MeOH); HRMS-ESI *m/z* for $C_{16}H_{28}N_2O_5SNa$ [M+Na]+ calc. 383.1617, found: 383.1617.

(*E*)-2-Methylbut-2-enoyl Pantetheine (13c)

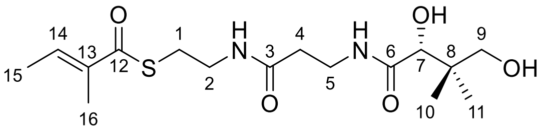

13c

(*E*)-2-Methylbut-2-enoyl pantetheine (**13c**, 37.6 mg, 0.104 mmol, 52 %) was obtained as a colorless oil. The analytical data are consistent with those reported in the literature [S2]. ^{1}H-NMR (400 MHz, MeOD-d4, MeOH = 3.31 ppm): δ 6.88 (q, J = 6.6 Hz, 1H, H-14), 3.89 (s, 1H, H-7), 3.50 – 3.33 (m, 6H, H-2, H-5, H-9), 3.03 (t, J = 6.7 Hz, 2H, H-1), 2.41 (t, J = 6.6 Hz, 2H, H-4), 1.85 (s, 3H, H-16), 1.84 (d, J = 7.5 Hz, 3H, H-15), 0.92 (s, 6H, Me-10, Me-11) ppm; ^{13}C-NMR (400 MHz, MeOD-d4 = 49.00 ppm): δ 194.4 (s, C-12), 176.1 (s, C-6), 173.9 (s, C-3), 138.2 (d, C-14), 137.6 (s, C-13), 77.3 (d, C-7), 70.3 (t, C-9), 40.4 (s, C-8), 40.2 (t, C-2), 36.4 (t, C-4), 36.3 (t, C-5), 29.0 (t, C-1), 21.3 (q, C-10), 21.0 (q, C-11), 14.4 (q, C-15), 12.1 (q, C-16) ppm; $[\alpha]_D^{20}$ = + 12.1 (c = 0.9, MeOH); HRMS-ESI *m/z* for $C_{16}H_{28}N_2O_5SNa$ $[M+Na]^+$ calc. 383.1617, found: 383.1616.

3.1.2 SNAC thioesters

General procedure for the synthesis of SNAC thioesters

N-Acetylcysteamine (238.4 mg, 2.0 mmol, 1.0 eq.) and the respective carboxylic acid (2.0 mmol, 1.0 eq.) were dissolved in CH_2Cl_2 (10 mL) and cooled to 0 °C. Then, DMAP (48.9 mg, 0.40 mmol, 0.2 eq.) and EDC•HCl (383.4 mg, 2.0 mmol, 1.0 eq.) were added subsequently. The mixture was allowed to reach ambient temperature and stirring was continued for 3 h. The reaction was then terminated by addition of a 2 M HCl solution (5 mL) and the layers were separated. The aqueous phase was extracted with CH_2Cl_2 (3 x 10 mL) and the combined organic phases were washed with a saturated aqueous $NaHCO_3$ solution and brine, dried over $MgSO_4$, filtered and the solvent was removed under reduced pressure. The crude product was purified by flash column chromatography (PE/EA = 1:1 → 100 % EA).

Crotonyl-*N*-acetylcysteamine (11a)

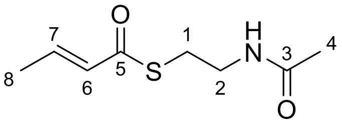

11a

Crotonyl-*N*-acetylcysteamine (**11a**, 285.0 mg, 1.52 mmol, 76 %) was obtained as a colorless solid. The analytical data are consistent with those reported in the literature. [S5] ^{1}H-NMR (400 MHz, $CDCl_3$, $CHCl_3$ = 7.26 ppm): δ 6.89 – 6.83 (m, 1H, H-6), 6.28 (bs, 1H, NH), 6.10 (dq, J = 15.4, 1.6 Hz, 1H, H-7), 3.40 – 3.37 (m, 2H, H-2), 3.05 – 3.01 (m, 2H, H-1), 1.92 (s, 3H, Me-4), 1.86 – 1.83 (m, 3H, Me-8) ppm; ^{13}C-NMR (400 MHz, $CDCl_3$ = 77.16 ppm): δ 190.0 (s, C-5), 170.5 (s, C-3), 141.7 (d, C-7), 129.9 (d, C-6), 39.7 (t, C-2), 28.1 (t, C-1), 23.1 (q, C-4), 18.0 (q, C-8); m.p. 60 °C; ref. [S6] 61.5 – 62 °C; HRMS-ESI *m/z* for $C_8H_{13}NO_2SNa$ [M+Na]+ calc. 210.0565, found: 210.0565.

(*E*)-2-Methylbut-2-enoyl-*N*-acetylcysteamine (11b)

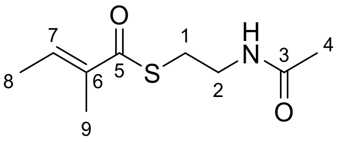

11b

(*E*)-2-Methylbut-2-enoyl-*N*-acetylcysteamin (**11b**, 292.5 mg, 1.45 mmol, 73 %) was obtained as a colorless oil. The analytical data are consistent with those reported in the literature.[S2] ^{1}H-NMR (400 MHz, $CDCl_3$, $CHCl_3$ = 7.26 ppm): δ 6.85 (dq, J = 6.9, 1.1 Hz, 1H, H-7), 6.04 (bs, 1H, NH), 3.42 (dt, J = 6.1, 6.0 Hz 2H, H-2), 3.04 (t, J = 6.4 Hz, 2H, H-1), 1.95 (s, 3H, Me-4), 1.85 (s, 3H, Me-9), 1.82 (d, J = 6.9 Hz, 3H, Me-8) ppm; ^{13}C-NMR (400 MHz, $CDCl_3$ = 77.16 ppm): δ 193.4 (s, C-5), 170.6 (s, C-

3), 136.7 (d, C-7), 136.5 (s, C-6), 39.5 (t, C-2), 28.2 (t, C-1), 22.9 (q, C-4), 14.3 (q, C-9), 12.0 (q, C-8); HRMS-ESI *m/z* for $C_9H_{15}NO_2SNa$ [M+Na]+ calc. 224.0721, found: 224.0721.

(*E*)-2-Methylpent-2-enoyl-*N*-acetylcysteamin (11c)

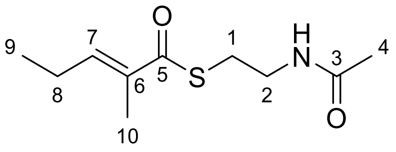

11c

(*E*)-2-Methylpent-2-enoyl-*N*-acetylcysteamine (**11c**, 272.0 mg, 1.26 mmol, 63 %) was obtained as a colorless oil. The analytical data are consistent with those reported in the literature.[S7] ^{1}H-NMR (400 MHz, $CDCl_3$, $CHCl_3$ = 7.26 ppm): δ 6.74 (dt, J = 7.5, 1.1 Hz, 1H, H-7), 6.00 (bs, 1H, NH), 3.43 (q, J = 6.1 Hz, 2H, H-2), 3.05 (t, J = 6.4 Hz, 2H, H-1), 2.25 – 2.18 (m, 2H, H-8), 1.95 (s, 3H, Me-4), 1.86 (s, 3H, Me-10), 1.06 (t, J = 7.5 Hz, 3H, Me-9) ppm; ^{13}C-NMR (400 MHz, $CDCl_3$ = 77.16 ppm): δ 194.1 (s, C-5), 170.8 (s, C-3), 143.6 (d, C-7), 135.3 (s, C-6), 40.0 (t, C-2), 28.3 (t, C-1), 23.2 (q, C-4), 22.2 (t, C-8), 13.0 (q, C-9), 12.4 q, C-10); HRMS-ESI *m/z* for $C_{10}H_{17}NO_2SNa$ $[M+Na]^+$ calc. 238.0878, found: 238.0876.

3.2 3-Hydroxyanilines

3-Amino-5-methylphenol (33)

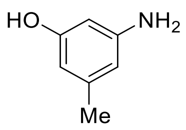

32

5-Methylresorcinol (2.1 g, 16.92 mmol, 1.0 eq.) was dissolved in water (10 mL) and ammonium chloride (1.5 g, 28.04 mmol, 1.7 eq.) and aqueous ammonia (28 %, 5.8 mL) were added. The mixture was heated in an autoclave to 180 °C and stirred for 17 h. The mixture was extracted with ethyl acetate (3 x 15 mL) and the combined organic layers were dried over $MgSO_4$, filtered and the solvent removed under reduced pressure. The crude product was purified by flash column chromatography (PE/EA = 3:1) to yield 3-amino-5-methylphenol (**33**) as a brown solid (0.96 g, 4.02 mmol, 52 % brsm). The analytical data are consistent with those reported in the literature.[S8] ^{1}H-NMR (400 MHz, DMSO-d6, DMSO = 2.50 ppm): δ 8.69 (s, 1H, OH), 5.83 – 5.82 (m, 1H, HAr), 5.81 – 5.80 (m ,1H, HAr), 5.76 – 5.75 (m, 1H, HAr), 4.77 (s, 2H, NH2), 2.04 (s, 3H, Me) ppm; ^{13}C-NMR (400 MHz, DMSO-d6 = 39.52 ppm): δ 158.0 (s, CAr), 149.5 (s, CAr), 138.5 (s, CAr), 106.3 (d, CAr), 104.4 (d, CAr), 98.4 (d, CAr), 21.3 (q, Me); m.p. 137 °C; ref. [S8] 138 °C.

3.3 Total synthesis of seco-acid derivatives 26, 28 and 30

3.3.1 Total synthesis of western fragment 19

***tert*-Butyl {3-[(*tert*-butyldiphenylsilyl)oxy]-5-(iodomethyl)phenyl}carbamate (S5)**

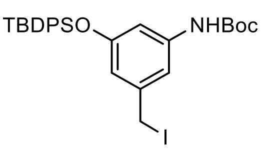

S5

PPh_3 (9.73 g, 37.0 mmol, 1.2 eq.) and imidazole (2.53 g, 37.0 mmol, 1.2 eq.) were dissolved in CH_2Cl_2 (300 mL) at room temperature. The reaction was cooled to 0 °C and iodine (9.39 g, 37.0 mmol, 1.2 eq.) was added in the absence of light. Stirring was continued at this temperature for 30 minutes. Benzylic alcohol **37** (14.7 g, 30.8 mmol, 1.0 eq.) dissolved in CH_2Cl_2 (50 mL) was added via canula. The reaction was stirred at 0 °C for 4 h. Silica gel was added and the solvent was removed under reduced pressure. The crude product was purified by flash column chromatography (PE/EtOAc = 10:1 → 6:1) to yield benzylic iodide **S5** (16.29 g, 27.72 mmol, 90%) as a pale-yellow oil. The analytical data are consistent with those reported in the literature.

R_f = 0.7 (PE/EtOAc = 4:1); ^{1}H-NMR (400 MHz, $CDCl_3$, $CHCl_3$ = 7.26 ppm): δ 7.71 – 7.69 (m, 4H, TBDPS), 7.43 – 7.36 (m, 6H, TBDPS), 7.12 (bs, 1H, C_{Ar}), 6.55 – 6.54 (m, 1H, C_{Ar}), 6.40 – 6.39 (m, 1H, C_{Ar}), 6.25 (bs, 1H, NH), 4.18 (s, 2H, CH_2I), 1.48 (s, 9H, Boc), 1.09 (s, 9H, TBDPS) ppm; ^{13}C-NMR (400 MHz, $CDCl_3$, $CDCl_3$ = 77.16 ppm): δ 156.2 (s, C_{Ar}), 152.5 (s, Boc), 140.9 (s, C_{Ar}), 139.5 (s, C_{Ar}), 135.6 (d, 4C, TBDPS), 132.7 (s, 2C, TBDPS), 130.1 (d, 2C, TBDPS), 128.0 (d, 4C, TBDPS), 115.1 (d,

C$_{Ar}$), 111.7 (d, C$_{Ar}$), 109.5 (d, C$_{Ar}$), 80.8 (s, Boc), 28.4 (q, 3C, Boc), 26.6 (q, 3C, TBDPS), 19.6 (s, TBDPS), 5.5 (t, CH_2I) ppm; HRMS-ESI *m/z* for $C_{28}H_{34}INO_3SiNa$ [M+Na]$^+$ calc. 610.1250, found 610.1248.

tert-Butyl {3-[(_R_)-3-((_S_)-4-benzyl-2-oxooxazolidin-3-yl)-2-methyl-3-oxopropyl]-5-[(_tert_-butyldiphenylsilyl)oxy]phenyl}carbamate (15)

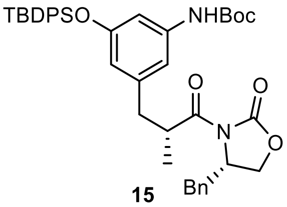
15

DIPA (1.29 mL, 9.24 mmol, 1.7 eq.) was dissolved in THF (25 mL) and cooled to –78 °C. *n*-BuLi (2.5 M in hexane, 3.69 mL, 9.24 mmol, 1.7 eq.) was added slowly and stirring was continued at –78 °C for 5 min. (*S*)-Oxazolidinone **20** (2.16 g, 9.24 mmol, 1.7 eq.) was added as a solution in THF (20 mL) via cannula. Stirring was continued for 15 min at –78 °C. Iodide **S5** (3.19 g, 5.44 mmol, 1.0 eq.) was added as a solution in THF (30 mL) via cannula. The reaction was stirred at –78 °C for 15 min and then slowly warmed to –35 °C over 2.5 h. The reaction was terminated by the addition of an aqueous saturated NH_4Cl solution. The phases were separated and the aqueous phase was extracted with EtOAc (6 x 75 mL). The combined organic phases were dried over $MgSO_4$, filtered and the solvent was removed under reduced pressure. The crude product was purified by flash column chromatography (PE/EtOAc = 10:1 → 2:1) to yield **15** (3.14 g, 4.53 mmol, 83%) as a yellow foam.

R_f = 0.2 (PE/EtOAc = 4:1); ^{1}H-NMR (400 MHz, $CDCl_3$, $CHCl_3$ = 7.26 ppm): δ 7.71 – 7.68 (m, 4H, TBDPS), 7.43 – 7.24 (m, 9H, TBDPS, Bn), 7.12 – 7.10 (m, 2H, Bn), 6.86 (bs, 1H, H$_{Ar}$), 6.73 (bs, 1H, NH), 6.29 (m, 1H, H$_{Ar}$), 6.25 (bs, 1H, H$_{Ar}$), 4.67 – 4.61 (m, 1H, C*H*Bn), 4.18 – 4.10 (m, 2H, CH_2CHBn), 3.86 – 3.81 (m, 1H, C-14), 3.16 (dd, *J* = 13.4, 3.2 Hz, 1H, Bn), 2.93 (dd, *J* = 13.2, 6.2 Hz, 1H, C-15), 2.60 (dd, *J* = 13.4, 9.5 Hz, 1H, Bn), 2.33 (dd, *J* = 13.2, 8.4 Hz, 1H, C-15), 1.45 (s, 9H, Boc), 1.07 (s, 9H, TBDPS), 0.93 (d, *J* = 6.7 Hz, 3H, Me-14) ppm; ^{13}C-NMR (400 MHz, $CDCl_3$, $CDCl_3$ = 77.16 ppm): δ 176.6 (s, C-13), 156.1 (s, C$_{Ar}$), 153.1 (s, Aux), 152.5 (s, Boc), 140.9 (s, C$_{Ar}$), 139.3 (s, C$_{Ar}$), 135.7 (d, 4C, TBDPS), 135.6 (s, Bn), 133.0 (s, 2C, TBDPS), 132.9 (d, Bn), 129.9 (d, 2C, TBDPS), 129.5 (d, Bn), 129.0 (d, Bn), 127.9 (d, 4C, TBDPS), 127.8 (d, Bn), 127.4 (d, Bn), 115.8 (d, C$_{Ar}$), 112.2 (d, C$_{Ar}$), 108.2 (d, C$_{Ar}$), 80.5 (s, Boc), 66.1 (t, *C*H_2O), 55.3 (d, *C*HN), 39.7 (s, Bn), 39.5 (d, C-14), 38.0 (t, C-15), 28.4 (q, 3C, Boc), 26.6 (q, 3C, TBDPS), 19.6 (s, TBDPS), 16.2 (q, Me-14) ppm; $[\alpha]_D^{20}$ = –2.4 (*c* = 1.0, $CHCl_3$; HRMS-ESI *m/z* for $C_{41}H_{48}N_2O_6SiNa$ [M+Na]$^+$ calc. 715.3179, found 715.3179.

(2_R_)-3-[3-(_tert_-Butoxycarbonylamino)-5-(_tert_-butyldiphenylsiloxy)-phenyl]-2-methyl-propan-1-ol (S6)

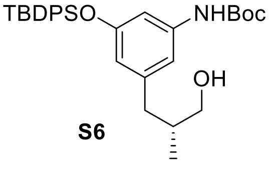
S6

The oxazolidinone **15** (10.2 g, 14.7 mmol, 1.0 eq.) was dissolved in Et_2O (70 mL) under argon atmosphere, added with water (0.23 mL, 14.7 mmol, 1.0 eq.) and cooled to 0 °C. Then lithium borohydride (2 M solution in THF) (16.2 mL, 32.4 mmol, 2.2 eq.) was slowly added and the reaction mixture was stirred for 1.5 h at 0 °C. The combined organic extracts were washed with aqueous NaCl solution, dried over $MgSO_4$ and concentrated under reduced pressure. The crude product was purified by flash chromatography (PE:EtOAc = 10:1 → 2:1) to give alcohol **S6** (5.95 g, 11.5 mmol, 78%) as a colorless foam; R_f = 0.4 (PE:EA= 2:1). ^{1}H-NMR (400 MHz, $CDCl_3$, $CHCl_3$ = 7.26 ppm) δ 7.71-7.69 (m, 4H, SiPh), 7.43-7.37 (m, 6H, SiPh), 6.80 (bs, 1H, Ph), 6.68 (dd, *J* = 2.1, 2.1 Hz, 1H, Ph), 6.25 (bs, 1H, NH), 6.09 (dd, *J* = 1.7, 1.7 Hz, 1H, Ph), 3.26 (dd, *J* = 10.5, 5.6 Hz, 1H, C*H*$_2$OH), 3.17 (dd, *J* = 10.5, 5.9 Hz, 1H, C*H*$_2$OH), 2.39 (dd, *J* = 13.3, 6.9 Hz, 1H, ArCH_2), 2.15 (dd, *J* = 13.3, 7.7 Hz, 1H, ArCH_2), 1.66-1.58 (m, 1H, C*H*CH_3), 1.48 (s, 9H, *t*-Bu), 1.25 (bs, CH_2OH), 1.07 (s, 9H, *t*-SiBu), 0.70 (d, *J* = 6.8 Hz, 3H, CHC*H*$_3$) ppm; ^{13}C-NMR (100 MHz, $CDCl_3$, $CDCl_3$ = 77.16 ppm) δ 155.8 (s, C-Ar), 152.5 (s, NCOO), 142.3 (s, C-Ar), 139.1 (s, C-Ar), 135.5 (d, C-Ar), 132.9 (s, C-Ar), 129.8 (d, C-Ar), 127.7 (d, C-Ar), 115.3 (d, C-Ar), 111.9 (d, C-Ar), 107.5 (d, C-Ar), 80.3 (s, *t*-Bu), 67.1 (t, CH_2), 39.4 (t, CH_2),

37.3 (d, CH), 28.3 (q, *t*-Bu), 26.4 (q, Si*t*-Bu), 19.4 (s, Si*t*-Bu), 16.4 (q, CH_3) ppm; HRMS (ESI) *m/z* for $C_{31}H_{41}NO_4SiNa$ $[M+Na]^+$: caculated: 542.2703, found: 542.2699; $[\alpha]^{20}_D$= +4.9° (*c* = 1.0, $CHCl_3$).

(2*R*)-3-[3-(*tert*-Butoxycarbonylamino)-5-(*tert*-butyldiphenylsiloxy)-phenyl]-2-methyl-propanal (S7)

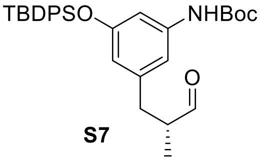

S7

Alcohol **S6** (5.90 g, 11.4 mmol, 1.0 eq.) was dissolved in CH_2Cl_2 (170 mL), cooled to 0 °C, and $NaHCO_3$ (1.16 g, 13.6 mmol, 1.2 eq.) was added followed by the Dess-Martin periodinane reagent (5.76 g, 13.6 mmol, 1.2 eq.).After 1 h, the reaction was terminated by the addition of an aqueous Na_2SO_3-solution and the aqueous phase was extracted three times with CH_2Cl_2. The combined organic phases were washed with brine, dried over $MgSO_4$, and concentrated under reduced pressure. Aldehyde **S7** (5.80 g, 11.2 mmol, 99%) was obtained as a colorless foam; Rf = 0.6 (PE:EA = 2:1). The aldehyde **S7** was directly used in the next reaction.

Ethyl (4*R*)-5-[3-(*tert*-Butoxycarbonylamino)-5-(*tert*-butyldiphenylsiloxy)-phenyl]-2-methyl-pent-2-enoate (16)

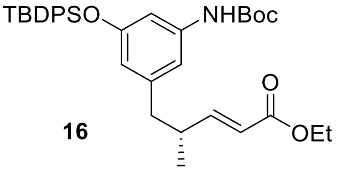

16

Aldehyde **S7** (5.80 g, 11.2 mmol, 1.0 eq.) was dissolved in $CHCl_3$ (35 mL) and phosphorylide Ph_3P=$CHCO_2Et$ (5.85 g, 16.8 mmol, 1.5 eq.) was added. The reaction mixture was heated to 50 °C and stirred for an additional 15 h at 50 °C. The solvent was removed under reduced pressure, the crude product was purified by flash chromatography (PE:EA= 15:1 → 4:1), and ester **16** (6.40 g, 10.9 mmol, 97%) was obtained as a colorless foam; Rf = 0.3 (PE:EA= 5:1). ^{1}H-NMR (400 MHz, $CDCl_3$, $CHCl_3$ = 7.26 ppm) δ 7.72-7.68 (m, 4H, SiPh), 7.44-7.34 (m, 6H, SiPh), 6.81 (dd, *J* = 15.7, 7.0 Hz, 1H, 3-H), 6.79 (bs, 1H, NH), 6.64 (s, 1H, Ph), 6.27 (bs, 1H, Ph), 6.09 (s, 1H, Ph), 5.63 (dd, *J* = 15.7, 0.6 Hz, 1H, 2-H), 4.16 (q, *J* = 7.1 Hz, 2H, OCH_2), 2.52-2.48 (m, 1H, 5-Ha), 2.27 (dq, *J* = 6.8, 6.7 Hz, 1H, 4-H), 2.23-2.17 (m, 1H, 5-Hb), 1.48 (s, 9H, *t*-Bu), 1.27 (t, *J* = 7.1 Hz, 3H, OCH_2CH_3), 1.07 (s, 9H, Si*t*-Bu), 0.78 (d, *J* = 6.7 Hz, 3H, 6-H) ppm; ^{13}C-NMR (100 MHz, $CDCl_3$, $CDCl_3$ = 77.16 ppm) δ 166.9 (s, COOEt), 156.1 (s, C- Ar), 153.6 (d, C-3), 152.6 (s, NHCOO), 141.5 (s, C-Ar), 139.2 (s, C-Ar), 133.0 (d, C-Ar), 133.0 (s, C-Ar), 130.0 (d, C-Ar), 130.0 (d, C- Ar), 128.7 (d, C-Ar), 119.8 (d, C-2), 115.3 (d, C-Ar), 112.0 (d, C-Ar), 107.9 (d, C-Ar), 80.5 (s, *t*-Bu), 60.2 (t, OCH_2), 42.2 (t, C-5), 37.9 (d, C-4), 28.4 (q, *t*-Bu), 26.6 (q, Si*t*-Bu), 19.5 (s, Si*t*-Bu), 18.5 (q, C-6), 14.4 (q, OCH_2CH_3) ppm; HRMS (ESI) *m/z* for $C_{35}H_{45}NO_5SiNa$ $[M+Na]^+$: calculated: 610.2965, found: 610.2962; $[\alpha]^{20}_D$ = -19.1° (*c* = 1.2, $CHCl_3$).

(4*R*)-5-[3-(*tert*-Butoxycarbonylamino)-5-(*tert*-butyldiphenylsiloxy)-phenyl]-4-methyl-pent-2-enol (S8)

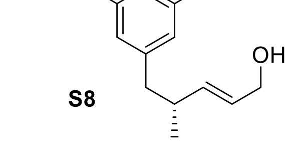

S8

Ester **S7** (6.35 g, 10.8 mmol, 1.0 eq.) was dissolved in CH_2Cl_2 (100 mL) under argon atmosphere, cooled to -78 °C, and slowly mixed with DIBAL-H (1.2 M solution in toluene) (22.5 mL, 27.0 mmol, 2.5 eq.). The reaction mixture was stirred at -78 °C for 5 h and then warmed to room temperature. The reaction was terminated by addition of an aqueous Na-K tartrate solution and the reaction mixture was stirred overnight at room temperature. The aqueous phase was extracted three times with CH_2Cl_2. The combined organic extracts were washed with brine, dried over $MgSO_4$ and concentrated under reduced pressure. The crude product was purified by flash chromatography (PE:EA= 10:1 → 2:1) to give alcohol **S8** (4.50 g, 8.25 mmol, 76%) as a colorless foam; R_f = 0.4 (PE:EA= 2:1). ^{1}H-NMR (400 MHz, $CDCl_3$, $CHCl_3$ = 7.26 ppm) δ 7.71-7.69 (m, 4H, SiPh), 7.43-7.33 (m, 6H, SiPh), 6.91 (s, 1H, Ph), 6.46 (m, 1H, Ph), 6.23 (bs, 1H, NH), 6.14 (m, 1H, Ph), 5.50 (dd, *J* = 15.5, 7.0 Hz, 1H, 3-H), 5.39 (dt, *J* = 15.5, 5.5 Hz, 1H, 2-H), 3.99 (t, *J* = 5.5 Hz, 2H, 1-H), 2.39 (dd, *J* = 13.1, 7.3 Hz, 1H,

5-Ha), 2.28 (dd, J = 13.1, 6.7 Hz, 1H, 5-Hb), 2.16 (m, 1H, 4-H), 1.73 (bs, 1H, CH_2OH), 1.47 (s, 9H, *t*-Bu), 1.09 (s, 9H, Si*t*-Bu), 0.80 (d, J = 6.8 Hz, 3H, 5-Me) ppm; ^{13}C-NMR (100 MHz, $CDCl_3$, $CDCl_3$ = 77.16 ppm) δ 155.8 (s, C-Ar), 152.9 (s, NHCOO), 142.4 (s, C-Ar), 138.7 (s, C-Ar), 138.0 (d, C-3), 135.6 (d, C-Ar), 133.0 (s, C-Ar), 130.0 (d, C-Ar), 127.8 (d, C-2), 115.7 (d, C-Ar), 112.9 (d, C-Ar), 107.7 (d, C-Ar), 80.6 (s, *t*-Bu), 63.9 (t, C-1), 43.4 (t, C-5), 37.8 (d, C-4), 28.5 (q, *t*-Bu), 26.6 (q, Si*t*-Bu), 19.7 (q, 5-Me), 19.6 (s, Si*t*-Bu) ppm; HRMS (ESI) *m/z* for $C_{33}H_{44}NO_4Si$ $[M+H]^+$: calculated: 546.3040, found: 546.3031; $[\alpha]^{20}_D$ = -10.7° (*c* = 1.0, $CHCl_3$).

tert-Butyl {3-[(_tert_-butyldiphenylsilyl)oxy]-5-[(_R_)-2-[(2_R_,3_R_)-3-(hydroxymethyl)oxiran-2-yl]propyl]phenyl}carbamate (17)

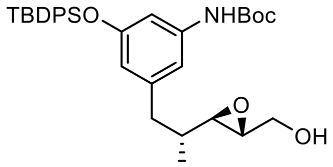

17

Freshly activated molecular sieves (4 Å) were suspended in CH_2Cl_2 (40 mL) and D-(-)-DET (0.82 mL, 4.76 mmol, 1.3 eq.) was added. The reaction was cooled to – 20 °C and Ti(O*i*Pr)4 (1.29 mL, 4.39 mmol, 1.2 eq) and *t*-BuOOH (5.0 – 6.0 M in decane, 2.93 mL, 14.65 – 17.58 mmol, 4.0 eq.) were added subsequently. Stirring was continued at this temperature for 1 h. Allylic alcohol **S8** (2.00 g, 3.66 mmol, 1.0 eq.) as a solution in CH_2Cl_2 (10 mL) was slowly added to the reaction mixture and stirring was continued at – 20 °C for 42 h. The reaction was terminated by addition of a EDTE (1.09 g, 4.61 mmol, 1.26 eq.; EDTE= *N,N,N',N'*-tetrakis(2-hydroxyethyl)ethylenediamine; 1.05 eq. relative to the amount of $Ti(OiPr)_4$). The mixture was heated to 55 °C for 15 min and after cooling to room temperature, the mixture was diluted with aqueous NH4OH and CH_2Cl_2. The two clear phases were separated and the aqueous layer was extracted with CH_2Cl_2 (3 x 20 mL). The combined organic layers were dried over $MgSO_4$, filtered and the solvent was removed under reduced pressure. The crude product was purified by flash column chromatography (PE/EtOAc = 10:1 → 2:1) to yield epoxide **17** (1.96 g, 3.48 mmol, 96%, d.r. > 10:1) as a colourless foam. R*f* = 0.3 (PE/EtOAc = 4:1); ^{1}H-NMR (400 MHz, $CDCl_3$, $CHCl_3$ = 7.26 ppm): δ 7.71 – 7.69 (m, 4H, TBDPS), 7.43 – 7.34 (m, 6H, TBDPS), 6.85 (bs, 1H, HAr), 6.63 – 6.62 (m, 1H, HAr), 6.29 (bs, 1H, NH), 6.12 – 6.11 (m, 1H, HAr), 3.81 (dd, J = 12.6, 2.7 Hz, 1H, H-11), 3.56 (dd, J = 12.6, 4.2 Hz, 1H, H-11'), 2.86 – 2.84 (m, 1H, H-12), 2.70 (dd, J = 6.8, 2.3 Hz, 1H, H-13), 2.62 (dd, J = 13.4, 5.3 Hz, 1H, H-15), 2.22 (dd, J = 13.3, 8.9 Hz, 1H, H-15'), 1.53 – 1.15 (m, 1H, H-14), 1.48, (s, 9H, Boc), 1.08 (s, 9H, TBDPS), 0.59 (d, J = 6.8 Hz, 3H, Me-14) ppm; ^{13}C-NMR (400 MHz, $CDCl_3$, $CDCl_3$ = 77.16 ppm): δ 156.0 (s, CAr), 152.7 (s, Boc), 141.4 (s, CAr), 139.1 (s, CAr), 135.6 (d, CAr), 135.6 (d, CAr), 133.0 (s, CAr), 133.0 (s, CAr), 130.0 (d, CAr), 130.0 (d, CAr), 127.9 (d, CAr), 127.9 (d, CAr), 115.6 (d, CAr), 112.4 (d, CAr), 107.9 (d, CAr), 80.6 (s, Boc), 61.9 (t, C-11), 59.9 (d, C-13), 57.2 (d, C-12), 40.1 (t, C-15), 36.4 (d, C-14), 28.5 (q, Boc), 26.6 (q, TBDPS), 19.6 (s, TBDPS), 14.8 (q, Me-14) ppm; $[\alpha]_D^{20}$ = + 5.6 (*c* = 1.2, CH_2Cl_2; HRMS-ESI *m/z* for $C_{33}H_{43}NO_5SiNa$ [M+Na]+ calc. 584.2808, found: 584.2807.

(4_R_, 2_S_)-5-[3-(_tert_-Butoxycarbonylamino)-5-(_tert_-butyldiphenylsiloxy)-phenyl]-4-methyl-1,2-pentanediol (S9)

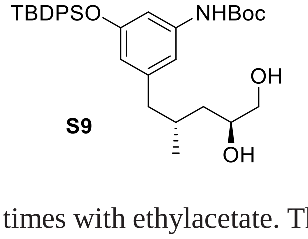

S9

Epoxide **17** (7.16 g, 12.8 mmol, 1.0 eq.) was dissolved in Et_2O (450 mL) under argon atmosphere, cooled to 0 °C and mixed with DIBAL-H (1.2 M solution in toluene) (53.1 mL, 63.7 mmol, 5.0 eq.). The reaction mixture was warmed to room temperature and stirred for an additional 5 h at room temperature. Na-K-tartrate solution was added and the reaction mixture was stirred overnight at room temperature. The aqueous phase was extracted three times with ethylacetate. The combined organic extracts were washed with brine, dried over $MgSO_4$ and concentrated under reduced pressure. The crude product was purified by flash chromatography (PE:EtOAc = 1:1 → 1:4) to give diol **S9** (6.09 g, 10.8 mmol, 85%) as a colorless foam; R_f = 0.5 (PE:EA

= 1:4). ^{1}H-NMR (400 MHz, $CDCl_3$, $CHCl_3$ = 7.26 ppm) δ 7.71-7.69 (m, 4H, SiPh), 7.41-7.34 (m, 6H, SiPh), 6.80 (s, 1H, Ph), 6.63 (m, 1H, Ph), 6.26 (bs, 1H, NH), 6.11 (m, 1H, Ph), 3.72-3.65 (m, 1H, 2-H), 3.52 (ddd, *J* = 10.8, 5.8, 3.3 Hz, 1H, 1-Ha), 3.30 (ddd, *J* = 10.8, 7.3, 4.0 Hz, 1H, 1-Hb), 2.31 (dd, *J* = 13.3, 6.6 Hz, 1H, 5-Ha), 2.19 (dd, *J* = 13.3, 7.8 Hz, 1H, 5-Hb), 1.86 (dd, *J* = 5.8, 4.0 Hz, 1H, 1-OH), 1.77 (d, *J* = 4.4 Hz, 1H, 2-OH), 1.73-1.64 (m, 1H, 4-H), 1.47 (s, 9H, *t*-Bu), 1.31 (ddd, *J* = 13.8, 9.3, 4.6 Hz, 1H, 3-Ha), 1.07 (s, 9H, Si*t*-Bu), 0.97 (ddd, *J* = 13.8, 9.7, 3.9 Hz, 1H, 3-Hb), 0.69 (d, *J* = 6.4 Hz, 3H, 5-Me) ppm; ^{13}C-NMR (100 MHz, $CDCl_3$, $CDCl_3$ = 77.16 ppm) δ 155.9 (s, C-Ar), 152.7 (s, NHCOO), 142.6 (s,C-Ar), 139.0 (s, C-Ar), 135.6 (d, C-Ar), 133.0 (s, C-Ar), 129.9 (d, C-Ar), 127.8 (d, C-Ar), 115.5 (d, C-Ar), 112.3 (d, C-Ar), 107.9 (d, C-Ar), 80.5 (s, *t*-Bu), 70.1 (d, C-2), 67.5 (t, C-1), 44.0 (t, C-5), 39.7 (t, C-3), 30.8 (d, C-4), 28.4 (q, *t*-Bu), 26.6 (q, Si*t*-Bu), 19.5 (s, Si*t*-Bu), 14.7 (q, 5-Me) ppm; HRMS (ESI) *m/z* for $C_{33}H_{45}NO_5SiNa$ $[M+Na]^+$: calculated: 586.2965, found: 586.2964; $[\alpha]^{20}_D$ = -10.1° (*c* = 1.0, $CHCl_3$).

tert-Butyl {3-[(2*R*,4*S*)-5-[(*tert*-butyldimethylsilyl)oxy]-4-hydroxy-2-methylpentyl]-5-[(*tert*-butyldiphenylsilyl)oxy]phenyl}carbamate (S10)

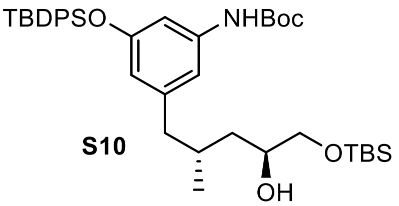

S10

Diol **S9** (1.81 g, 3.20 mmol, 1.0 eq.) was dissolved in CH_2Cl_2 (100 mL) and cooled to 0 °C. 2,6-Lutidine (372 µL, 3.20 mmol, 1.0 eq.) was added followed by dropwise addition of TBSOTf (736 µL, 3.20 mmol, 1.0 eq.). The reaction was stirred at 0 °C for 30 min and was terminated by the addition of a saturated aqueous NH_4Cl solution. The phases were separated and the aqueous phase was extracted with CH_2Cl_2 (4 x 20 mL). The combined organic phases were washed with brine, dried over $MgSO_4$, filtered and the solvent was removed under reduced pressure. The crude product was purified by flash column chromatography (PE/EtOAc = 20:1) to yield silyl ether **S10** (1.67 g, 2.46 mmol, 77%, 92% brsm) as a colorless oil. R_f = 0.5 (PE/EtOAc = 4:1); ^{1}H-NMR (400 MHz, $CDCl_3$, $CHCl_3$ = 7.26 ppm): δ 7.72 – 7.70 (m, 4H, TBDPS), 7.43 – 7.34 (m, 6H, TBDPS), 6.78 (bs, 1H, H_{Ar}), 6.66 (s, 1H, H_{Ar}), 6.26 (bs, 1H, NH), 6.15 (s, 1H, H_{Ar}), 3.70 – 3.64 (m 1H, H-12), 3.52 (dd, *J* = 9.9, 3.4 Hz, 1H, H-11), 3.31 (dd, *J* = 9.8, 7.6 Hz, H-11'), 2.40 (dd, *J* = 13.3, 6.0 Hz, 1H, H-15), 2.13 (dd, *J* = 13.3, 8.4 Hz, 1H, H-15'), 1.82 – 1.73 (m, 1H, H-14), 1.48 (s, 9H, Boc), 1.37 – 1.31 (m, 1H, H-13), 1.08 (s, 9H, TBDPS), 0.99 – 0.92 (m, 1H, H-13'), 0.90 (s, 9H, TBDPS), 0.67 (d, *J* = 6.6 Hz, 3H, Me-14), 0.07 (s, 6H, TBDPS) ppm; ^{13}C-NMR (400 MHz, $CDCl_3$, $CDCl_3$ = 77.16 ppm): δ 155.9 (s, C_{Ar}), 152.6 (s, Boc), 142.9 (s, C_{Ar}), 139.0 (s, C_{Ar}), 135.7 (d, 4C, TBDPS), 133.1 (s, 2C, TBDPS), 129.9 (d, 2C, TBDPS), 127.9 (d, 4C, TBDPS), 115.6 (d, C_{Ar}), 112.3 (d, C_{Ar}), 107.6 (d, C_{Ar}), 80.4 (s, Boc), 69.7 (d, C-12), 68.0 (t, C-11), 44.3 (t, C-15), 39.6 (t, C-13), 31.0 (d, C-14), 28.5 (q, 3C, Boc), 26.6 (q, 3C, TBDPS), 26.0 (q, 3C, TBS), 19.6 (s, TBDPS), 19.0 (q, Me-14), 18.4 (s, TBS), -5.2 (q, 2C, TBS) ppm; $[\alpha]^{20}_{D} = -2.0$ (*c* = 1.4, CH_2Cl_2; HRMS-ESI *m/z* for $C_{39}H_{59}NO_5Si_2Na$ $[M+Na]^+$ calc. 700.3830, found 700.3834.

(4*R*, 2*S*)-5-[3-(*tert*-Butoxycarbonylamino)-5-(*tert*-butyldiphenylsiloxy)-phenyl]-4-methyl-1-(tert-butyldemethylsiloxy)-2-methoxypentane (S11)

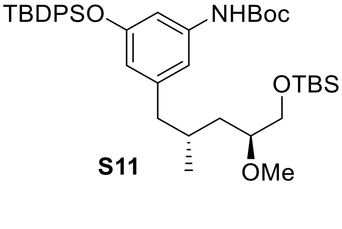

S11

Alcohol **S10** (4.40 g, 6.49 mmol, 1.0 eq.) was dissolved in CH_2Cl_2 (120 mL) under argon atmosphere and 1,8-bis(dimethylamino)-naphthalene (4.87 g, 22.7 mmol, 3.5 eq.) and Me_3OBF_4 (3.08 g, 16.2 mmol, 2.5 eq.) were added. After 1 h, the reaction was terminated by addition of water. The phases were separated and the aqueous phase was extracted three times with CH_2Cl_2. The combined organic extracts were washed with brine, dried over $MgSO_4$ and concentrated under reduced pressure. The crude product was purified by flash chromatography (PE:EA= 15:1 → 4:1) to give the methylated alcohol **S11** (3.98 g, 5.76 mmol,

89%) as a colorless foam; R_f = 0.5 (PE:EtOAc = 4:1). ^{1}H-NMR (400 MHz, $CDCl_3$, $CHCl_3$ = 7.26 ppm) δ 7.72-7.69 (m, 4H, SiPh), 7.43-7.33 (m, 6H, SiPh), 6.75 (s, 1H, Ph), 6.67 (m, 1H, Ph), 6.24 (bs, 1H, NH), 6.15 (m, 1H, Ph), 3.58 (dd, *J* = 10.5, 5.8 Hz, 1H, 1-Ha), 3.46 (dd, *J* = 10.5, 4.6 Hz, 1H, 1-Hb), 3.36 (s, 3H, OMe), 3.25-3.19 (m, 1H, 2-H), 2.42 (dd, *J* = 13.2, 5.5 Hz, 1H, 5-Ha), 2.06 (dd, *J* = 13.2, 9.0 Hz, 1H, 5-Hb), 1.75-1.66 (m, 1H, 4-H), 1.48 (s, 9H, *t*-Bu), 1.38-1.31 (m, 1H, 3-Ha), 1.08 (s, 9H, Si*t*-Bu), 0.98-0.93 (m, 1H, 3-Hb), 0.89 (s, 9H, Si*t*-Bu), 0.62 (d, *J* = 6.5 Hz, 3H, 5-Me), 0.05 (s, 6H, SiMe) ppm; ^{13}C-NMR (100 MHz, $CDCl_3$, $CDCl_3$ = 77.16 ppm) δ 155.9 (s, C-Ar), 152.6 (s, NHCOO), 143.1 (s,C-Ar), 139.0 (s, C-Ar), 135.7 (d, C-Ar), 133.1 (s, C-Ar), 129.9 (d, C-Ar), 127.8 (d, C-Ar), 115.6 (d, C-Ar), 112.3 (d, C-Ar), 107.6 (d, C-Ar), 80.4 (s, *t*-Bu), 80.0 (d, C-2), 66.1 (t, C-1), 58.2 (q, OMe), 44.4 (t, C-5), 39.2 (t, C-3), 31.2 (d, C-4), 28.5 (q, *t*-Bu), 26.6 (q, Si*t*-Bu), 26.1 (q, Si*t*-Bu) 19.6 (s, Si*t*-Bu), 19.0 (q, 5-Me), 18.4 (s, Si*t*-Bu), -5.2 (q, SiMe) ppm; $[\alpha]^{20}_D$ = -7.4° (*c* = 1.0, $CHCl_3$).

(4*R*, 2*S*)-5-[3-(*tert*-Butoxycarbonylamino)-5-(*tert*-butyldiphenylsiloxy)-phenyl]-4-methyl-2-methoxypentane (18)

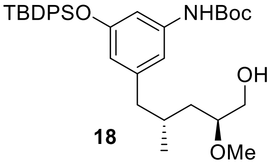

18

Alcohol **S11** (3.98 g, 5.76 mmol, 1.0 eq.) was dissolved in CH_2Cl_2 (40 mL) under argon atmosphere and acetonitrile (40 mL) and $LiBF_4$ (1.62 g, 17.3 mmol, 3.0 eq.) were added. After 48 h, the reaction was terminated by addition of waterThe phases were separated and the aqueous phase was extracted three times with CH_2Cl_2. The combined organic extracts were washed with brine, dried over $MgSO_4$ and concentrated under reduced pressure. The crude product was purified by flash chromatography (PE:EA= 10:1 → 2:1) followed by preparative HPLC (C18-P) (H_2O:MeOH = 80:20 {5 min}, gradient H_2O:MeOH = 80:20 → 0:100 {85 min}, H_2O:MeOH = 0:100 {10 min}, 15 mL/min) (t_R = 85. 0 min) was purified and the methylated alcohol **18** (2.50 g, 4.32 mmol, 75%) was obtained as a colorless foam; R_f = 0.3 (PE:EA= 2:1).

^{1}H-NMR (400 MHz, $CDCl_3$, $CHCl_3$ = 7.26 ppm) δ 7.71-7.69 (m, 4H, SiPh), 7.41-7.34 (m, 6H, SiPh), 6.77 (s, 1H, Ph), 6.66 (m, 1H, Ph), 6.26 (bs, 1H, NH), 6.11 (m, 1H, Ph), 3.61-3.58 (m, 1H, 1-Ha), 3.40-3.35 (m, 1H, 1-Hb), 3.30 (s, 3H, OMe), 3.28-3.22 (m, 1H, 2-H), 2.38 (dd, *J* = 13.2, 5.8 Hz, 1H, 5-Ha), 2.07 (dd, *J* = 13.2, 8.7 Hz, 1H, 5-Hb), 1.85 (br. s, 1H, 1-OH), 1.66-1.59 (m, 1H, 4-H), 1.48 (s, 9H, *t*-Bu), 1.50-1.46 (m, 1H, 3-Ha), 1.07 (s, 9H, Si*t*-Bu), 1.10-1.05 (m, 1H, 3-Hb), 0.69 (d, *J* = 6.8 Hz, 3H, 5-Me) ppm; ^{13}C-NMR (100 MHz, $CDCl_3$, $CDCl_3$ = 77.16 ppm) δ 155.9 (s, C-Ar), 152.7 (s, NHCOO), 142.6 (s, C-Ar), 139.0 (s, C-Ar), 135.6 (d, C-Ar), 133.0 (s, C-Ar), 129.9 (d, C-Ar), 127.8 (d, C-Ar), 115.5 (d, C-Ar), 112.3 (d, C-Ar), 107.9 (d, C-Ar), 80.5 (s, *t*-Bu), 79.6 (d, C-2), 64.3 (t, C-1), 57.0 (q, OMe), 44.0 (t, C-5), 37.9 (t, C-3), 31.4 (d, C-4), 28.4 (q, *t*-Bu), 26.6 (q, Si*t*-Bu), 19.5 (s, Si*t*-Bu), 19.5 (q, 5-Me) ppm; HRMS (ESI) *m/z* for $C_{37}H_{48}NO_5Si$ $[M+H]^+$: calculated: 578.3454, found: 578.3467; $[\alpha]^{20}_D$ = +6.1° (*c* = 1.0, CH_2Cl_2).

(4*R*, 2*S*)-5-[3-(*tert*-Butoxycarbonylamino)-5-(*tert*-butyldiphenylsiloxy)-phenyl]-4-methyl-2-methoxy-1-pentanal (S12)

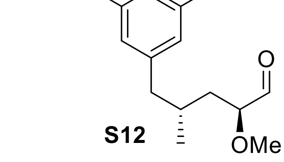

S12

Alcohol **18** (0.15 g, 0.25 mmol, 1.0 eq.) was dissolved in CH_2Cl_2 (20 mL), cooled to 0 °C, and $NaHCO_3$ (0.03 g, 0.30 mmol, 1.2 eq.) and the Dess-Martin periodinane reagent (0.13 g, 0.30 mmol, 1.2 eq.) were added. After 1 h at room temperature, the reaction was terminated by addition of an aqueous Na_2SO_3 solution. The phases were separated and the aqueous phase was extracted three times with CH_2Cl_2. The combined organic phases were washed with aqueous NaCl solution, dried over $MgSO_4$, concentrated under reduced pressure, and aldehyde **S12** (0.15 g, 0.25 mmol, 99%) was obtained as a colorless foam; Rf = 0.8 (PE:EA= 2:1). The aldehyde **S12** was directly reused without characterization.

(3*S*, 4*R*, 5*S*, 7*R*)-8-[3-*tert*-Butoxycarbonylamino-5-(*tert*-butyldiphenylsiloxy)-phenyl]-4-hydroxy-5-methoxy-3,7-dimethyl-oct-2-ene (19)

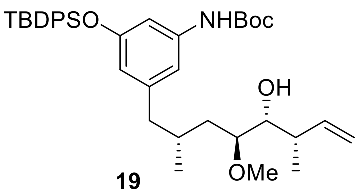

19

Z-Crotylboronate **21** (0.18 g, 0.61 mmol, 2.5 eq.) was dissolved in toluene (2.0 mL) under argon atmosphere. Molecular sieves 4Å (0.10 g) was then added, the reaction mixture was stirred at room temperature for 20 min, and then cooled to -78 °C. Aldehyde **S12** (0.14 g, 0.24 mmol, 1.0 eq.) was dissolved in toluene (1.0 mL) and slowly added to the reaction mixture. After 20 h, the reaction was terminated by addition of an aqueous NaOH solution (1.0 M). The reaction mixture was heated to room temperature and stirred for 1 h at this temperature. The molecular sieves were filtered off over Celite™ and the aqueous phase was extracted three times with Et_2O. The combined organic extracts were washed with brine, dried over $MgSO_4$ and concentrated under reduced pressure. The crude product was purified by flash chromatography (PE:EA= 10:1 → 4:1) to afford alkene **19** (0.15 g, 0.24 mmol, 97%) as a colorless foam; R_f = 0.4 (PE:EA= 5:1). ^{1}H-NMR (400 MHz, $CDCl_3$, $CHCl_3$ = 7.26 ppm) δ 7.72-7.69 (m, 4H, SiPh), 7.42-7.34 (m, 6H, SiPh), 6.77 (s, 1H, Ph), 6.66 (m, 1H, Ph), 6.28 (bs, 1H, NH), 6.16 (m, 1H, Ph), 5.61 (ddd, *J* = 17.2, 10.2, 8.6 Hz, 1H, 2-H), 5.03 (d, *J* = 10.2 Hz, 1-Ha), 4.99 (d, *J* = 3.1 Hz, 1H, 1-Hb), 3.58 (d, *J* = 8.9 Hz, 1H, 4-H), 3.29 (s, 3H, OMe), 3.21-3.19 (m, 1H, 5-H), 2.42 (dd, *J* = 13.3, 5.5 Hz, 1H, 8-Ha), 2.26-2.16 (m, 1H, 3-H), 2.09 (br. s, 1H, 4-OH), 2.09-2.03 (m, 1H, 8-Hb), 1.76-1.63 (m, 1H, 7-H), 1.57-1.51 (m, 1H, 6-Ha), 1.48 (s, 9H, *t*-Bu), 1.13 (d, *J* = 6.5 Hz, 3H, 3-Me), 1.08 (s, 9H, Si*t*-Bu), 1.07-1.02 (m, 1H, 6-Hb), 0.58 (d, *J* = 6.5 Hz, 3H, 7-Me) ppm; ^{13}C-NMR (100 MHz, $CDCl_3$, $CDCl_3$ = 77.16 ppm) δ 155.9 (s, C-Ar), 152.7 (s, NHCOO), 143.1 (s, C-Ar), 140.1 (d, C-2), 139.0 (s, C-Ar), 135.6 (d, C-Ar), 133.1 (s, C-Ar), 129.9 (d, C-Ar), 115.6 (t, C-1), 115.3 (d, C-Ar), 112.3 (d, C-Ar), 107.6 (d, C-Ar), 80.5 (s, *t*-Bu), 80.2 (d, C-5), 73.5 (d, C-4), 57.1 (q, OMe), 44.7 (t, C-8), 40.5 (d, C-3), 34.7 (t, C-6), 30.9 (d, C-7), 28.5 (q, *t*-Bu), 26.6 (q, Si*t*-Bu), 19.6 (s, Si*t*-Bu), 18.5 (q, 7-Me), 17.5 (q, 3-Me) ppm; HRMS (ESI) *m/z* for $C_{38}H_{53}NO_5SiNa$ $[M+Na]^+$: calculated: 654.3591, found: 654.3577; $[\alpha]^{20}_D$ = -17.8° (*c* = 1.0, $CHCl_3$).

3.3.2 Synthesis of eastern fragment 24

(*S*)-5-Oxotetrahydrofuran-2-carboxylic acid (22) (ref. S9)

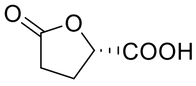

22

L-glutamic acid (19.0 g, 129 mmol, 1.0 eq.) was dissolved in water (140 mL), hydrochloric acid (2 M, 85 mL) was added, and the reaction mixture was cooled to -10 °C. $NaNO_2$ (10.7 g, 155 mmol, 1.2 eq.) was dissolved in water (85 mL) and slowly added to the reaction mixture within 3 h. The reaction mixture was cooled to room temperature and stirred for 12 h. The aqueous phase was extracted three times with ethyl acetate. The combined organic extracts were dried over $MgSO_4$ and concentrated under reduced pressure. The acid **22** (8.57 g, 65.9 mmol, 66%) was obtained as a colorless solid. ^{1}H-NMR (400 MHz, MeOD-d_4, MeOH = 3.31 ppm): δ 5.00 (dd, *J* = 7.8, 4.9 Hz, 1H, H-4), 2.70-2.55 (m, 2H, H-2), 2.55-2.40 (m, 1H, H-3a), 2.38-2.25 (m, 1H, H-3b) ppm; HRMS (ESI) *m/z* for $C_5H_5O_4$ $[M-H]^-$: calculated: 129.0188, found: 129.0190; mp= 69 °C; $[\alpha]^{20}_D$ = +14.5° (*c* = 1.0, MeOH); ref. S10: $[\alpha]^{20}_D$ = +15.6° (*c* = 2.0, EtOH).

(*S*)-5-(Hydroxymethyl)-2-oxotetrahydrofuran (S13)

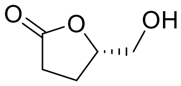

S13

Acid **22** (7.50 g, 57.7 mmol, 1.0 eq.) was dissolved in THF (200 mL) under argon atmosphere. The solution was cooled to 0 °C and BH_3*SMe_2 (8.20 mL, 86.5 mmol, 1.5 eq.) were added. The reaction mixture was warmed to room temperature stirred for 3 h. The reaction was terminated by addition of MeOH and the solvent was removed under reduced pressure to give lactone **S13** (6.5 g, 56.0 mmol, 97%) as a colorless oil. ^{1}H-NMR (400 MHz, MeOD-d_4, MeOH = 3.31 ppm): δ 4.72-4.56 (m, 1H, 4-H), 3.91 (dd, *J* = 1.9, 3.4 Hz,

1H, 5-Ha), 3.66 (dd, J = 11.9, 4.4 Hz, 1H, 5-Hb), 2.68-2.53 (m, 2H, 2-H), 2.35-2.05 (m, 2H, 3-H) ppm; $[\alpha]^{20}_{D}$ = +44.6° (c = 1.0, $CHCl_3$); ref. [S9]: $[\alpha]^{20}_{D}$ = +31.3° (c = 2.92, EtOH).

(*S*)-5-[(4-Methoxybenzyl)oxymethyl]-2-oxotetrahydrofuran (S14)

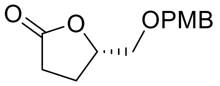

S14

Alcohol **S13** (1.66 g, 14.28 mmol, 1.0 eq.) was dissolved in CH_2Cl_2 (50 mL) at room temperature. 4-Methoxybenzyl-2,2,2-trichloroacetimidate (6.05 g, 21.42 mmol, 1.5 eq.) was added, followed by CSA (165.8 mg, 0.71 mmol, 5 mol%). Stirring was continued for 3 days before the reaction was terminated by the addition of a saturated aqueous $NaHCO_3$ solution. The phases were separated and the aqueous phase was extracted with CH_2Cl_2 (3 x 30 mL). The combined organic phases were dried over $MgSO_4$, filtered and the solvent was removed under reduced pressure. The crude product was purified by flash column chromatography (PE/EtOAc = 6:1 → 1:1) to furnish protected alcohol **S14** (3.09 g, 13.07 mmol, 92%) as an orange oil.

R*f* = 0.2 (PE/EtOAc = 2:1); ^{1}H-NMR (400 MHz, $CDCl_3$, $CHCl_3$ = 7.26 ppm): δ 7.24 (d, J = 8.8 Hz, 2H, PMB), 6.88 (app. d, J = 8.7 Hz, 2H, PMB), 4.68 – 4.62 (m, 1H, H-6), 4.49 (dd, J = 26.6, 3.4 Hz, 2H, PMB), 3.80 (s, 3H, PMB), 3.64 (dd, J = 10.7, 3.5 Hz, 1H, H-7), 3.55 (dd, J = 10.7, 4.2 Hz, 1H, H-7'), 2.61 (ddd, J = 17.7, 10.0, 6.6 Hz, 1H, H-4), 2.47 (ddd, J = 17.7, 9.9, 7.0 Hz, 1H, H-4'), 2.27 (dddd, J = 12.8, 9.9, 7.8, 6.6 Hz, 1H, H-5), 2.11 (dddd, J = 12.8, 10.0, 7.0, 5.9 Hz, 1H, H-5') ppm; $[\alpha]^{20}_{D}$ = +6.9 (c = 1.1, CH_2Cl_2; ref. [S10] $[\alpha]^{20}_{D}$ = +10.6, c = 1.0, $CHCl_3$; HRMS-ESI *m/z* for $C_{13}H_{16}O_4Na$ [M+Na]+ calc. 259.0947, found 259.0944.

(*S*)-5-[(4-Methoxybenzyl)oxy]pentane-1,4-diol (S15)

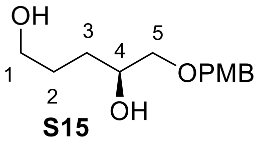

S15

PMB-ether **S14 (**1.72 g, 7.28 mmol, 1.0 eq.) as a solution in THF (20 mL) was slowly added to a suspension of lithium aluminium hydride (0.69 g, 18.21 mmol, 2.5 eq.) in THF (80 mL) at –10 °C. Stirring was continued for 2 h before the reaction was terminated by careful addition of MeOH (20 mL). A saturated aqueous Rochelle's salt solution and Et_2O was added and the mixture was allowed to reach room temperature and stirred vigorously for 5 h. The phases were separated and the aqueous phase was extracted with Et_2O (3 x 40 mL). The combined organic phases were washed with brine, dried over $MgSO_4$, filtered and the solvent was removed under reduced pressure. The corresponding diol **S15** (1.59 g, 6.63 mmol, 91%) was obtained as a colorless oil and was of sufficient purity to be used without further purification. R*f* = 0.3 (100% EtOAc); ^{1}H-NMR (400 MHz, $CDCl_3$, $CHCl_3$ = 7.26 ppm): δ 7.25 (app.1 d, J = 8.7 Hz, 2H, PMB), 6.88 (d, J = 8.7 Hz, 2H, PMB), 4.48 (s, 2H, PMB), 3.86 – 3.82 (m, 1H, H-6), 3.81 (s, 3H, PMB), 3.66 (ddd, J = 23.1, 10.8, 5.9 Hz, 2H, H-3), 3.47 (dd, J = 9.4, 3.3 Hz, 1H, H-7), 3.32 (dd, J = 9.4, 8.0 Hz, 1H, H-7'), 2.79 (bs, 1H, OH), 2.36 (bs, 1H, OH), 1.72 – 1.66 (m, 2H, H-4), 1.63 – 1.55 (m, 1H, H-5), 1.54 – 1.44 (m, 1H, H-5') ppm; $[\alpha]^{23}_{D}$ = –7.42 (c = 1.0, EtOH; **HRMS-ESI** *m/z* for $C_{13}H_{20}O_4Na$ [M+Na]+ calc. 263.1260, found 263.1258.

(*S*)-5-(*tert*-Butyldimethylsilyloxy)-1-[(4-methoxybenzyl)oxy]pentane-2-ol (S16)

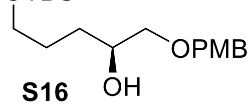

S16

Diol **S15** (0.85 g, 3.54 mmol, 1.0 eq.) was dissolved in CH_2Cl_2 (25 mL) under argon atmosphere, cooled to 0 °C, and mixed with imidazole (0.48 g, 7.08 mmol, 2.0 eq.) followed by TBSCl (0.59 g, 3.90 mmol, 1.1 eq.). The reaction mixture was heated to room temperature and stirred for 1 h. The reaction was terminated by addition of an aqueous NH_4Cl solution. The phases were separated and the aqueous phase was

extracted three times with CH_2Cl_2. The combined organic extracts were washed with brine, dried over $MgSO_4$ and concentrated under reduced pressure. The crude product was purified by flash chromatography (PE:EA= 5:1 → 2:1) to give the protected alcohol **S16** (1.25 g, 3.53 mmol, 99%) as a colorless oil; R_f = 0.7 (PE:EA= 1:1). ^{1}H-NMR (400 MHz, $CDCl_3$, $CHCl_3$ = 7.26 ppm) δ 7.30 (d, *J* = 8.4 Hz, 2H, PMB), 6.92 (d, *J* = 8.4 Hz, 2H, PMB), 4.53 (s, 2H, PMB), 3.90-3.81 (m, 1H, 4-H), 3.85 (s, 3H, PMB), 3.73-3.63 (m, 2H, 1-H), 3.51 (dd, *J* = 9.3, 3.7 Hz, 1H, 5-Ha), 3.38 (dd, *J* = 9.3, 7.4 Hz, 5-Hb), 2.86 (br. s, 1H, 4-OH), 1.75-1.50 (m, 2H, 2-H), 1.75-1.50 (m, 2H, 3-H), 0.93 (s, 9H, Si*t*-Bu), 0.09 (s, 6H, SiMe) ppm; ^{13}C-NMR (100 MHz, $CDCl_3$, $CDCl_3$ = 77.16 ppm) δ 159.6 (s, PMB), 129.7 (d, PMB), 114.2 (d, PMB), 74.6 (d, C-4), 73.3 (t, PMB), 70.6 (t, C-5), 63.6 (t, C-1), 55.6 (q, PMB), 30.6 (t, C-2), 29.3 (t, C-3), 26.3 (q, Si*t*-Bu), 18.5 (s, Si*t*-Bu), -5.00 (q, SiMe) ppm; HRMS (ESI) *m/z* for $C_{19}H_{35}O_4Si$ $[M+H]^+$: calculated: 355.2305, found: 355.2305; $[\alpha]^{20}_D$ = -2.3° (*c* = 1.0, $CHCl_3$). HRMS-ESI *m/z* for C20H36O4SiNa [M+Na]+ calc. 391.2281, found 391.2285.

(*S*)-5-(*tert*-Butyldimethylsilyloxy)-1-[(4-methoxybenzyl)oxy]-4-(methoxy)pentane (S17)

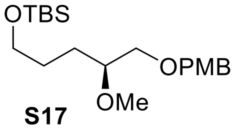

S17

Alcohol **S16** (1.20 g, 3.39 mmol, 1.0 eq.) was dissolved in Et_2O (20 mL) under argon atmosphere, cooled to 0 °C, and NaH (60% suspension in mineral oil) (0.20 g, 5.08 mmol, 1.5 eq.) was added followed by MeI (0.21 mL, 0.53 mmol, 1.1 eq.).
The reaction mixture was warmed up to room temperature and stirred for 72 h. The reaction was terminated by addition of water. The phases were separated and the aqueous phase was extracted three times with ethyl acetate. The combined organic extracts were washed with brine, dried over $MgSO_4$ and concentrated under reduced pressure. The crude product was purified by flash chromatography (PE:EA= 5:1 → 2:1) to give the protected alcohol **S17** (1.09 g, 2.96 mmol, 88%) as a colorless oil; R_f = 0.5 (PE:EA= 2:1). ^{1}H-NMR (400 MHz, $CDCl_3$, $CHCl_3$ = 7.26 ppm) δ 7.24 (d, *J* = 8.5 Hz, 2H, PMB), 6.87 (d, *J* = 8.5 Hz, 2H, PMB), 4.48 (s, 2H, PMB), 3.80 (s, 3H, PMB), 3.63-3.57 (m, 2H, 1-H), 3.45 (d, *J* = 4.8 Hz, 2H, 5-H), 3.04 (s, 3H, OMe), 3.38-3.34 (m, 1H, 4-H), 1.61-1.48 (m, 2H, 2-H), 1.61-1.48 (m, 2H, 3-H), 0.89 (s, 9H, Si*t*-Bu), 0.04 (s, 6H, SiMe) ppm; ^{13}C-NMR (100 MHz, $CDCl_3$, $CDCl_3$ = 77.16 ppm) δ 159.5 (s, PMB), 130.8 (s, PMB), 129.6 (d, PMB), 114.1 (d, PMB), 80.3 (d, C-4), 73.3 (t, PMB), 72.1 (t, C-5), 63.5 (t, C-1), 57.8 (q, OMe), 55.6 (q, PMB), 29.0 (t, C-2), 28.1 (t, C-3), 26.3 (q, Si*t*-Bu), 18.7 (s, Si*t*-Bu), -4.9 (q, SiMe) ppm; HRMS (ESI) *m/z* for $C_{20}H_{36}O_4SiNa$ $[M+Na]^+$: calculated: 391.2281, found: 391.2290; $[\alpha]^{20}_D$ = -6.0° (*c* = 1.0, $CHCl_3$)

(*S*)-5-(*tert*-Butyldimethylsilyloxy)-4-methoxypentane-1-ol (S18)

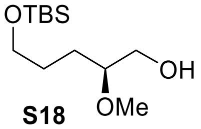

S18

Alcohol **S17** (1.05 g, 2.85 mmol, 1.0 eq.) was dissolved in CH_2Cl_2 (80 mL), cooled to 0 °C, and phosphate buffer (pH 7, 8 mL) and DDQ (0.78 g, 3.42 mmol, 1.2 eq.) were added.
The reaction mixture was warmed to room temperature and stirred for 1.5 h. The reaction was terminated by addition of an aqueous bicarbonate solution. The phases were separated and the aqueous phase was extracted three times with CH_2Cl_2. The combined organic extracts were washed with brine, dried over $MgSO_4$ and concentrated under reduced pressure. The crude product was purified by flash chromatography (PE:EA= 5:1 → 2:1) to afford the deprotected alcohol **S18** (0.65 g, 2.62 mmol, 92%) as a colorless oil; R_f = 0.4 (PE:EA= 2:1). ^{1}H-NMR (400 MHz, $CDCl_3$, $CHCl_3$ = 7.26 ppm) δ 3.71-3.65 (m, 1H, 5-Ha), 3.63-3.60 (m, 2H, 1-H), 3.52-3.47 (m, 1H, 5-Ha), 3.04 (s, 3H, OMe), 3.32-3.27 (m, 1H, 4-H), 1.98-1.95 (m, 1H, OH), 1.65-1.47 (m, 2H, 3-H), 1.65-1.47 (m, 2H, 2-H), 0.89 (s, 9H, Si*t*-Bu), 0.05 (s, 6H, SiMe) ppm; ^{13}C-NMR (100 MHz, $CDCl_3$, $CDCl_3$ = 77.16 ppm) δ 81.5 (d, C-4), 64.1 (t, C-5), 63.2 (t, C-1), 57.2 (q, OMe), 28.6 (t, C-2), 26.7 (t, C-3), 26.1 (q, Si*t*-Bu), 18.5 (s, Si*t*-Bu), -5.2 (q, SiMe) ppm; HRMS (ESI) *m/z* for $C_{12}H_{28}O_3SiNa$ $[M+Na]^+$: calculated: 271.1705, found: 271.1704; $[\alpha]^{20}_D$= +20.0° (*c* = 1.0, $CHCl_3$).

(*S*)-5-(*tert*-Butyldimethylsilyloxy)-4-methoxypentan-1-al (23)

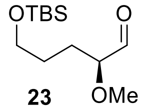

23

Alcohol **S30** (0.10 g, 0.40 mmol, 1.0 eq.) was dissolved in CH2Cl2 (30 mL), cooled to 0 °C, and $NaHCO_3$ (0.04 g, 0.48 mmol, 1.2 eq.) was added followed by the Dess-Martin periodinane reagent (0.21 g, 0.48 mmol, 1.2 eq.).

The reaction mixture was heated to room temperature and stirred for 1 h. The reaction was terminated by addition of an aqueous Na_2SO_3 solution. The phases were separated and the aqueous phase was extracted three times with CH_2Cl_2. The combined organic phases were washed with brine, dried over $MgSO_4$ and concentrated under reduced pressure. Aldehyde **S23** (0.10 g, 0.39 mmol, 99%) was obtained as a colorless foam; Rf = 0.7 (PE:EtOAc = 2:1). The aldehyde **23** was used directly without further characterization.

(3*S*,4*R*)-7-(*tert*-Butyldimethylsilyloxy)-4-methoxyhept-1-en-3-ol (24)

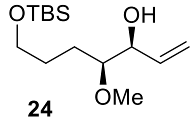

24

Aldehyde **23** (0.05 g, 0.20 mmol, 1.0 eq.) was dissolved in CH_2Cl_2 (3.0 mL) under an argon atmosphere, cooled to 0 °C, and $MgBr_2$*Et_2O (0.13 g, 0.49 mmol, 2.4 eq.) was added.

The reaction mixture was stirred at 0 °C for 30 min, then cooled to 78 °C and vinyl magnesium bromide (0.72 mL, 0.51 mmol, 2.5 eq.) (0.7 M solution in THF) was added slowly. After 3 h, the reaction was terminated by addition of an aqueous NH_4Cl solution. The phases were separated and the aqueous phase was extracted three times with CH_2Cl_2. The combined organic extracts were washed with brine solution, dried over $MgSO_4$ and concentrated under reduced pressure. The crude product was purified by flash chromatography (PE:EA= 5:1 → 2:1) to give alkene **24** (0.03 g, 0.10 mmol, 61%, from **S30**, *syn:anti* d.r. = 3:1) as a colorless oil; R_f = 0.5 (PE:EA= 2:1). ^{1}H-NMR (400 MHz, $CDCl_3$, $CHCl_3$ = 7.26 ppm) δ 5.89-5.80 (m, 1H, 6-H), 5.35 (d, *J* = 17.1 Hz, 1H, 7-Ha), 5.21 (d, *J* = 10.2 Hz, 1H, 7-Hb), 4.05-4.00 (m, 1H, 5-H), 3.63-3.57 (m, 2H, 1-H), 3.42 (s, 3H, OMe), 3.14-3.10 (m, 1H, 4-H), 2.55 (s, 1H, 5-OH), 1.70-1.48 (m, 2H, 3-H), 1.70-1.48 (m, 2H, 2-H), 0.89 (s, 9H, Si*t*-Bu), 0.04 (s, 6H, SiMe) ppm; ^{13}C-NMR (100 MHz, $CDCl_3$, $CDCl_3$ = 77.16 ppm) δ 137.5 (d, C-6), 117.2 (t, C-7), 83.9 (d, C-4), 74.5 (t, C-5), 63.2 (t, C-1), 58.1 (q, OMe), 28.2 (t, C-2), 26.2 (t, C-3), 26.1 (q, Si*t*-Bu), 18.5 (s, Si*t*-Bu), -5.2 (q, SiMe; HRMS (ESI) *m/z* for $C_{12}H_{28}O_3SiNa$ $[M+Na]^+$: calculated: 271.1705, found: 271.1704.
To verify the absolute configuration at C-5, the secondary alcohol was converted to the corresponding Mosher esters **S19a** and **S19b**,[S11] respectively, and analyzed by NMR spectroscopy.

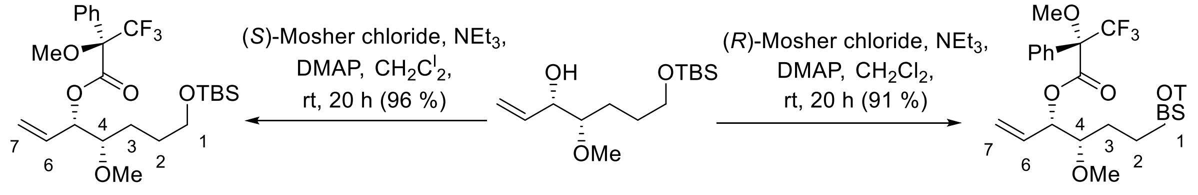

S19a **24** **S19b**

Table S3: Analysis of ^{1}H NMR specra of Mosher esters **S19a** and **S19b**.

signal	δ (*R*)-Mosher ester [ppm]	δ (*S*)-Mosher ester [ppm]	$\delta_S - \delta_R$
H-6	5.84	5.74	-0.10
H-7a	5.43	5.32	-0.11
H-7b	5.34	5.28	-0.06
H-4	3.27	3.34	0.07
OMe	3.24	3.38	0.14

3.3.3 End game synthesis towards seco acid derivative 28

Olefine metathesis product 25

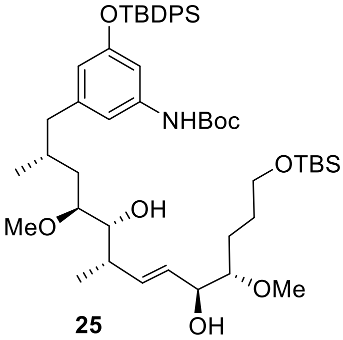

25

Alkene **19** (50.0 mg, 0.08 mmol, 1.0 eq.) was dissolved in $(CH_2Cl)_2$ (3.0 mL) under argon atmosphere, alkene **24** (40.0 mg, 0.13 mmol, 1.7 eq.) and Grubbs-Hoveyda II catalyst (4.90 mg, 8.00 µmol, 0.1 eq.) were added, and the reaction mixture was heated to 40 °C. After 10 h each, a further amount of Grubbs-Hoyveda II catalyst (4.90 mg, 8.00 µmol, 0.1 eq.) was added in portions. After 48 h, the reaction mixture was cooled to room temperature and the solvent was removed under reduced pressure.

The crude product was purified by flash chromatography (PE:EA= 10:1 → 2:1) followed by preparative HPLC (C18-P) (H_2O:MeOH = 80:20 {5 min}, gradient H_2O:MeOH = 80:20 → 0:100 {85 min}, H_2O:MeOH = 0:100 {10 min}, 15 mL/min) (t_r = 83. 0 min) was purified and alcohol **25** (27.0 mg, 30.7 µmol, 39%, 5*S*:5*R* d.r. = 6:1) was obtained as a colorless oil; R_f = 0.1 (PE:EA= 2:3). ^{1}H-NMR (400 MHz, $CDCl_3$, $CHCl_3$ = 7.26 ppm): δ 7.73-7.67 (m, 4H, SiPh), 7.42-7.33 (m, 6H, SiPh), 6.77 (s, 1H, Ph), 6.65 (s, 1H, Ph), 6.29 (s, 1H, NH), 6.16 (s, 1H, Ph), 5.60 (dd, *J* = 15.7, 8.3 Hz, 7-H), 5.48 (dd, *J* = 15.7, 5.6 Hz, 1H, 6-H), 4.01-3.95 (m, 1H, 5H), 3.63-3.55 (m, 1H, 9-H), 3.63-3.55 (m, 2H, 1-H), 3.41 (s, 3H, 4-OMe), 3.29 (s, 3H, 10-OMe), 3.23-3.15 (m, 1H, 4-H), 3.11-3.06 (m, 1H, 10-H), 2.54 (s, 1H, OH), 2.41 (dd, 1H, *J* = 13.4, 5.6 Hz, 13-Ha), 2.28-2.20 (m, 1H, 8H), 2.10-2.02 (m, 1H, 13-Hb), 1.80-1.71 (m, 1H, 12-H), 1.78-1.60 (m, 2H, 2-H), 1.78-1.60 (m, 2H, 3-H), 1.78-1.60 (m, 1H, 11-Ha), 1.48 (s, 9H, *t*-Bu), 1.12 (d, *J* = 6.6 Hz, 3H, 8-Me), 1.08 (s, 9H, Si*t*-Bu), 1.02-0.96 (m, 1H, 11-Hb), 0.89 (s, 9H, Si*t*-Bu), 0.59 (d, *J* = 6.5 Hz, 3H, 12-Me), 0.05 (s, 6H, SiMe) ppm; ^{13}C-NMR (100 MHz, $CDCl_3$, $CDCl_3$ = 77.16 ppm) δ 155.9 (s, C-Ar), 152.7 (s, NHCOO), 143.1 (s, C-Ar), 139.0 (d, C-Ar), 135.6 (s, C-Ar), 134.4 (d, C-7), 133.1 (s, C-Ar), 130.0 (s, C-Ar), 129.9 (d, C-6), 127.9 (s, C-Ar), 115.6 (d, C-Ar), 112.3 (d, C-Ar), 107.6 (d, C-Ar), 84.0 (d, C-4), 80.1 (d, C-10), 73.7 (d, C-9), 73.6 (d, C-5), 63.3 (t, C-1), 58.2 (q, 4-OMe), 57.2 (q, 10-OMe), 44.7 (t, C-13), 38.8 (d, C-8), 35.1 (t, C-11), 30.9 (d, C-12), 28.5 (t, C-2), 28.3 (q, *t*-Bu), 26.6 (q, Si*t*-Bu), 26.2 (q, Si*t*-Bu), 26.1 (t, C-3), 19.6 (s, Si*t*-Bu), 18.8 (q, 12-Me), 18.5 (s, Si*t*-Bu), 17.4 (q, 8-Me), -5.1 (q, SiMe) ppm; HRMS (ESI) *m/z* for $C_{50}H_{79}NO_8Si_2Na$ $[M+Na]^+$: calculated: 900.5242, found: 900.5239.

Diol S20

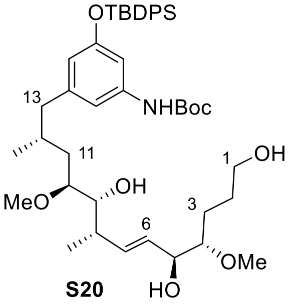

S20

Alcohol **25** (15.0 mg, 17.1 µmol, 1.0 eq.) was dissolved in CH_2Cl_2 (3.0 mL) under argon atmosphere, cooled to 0 °C, and added MeOH (2.0 mL) and CSA (2.00 mg, 5.10 µmol, 0.3 eq.). After 4 h, the reaction was terminated by addition of an aqueous bicarbonate solution. The phases were separated and the aqueous phase was extracted three times with CH_2Cl_2. The combined organic extracts were washed with brine, dried over $MgSO_4$ and concentrated under reduced pressure.

The crude product was purified by flash chromatography (PE:EA= 5:1 → 1:1) to afford alcohol **S20** (12.0 mg, 15.7 µmol, 92%) as a colorless oil; R_f = 0.1 (PE:EA= 2:3). ^{1}H-NMR (400 MHz, $CDCl_3$, $CHCl_3$ = 7.26 ppm): δ 7.73-7.67 (m, 4H, SiPh), 7.43-7.32 (m, 6H, SiPh), 6.82 (s, 1H, NH), 6.59 (s, 1H, Ph), 6.35 (s, 1H, Ph), 6.16 (s, 1H, Ph), 5.61 (dd, *J* = 15.8, 7.8 Hz, 1H, 7-H), 5.45 (dd, *J* = 15.8, 6.0 Hz, 6-H), 4.04-3.97 (m, 1H, 5-H), 3.68-3.61 (m, 2H, 1-H), 3.57 (dd, 1H, *J* = 7.9, 3.5 Hz, 9-H), 3.43 (s, 3H, 4-OMe), 3.29 (s, 3H, 10-OMe), 3.22-3.15 (m, 1H, 10-H), 3.13-3.08 (m, 1H, 4-H), 2.52 (s, 1H, OH), 2.43 (dd, *J* = 13.0, 5.8 Hz, 1H, 13-Ha), 2.30-2.20 (m, 1H, 8H), 2.07 (dd, 1H, *J* = 13.1, 8.8 Hz, 13-Hb), 1.80-1.58

(m, 1H, 12-H), 1.80-1.58 (m, 2H, 2-H), 1.80-1.58 (m, 2H, 3-H), 1.69-1.55 (m, 1H, 11-Ha), 1.47 (s, 9H, *t*-Bu), 1.30-1.20 (m, 1H, 11-Hb), 1.11 (d, *J* = 6.6 Hz, 3H, 8-Me), 1.08 (s, 9H, Si*t*-Bu), 0.60 (d, *J* = 6.4 Hz, 3H, 12-Me) ppm; ^{13}C-NMR (100 MHz, $CDCl_3$, $CDCl_3$ = 77.16 ppm) δ 155.9 (s, C-Ar), 152.7 (s, NHCOO), 143.1 (s, C-Ar), 139.0 (d, C-Ar), 135.7 (s, C-Ar), 135.6 (d, C-7), 133.1 (d, C-Ar), 129.9 (s, C-Ar), 129.6 (d, C-6), 129.0 (s, C-Ar), 127.9 (s, C-Ar), 115.7 (d, C-19), 112.3 (d, C-15), 107.6 (d, C-17), 84.1 (d, C-4), 80.2 (d, C-10), 79.8 (s, *t*-Bu), 73.9 (d, C-5), 73.8 (d, C-9), 62.9 (t, C-1), 58.3 (q, 4-OMe), 57.1 (q, 10-OMe), 44.6 (t, C-13), 39.0 (d, C-8), 35.0 (t, C-11), 31.0 (d, C-12), 28.5 (q, *t*-Bu), 28.0 (t, C-3), 26.6 (q, Si*t*-Bu), 26.5 (t, C-2), 19.6 (q, 12-Me), 18.8 (s, Si*t*-Bu), 17.3 (q, 8-Me) ppm; HRMS (ESI) *m/z* for $C_{44}H_{65}NO_8SiNa$ [M+Na]$^+$: calculated: 786.4377, found: 786.4372.

Per-*O*-silylated polyol S21

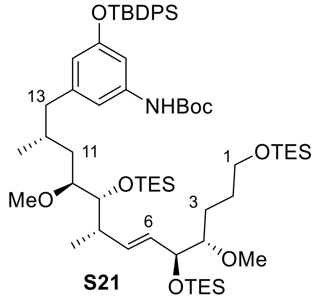

S21

Alcohol **S20** (30.0 mg, 39.0 µmol, 1.0 eq.) was dissolved in CH_2Cl_2 (15 mL) under argon atmosphere, cooled to 0 °C, and mixed with 2,6-lutidine (23.0 µL, 195 µmol, 5.0 eq.) followed by TESOTf (30.0 µL, 137 µmol, 3.5 eq.). The reaction mixture was warmed up to room temperature and stirred for an additional 3 h. The reaction was terminated by addition of an aqueous NH_4Cl solution. The phases were separated and the aqueous phase was extracted three times with CH_2Cl_2.

The combined organic extracts were washed with brine, dried over $MgSO_4$ and concentrated under reduced pressure. The crude product was purified by flash chromatography (PE:EA= 100:1 → 20:1) to afford the protected alcohol **S21** (35.0 mg, 31.6 µmol, 81%) as a colorless oil; R_f = 0.5 (PE:EA= 10:1). ^{1}H-NMR (400 MHz, C_6D_6, C_6D_5H = 7.16 ppm): δ 7.89-7.83 (m, 4H, SiPh), 7.27 (s, 1H, Ph), 7.23-7.19 (m, 6H, SiPh), 6.59 (s, 1H, Ph), 6.48 (s, 1H, Ph), 5.89 (s, 1H, NH), 5.66 (dd, 1H, *J* = 15.5, 6.3 Hz, 7-H), 5.56 (dd, 1H, *J* = 15.5, 8.6 Hz, 6-H), 4.38-4.34 (m, 1H, 5-H), 3.73-3.63 (m, 1H, 9-H), 3.73-3.63 (m, 2H, 1-H), 3.31 (s, 3H, 4-OMe), 3.35-3.30 (m, 1H, 10-H), 3.25 (s, 3H, 10-OMe), 3.22-3.17 (m, 1H, H-4), 2.46 (dd, *J* = 13.0, 5.7 Hz, 1H, 13-Ha), 2.36-2.27 (m, 1H, 8-H), 2.12 (dd, 1H, *J* =13.0, 9.0 Hz, 13-Hb), 2.05-1.95 (m, 1H, 12-H), 1.93-1.84 (m, 1H, 2-Ha), 1.93-1.84 (m, 1H, 11-Ha), 1.79-1.68 (m, 1H, 3-Ha), 1.79-1.68 (m, 1H, 2-Hb), 1.64-1.50 (m, 1H, 3-Hb), 1.39 (s, 9H, *t*-Bu), 1.25 (d, *J* = 6.7 Hz, 3H, 8-Me), 1.20 (s, 9H, Si*t*-Bu), 1.15-1.05 (m, 1H, 11-Hb), 1.12-1.03 (m, 27H, $SiCH_2CH_3$), 0.79-0.60 (m, 18H, $SiCH_2CH_3$), 0.79-0.60 (m, 3H, 12-Me) ppm; ^{13}C-NMR (100 MHz, C_6D_6, C_6D_6 = 128.06 ppm) δ 156.4 (s, C-Ar), 152.3 (s, NHCOO), 143.5 (s, C-Ar), 140.1 (d, C-Ar), 136.0 (s, C-Ar), 134.7 (d, C-7), 133.7 (d, C-Ar), 130.2 (d, C-6), 130.1 (s, C-Ar), 115.7 (d, C-Ar), 112.4 (d, C-Ar), 107.9 (d, C-Ar), 85.4 (d, C-4), 81.4 (d, C-10), 79.5 (s, *t*-Bu), 76.5 (d, C-9), 74.1 (d, C-5), 63.4 (t, C-1), 58.3 (q, 4-OMe), 57.2 (q, 10-OMe), 45.2 (t, C-13), 41.2 (d, C-8), 35.9 (t, C-11), 31.6 (d, C-12), 30.2 (t, C-2), 28.3 (q, *t*-Bu), 26.8 (q, Si*t*-Bu), 26.6 (t, C-3), 19.8 (s, Si*t*-Bu), 19.3 (q, 12-Me), 18.8 (q, 8-Me), 7.5 (q, $SiCH_2CH_3$), 7.3 (q, $SiCH_2CH_3$), 7.2 (q, $SiCH_2CH_3$), 5.7 (t, $SiCH_2CH_3$), 5.5 (t, $SiCH_2CH_3$), 4.9 (t, $SiCH_2CH_3$) ppm; HRMS (ESI): The recording of high-resolution mass spectrometry could not be successfully performed for this molecule.

Aldehyde S22

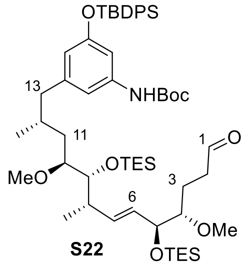

S22

DMSO (3.9 µL, 54 µmol, 3.0 eq.) was dissolved in CH_2Cl_2 (4.0 mL) under argon atmosphere, cooled to -78 °C, and oxalyl chloride (3.1 µL, 36 µmol, 2.0 eq.) was added. After 10 min, alcohol **S21**(20 mg, 18 µmol, 1.0 eq.) was dissolved in CH_2Cl_2 (0.6 mL) and added to the reaction mixture. After another 10 min, the reaction mixture was heated to 60 °C and stirred for 1.5 h. The reaction mixture was allowed to cool.

The reaction mixture was then cooled back to -78 °C, to which DIPEA (21 µL, 126 µmol, 7.0 eq.) was added and warmed up to room temperature. The reaction was terminated by addition of phosphate buffer (pH 7). The aqueous phase was extracted three times with CH_2Cl_2. The combined organic phases were washed brine, dried over $MgSO_4$ and concentrated under reduced pressure (91 % crude). The crude product was purified by flash chromatography (PE:EA= 50:1 → 10:1) to give aldehyde **S22** (13 mg, 13 µmol, 73%) as a colorless oil; R_f = 0.4 (PE:EA= 9:1). ^{1}H-NMR (400 MHz, C_6D_6, C_6D_5H = 7.16 ppm): δ 9.44 (s, 1H, 1-H), 7.90-7.81 (m, 4H, SiPh), 7.24 (s, 1H, Ph), 7.22-7.19 (m, 6H, SiPh), 6.65 (s, 1H, Ph), 6.48 (s, 1H, Ph), 5.94 (s, 1H, NH), 5.51-5.46 (m, 1H, 7-H), 5.51-5.46 (m, 1H, 6-H), 4.25-4.20 (m, 1H, 5-H), 3.67 (d, *J* = 9.2 Hz, 1H, 9-H), 3.29-3.25 (m, 1H, 10-H), 3.23 (s, 3H, 4-OMe), 3.20 (s, 3H, 10-OMe), 3.07-3.00 (m, 1H, H-4), 2.49 (dd, *J* = 13.2, 5.6 Hz, 1H, 13-Ha), 2.34-2.23 (m, 1H, 8-H), 2.21-2.06 (m, 2H, 2-H), 2.21-2.06 (m, 1H, H-13b), 2.06-1.93 (m, 1H, 12-H), 1.93-1.78 (m, 1H, 3-Ha), 1.93-1.78 (m, 1H, 11-Ha), 1.66-1.52 (m, 1H, 3-Hb), 1.39 (s, 9H, *t*-Bu), 1.22 (d, *J* = 6.7 Hz, 3H, 8-Me), 1.19-1.12 (m, 1H, 11-Hb), 1.19 (s, 9H, Si*t*-Bu), 1.12-1.04 (m, 18H, $SiCH_2CH_3$), 0.79-0.64 (m, 12H, $SiCH_2CH_3$), 0.79-0.71 (m, 3H, 12-Me) ppm; ^{13}C-NMR (100 MHz, C_6D_6, C_6D_6 = 128.06 ppm) δ 200.6 (t, C-1), 156.4 (s, C-Ar), 152.3 (s, NHCOO), 143.4 (s, C-Ar), 140.1 (d, C-Ar), 136.0 (s, C-Ar), 135.3 (d, C-7), 133.6 (d, C-Ar), 130.1 (s, C-Ar), 129.8 (d, C-6), 115.7 (d, C-Ar), 112.4 (d, C-Ar), 107.9 (d, C-Ar), 84.3 (d, C-4), 81.4 (d, C-10), 79.6 (s, *t*-Bu), 76.4 (d, C-9), 74.4 (d, C-5), 58.4 (q, 4-OMe), 57.2 (q, 10-OMe), 45.1 (t, C-13), 41.3 (d, C-8), 40.8 (t, C-2), 35.9 (t, C-11), 31.6 (d, C-12), 28.3 (q, *t*-Bu), 26.8 (q, Si*t*-Bu), 23.3 (t, C-3), 19.8 (s, Si*t*-Bu), 19.3 (q, 12-Me), 18.8 (q, 8-Me), 7.5 (q, $SiCH_2CH_3$), 7.2 (q, $SiCH_2CH_3$), 5.7 (t, $SiCH_2CH_3$), 5.4 (t, $SiCH_2CH_3$) ppm; HRMS (ESI): The recording of high-resolution mass spectrometry could not be successfully performed for this molecule.

Hemiacetal 26

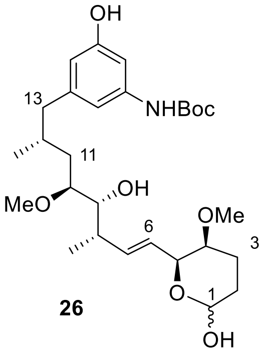

26

Aldehyde **S22** (21 mg, 21 µmol, 1.0 eq.) was dissolved in THF (8.0 mL) under argon atmosphere and pyridine (1.0 mL) and HF*Py (700 µL) were added. After 4 days, the reaction was terminated by addition of an aqueous bicarbonate solution. The phases were separated and the aqueous phase was extracted three times with ethyl acetate. The combined organic extracts were washed with brine, dried over $MgSO_4$ and concentrated under reduced pressure.The crude product was analyzed by preparative HPLC (C18 ISIS SP) (H_2O:MeOH = 90:10 {5 min}, gradient H_2O:MeOH = 90:10 → 0:100 {55 min}, H_2O:MeOH = 0:100 {10 min}, 2. 5 mL/min) (t_R = 54.6 min) was purified to give lactol **26** (10 mg, 19 µmol, 91%) as a colorless oil; R_f = 0.1 (PE:EA= 1:1). Compound **26** was used directly in the next steps without further purification. HRMS (ESI) *m/z* for $C_{28}H_{45}NO_8Na$ $[M+Na]^+$: calculated: 546.3043, found: 546.3045.

***N*-Boc SNAC-ester 27**

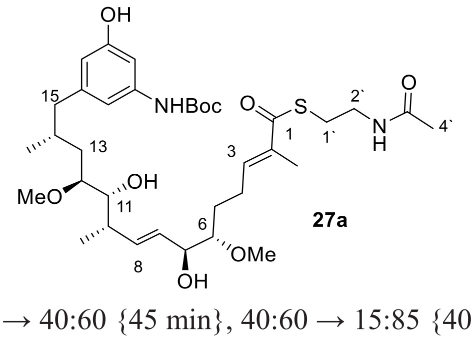

27a

Lactol **26** (5.0 mg, 9.6 µmol, 1.0 eq.) was dissolved in $CHCl_3$ (0.2 mL) to which phosphorylide $Ph_3P=C(CH_3)C(O)SCH_2CH_2NHAc$ (21 mg, 49 µmol, 5.0 eq.) was added and stirred at room temperature for 5 days. The solvent was removed under reduced pressure and the crude product was purified by flash chromatography (EtOAc). This was followed by preparative HPLC (C18 ISIS SP) (H_2O:MeOH = 70:30 {5 min}, gradient H_2O:MeOH = 70:30 → 40:60 {45 min}, 40:60 → 15:85 {40 min}, 15:85 → 0:100 {10 min}, 2.5 mL/min) (t_R = 50.0 min). The SNAC ester **27a** (3.5 mg, 5.1 µmol, 54%) was obtained as a colorless oil; R_f = 0.1 (EtOAc). ^{1}H-NMR (400 MHz, $CDCl_3$, $CHCl_3$ = 7.26 ppm): δ 6.83 (s, 1H, Ph), 6.72 (t, *J* = 7.4 Hz, 1H, 3-H), 6.64 (s,

1H, Ph), 6.49 (s, 1H, OH), 6.41 (s, 1H, NH), 6.34 (s, 1H, Ph), 5.92 (s, 1H, NH), 5.62 (dd, *J* = 15.5, 8.8 Hz, 1H, 9-H), 5.46 (dd, *J* = 15.5, 6.4 Hz, 8-H), 4.03-3.99 (m, 1H, 7-H), 3.65-3.60 (m, 1H, 11-H), 3.51-3.41 (m, 2H, 2'-H), 3.44 (s, 3H, 6-OMe), 3.33 (s, 3H, 12-OMe), 3.26-3.20 (m, 1H, 12-H), 3.14-3.09 (m, 1H, 6-H), 3.07-3.00 (m, 2H, 1'-H), 2.58 (dd, *J* = 13.3, 5.5 Hz, 1H, 15-Ha), 2.45 (d, *J* = 3.8 Hz, 1H, 7-OH), 2.35-2.25 (m, 1H, 10-H), 2.35-2.25 (m, 2H, 4-H), 2.35-2.25 (m, 1H, 15-Hb), 2.14 (d, *J* = 2.4 Hz, 1H, 11-OH), 1.98 (s, 3H, 4'-H), 1.97-1.90 (m, 1H, 14-H), 1.88 (s, 3H, 2-Me), 1.76-1.59 (m, 1H, 13-Ha), 1.76-1.59 (m, 1H, 5-Ha), 1.76-1.59 (m, 1H, 5-Hb), 1.50 (s, 9H, *t*-Bu), 1.28-1.17 (m, 1H, 13-Hb), 1.14 (d, *J* = 6.6 Hz, 3H, 10-Me), 0.81 (d, *J* = 6.5 Hz, 3H, 14-Me) ppm; ^{13}C-NMR (100 MHz, $CDCl_3$, $CDCl_3$ = 77.16 ppm) δ 194.5 (s, C-1), 170.9 (s, C-3'), 156.8 (s, C-Ar), 152.8 (s, NHCOO), 143.4 (s, C-Ar), 141.3 (d, C-3), 139.5 (s, C-Ar), 136.3 (d, C-2), 135.8 (d, C-9), 129.6 (d, C-8), 111.4 (d, C-Ar), 111.2 (d, C-Ar), 103.5 (d, C-Ar), 83.4 (d, C-6), 80.3 (d, C-12), 73.9 (d, C-7), 73.6 (d, C-11), 58.6 (q, 6-OMe), 57.2 (q, 12-OMe), 44.6 (t, C-15), 40.0 (t, C-2'), 39.1 (d, C-10), 34.8 (t, C-13), 31.0 (d, C-14), 28.9 (t, C-1'), 28.5 (q, *t*-Bu), 28.5 (t, C-5), 24.4 (t, C-4), 23.4 (q, C-4'), 19.3 (q, 14-Me), 17.8 (q, 10-Me), 12.7 (q, 2-Me) ppm; HRMS (ESI) *m/z* for $C_{35}H_{56}N_2O_9SNa$ [M+Na]$^+$: calculated: 703.3604, found: 703.3610.

SNAC-ester 27b

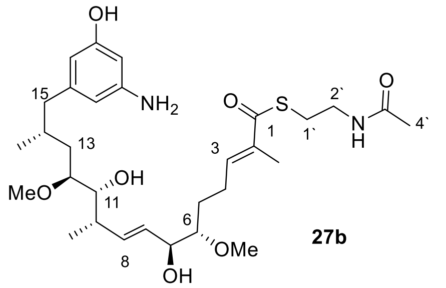

27b

The SNAC ester **27a** (3.0 mg, 4.4 µmol, 1.0 eq.) was dissolved in CH_2Cl_2 (3.0 mL) under argon atmosphere, cooled to 0 °C, and TFA (350.0 µl) was added. After 3 h, the reaction was terminated by addition of an aqueous bicarbonate solution. The layers were separated and the aqueous phase was extracted three times with ethyl acetate. The combined organic phases were washed with brine, dried over $MgSO_4$ and concentrated under reduced pressure. The crude product was purified by preparative HPLC (C18 ISIS SP) (H_2O:MeOH = 70:30 {5 min}, gradient H_2O:MeOH = 70:30 → 40:60 {80 min}, 40:60 → 15:85 {5 min}, 15:85 → 0:100 {10 min}, 2.5 mL/min) (t_R = 55.6 min) to give SNAC ester **27b** (2.0 mg, 3.4 µmol, 77%) as a colorless oil. ^{1}H-NMR (400 MHz, $CDCl_3$, $CHCl_3$ = 7.26 ppm): δ 6.75-6.70 (m, 1H, 3-H), 6.23 (s, 1H, OH), 6.07 (s, 1H, Ph), 6.07 (s, 1H, Ph), 6.04 (s, 1H, Ph), 5.89 (s, 1H, NH), 5.62 (dd, *J* = 15.7, 8.2 Hz, 1H, 9-H), 5.48 (dd, *J* = 15.7, 6.5 Hz, 8-H), 4.05-4.00 (m, 1H, 7-H), 3.66-3.61 (m, 1H, 11-H), 3.49-3.46 (m, 2H, 2'-H), 3.44 (s, 3H, 6-OMe), 3.34 (s, 3H, 12-OMe), 3.26-3.21 (m, 1H, 12-H), 3.15-3.09 (m, 1H, 6-H), 3.09-3.06 (m, 2H, 1'-H), 2.53 (dd, *J* = 13.2, 5.8 Hz, 1H, 15-Ha), 2.45 (d, *J* = 3.8 Hz, 1H, 7-OH), 2.35-2.26 (m, 1H, 10-H), 2.35-2.26 (m, 2H, 4-H), 2.19 (dd, *J* = 13.2, 8.2 Hz, 1H, 15-Hb), 2.12 (d, *J* = 2.4 Hz, 1H, 11-OH), 1.97 (s, 3H, 4'-H), 1.98-1.88 (m, 1H, 14-H), 1.89 (s, 3H, 2-Me), 1.75-1.58 (m, 1H, 13-Ha), 1.73-1.62 (m, 2H, 5-H), 1.26-1.17 (m, 1H, 13-Hb), 1.14 (d, *J* = 6.6 Hz, 3H, 10-Me), 0.82 (d, *J* = 6.4 Hz, 3H, 14-Me) ppm; ^{13}C-NMR (100 MHz, $CDCl_3$, $CDCl_3$ = 77.16 ppm) δ 194.4 (s, C-1), 170.9 (s, C-3'), 157.2 (s, C-Ar), 147.7 (s, C-Ar), 143.8 (d, C-3), 141.3 (s, C-Ar), 136.4 (s, C-2), 135.8 (d, C-9), 129.6 (d, C-8), 108.7 (d, C-Ar), 107.1 (d, C-Ar), 100.2 (d, C-Ar), 83.4 (d, C-6), 80.4 (d, C-12), 73.9 (d, C-7), 73.6 (d, C-11), 58.6 (q, 6-OMe), 57.2 (q, 12-OMe), 44.7 (t, C-15), 40.0 (t, C-2'), 39.0 (d, C-10), 34.9 (t, C-13), 30.9 (d, C-14), 28.9 (t, C-1'), 28.5 (t, C-5), 24.4 (t, C-4), 23.4 (q, C-4'), 19.4 (q, 14-Me), 17.7 (q, 10-Me), 12.7 (q, 2-Me) ppm; HRMS (ESI) *m/z* for $C_{30}H_{49}N_2O_7S$ [M+H]$^+$: calculated: 581.3260, found: 581.3262; $[\alpha]^{20}_D$ = -9.0° (*c* = 0.6, MeOH).

3.3.4 End game synthesis towards seco acid derivative 29

TBS-ether S23

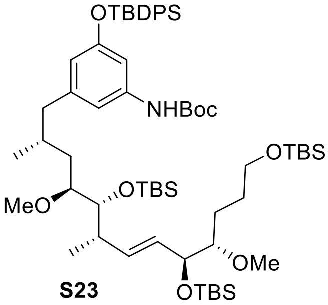

S23

Diol **S23** (24.0 mg, 27.3 µmol, 1.0 eq.) was dissolved in CH_2Cl_2 (10 mL) under argon atmosphere, cooled to -78 °C, and 2,6-lutidine (15.6 µL, 137 µmol, 5.0 eq.) was added followed by TBSOTf (19.0 µL, 82.0 µmol, 3.0 eq.). The reaction mixture was heated to room temperature and stirring was continued for an additional 3 h at room temperature. The reaction was terminated by addition of an aqueous NH_4Cl solution. The phases were separated and the aqueous phase was extracted three times with CH_2Cl_2. The combined organic phases were washed with brine, dried over $MgSO_4$, filtered and concentrated under reduced pressure. The crude product was purified by flash chromatography (PE:EtOAc = 100:1 → 20:1) to afford the protected alcohol **S23** (28.0 mg, 25.3 µmol, 93%, d.r.= 4:1) as a colorless oil; Rf = 0.5 (PE:EtOAc = 10:1).

^{1}H-NMR (400 MHz, $CDCl_3$, $CHCl_3$ = 7.26 ppm): δ 7.74-7.68 (m, 4H, SiPh), 7.42-7.31 (m, 6H, SiPh), 6.71 (s, 1H, Ph), 6.68 (s, 1H, Ph), 6.24 (s, 1H, NH), 6.17 (s, 1H, Ph), 5.47-5.37 (m, 1H, 7-H), 5.47-5.37 (m, 1H, 6-H), 4.10-4.03 (m, 1H, 5-H), 3.65-3.47 (m, 1H, 9-H), 3.65-3.47 (m, 2H, 1-H), 3.38 (s, 3H, 4-OMe), 3.21 (s, 3H, 10-OMe), 3.15-3.09 (m, 1H, 10-H), 3.09-3.00 (m, 1H, H-4), 2.40 (dd, *J* = 13.2, 5.8 Hz, 1H, 13-Ha), 2.23-2.12 (m, 1H, 8-H), 2.05-2.00 (m, 1H, 13-Hb), 1.76-1.60 (m, 1H, 12-H), 1.76-1.60 (m, 1H, 2-Ha), 1.58-1.53 (m, 1H, 11-Ha), 1.56-1.49 (m, 1H, 2-Hb), 1.48-1.36 (m, 1H, 3-Ha), 1.25-1.17 (m, 1H, 3-Hb), 1.47 (s, 9H, *t*-Bu), 1.07-1.03 (m, 1H, 11-Hb), 1.07 (s, 9H, Si*t*-Bu), 1.03 (d, *J* = 6.7 Hz, 3H, 8-Me), 0.90 (s, 9H, Si*t*-Bu), 0.89 (s, 9H, Si*t*-Bu), 0.86 (s, 9H, Si*t*-Bu), 0.58 (d, *J* = 6.4 Hz, 3H, 12-Me), 0.07 (s, 3H, SiMe), 0.06 (s, 9H, SiMe), 0.04 (s, 6H, SiMe) ppm; ^{13}C-NMR (100 MHz, $CDCl_3$, $CDCl_3$ = 77.16 ppm) δ 155.9 (s, C-Ar), 152.6 (s, NHCOO), 143.4 (s, C-Ar), 138.9 (s, C-Ar), 135.6 (d, C-Ar), 134.6 (d, C-7), 133.2 (s, C-Ar), 130.5 (d, C-6), 129.8 (s, C-Ar), 127.8 (d, C-Ar), 115.6 (d, C-Ar), 112.3 (d, C-Ar), 107.5 (d, C-Ar), 85.3 (d, C-4), 81.3 (d, C-10), 80.3 (s, *t*-Bu), 75.3 (d, C-9), 73.7 (d, C-5), 63.5 (t, C-1), 58.5 (q, 4-OMe), 57.1 (q, 10-OMe), 44.7 (t, C-13), 40.7 (d, C-8), 36.0 (t, C-11), 31.2 (d, C-12), 29.2 (t, C-2), 28.5 (q, *t*-Bu), 26.7 (q, Si*t*-Bu), 26.3 (q, Si*t*-Bu), 26.1 (q, Si*t*-Bu), 25.8 (t, C-3), 19.6 (q, 12-Me), 19.0 (s, Si*t*-Bu), 18.6 (q, 8-Me), 18.3 (s, Si*t*-Bu), -3.8 (q, SiMe), -4.0 (q, SiMe), -4.6 (q, SiMe), -5.1 (q, SiMe) ppm; HRMS (ESI): The recording of high-resolution mass spectrometry could not be successfully performed for this molecule.

Alcohol S24

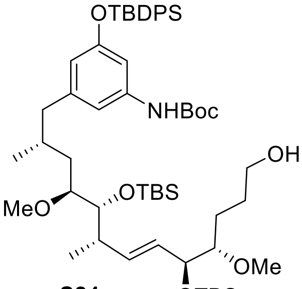

S24

Silyl ether **S23** (10 mg, 9.0 µmol, 1.0 eq.) was dissolved in CH_2Cl_2 (2.0 mL) under argon atmosphere, cooled to 0 °C, and added MeOH (1.3 mL) and CSA (0.6 mg, 2.7 µmol, 0.3 eq.). After 4 h, the reaction was terminated by addition of an aqueous $NaHCO_3$ solution. The aqueous phase was extracted three times with CH_2Cl_2. The combined organic phases were washed with aqueous NaCl solution, dried over $MgSO_4$ and concentrated under reduced pressure. The crude product was purified by flash chromatography (PE:EtOAc = 10:1 → 5:1) to give alcohol **S24** (8.0 mg, 8.1 µmol, 88%, d.r. = 4:1) as a colorless oil; Rf = 0.1 (PE:EtOAc = 9:1).

^{1}H-NMR (400 MHz, $CDCl_3$, $CHCl_3$ = 7.26 ppm): δ 7.74-7.67 (m, 4H, SiPh), 7.45-7.30 (m, 6H, SiPh), 6.73 (s, 1H, Ph), 6.65 (s, 1H, Ph), 6.30 (s, 1H, NH), 6.17 (s, 1H, Ph), 5.51-5.35 (m, 1H, 7-H), 5.51-5.35 (m, 1H, 6-H), 4.17-4.08 (m, 1H, 5-H), 3.67-3.57 (m, 2H, 1-H), 3.52 (dd, *J* = 8.7, 1.3 Hz, 1H, 9-H), 3.40 (s, 3H, 4-OMe), 3.21 (s, 3H, 10-OMe), 3.19-3.10 (m, 1H, 10-H), 3.10-3.03 (m, 1H, H-4), 2.42 (dd, *J* = 13.3, 5.6 Hz, 1H, 13-Ha), 2.23-2.15 (m, 1H, 8-H), 2.09-2.02 (m, 1H, 13-Hb), 1.74-1.60 (m, 1H, 12-H), 1.74-1.60 (m, 2H, 2-H), 1.60-1.46 (m, 2H, 3-H), 1.60-1.46 (m, 1H, 11-Ha), 1.47 (s, 9H, *t*-Bu), 1.07 (s, 9H, Si*t*-Bu), 1.03 (d, *J* = 6.7 Hz, 3H, 8-Me), 1.03-0.89 (m, 1H, 11-Hb), 0.89 (s, 9H, Si*t*-Bu), 0.86 (s, 9H,

Si*t*-Bu), 0.58 (d, *J* = 6.5 Hz, 3H, 12-Me), 0.06 (s, 3H, SiMe), 0.03 (s, 6H, SiMe), 0.02 (s, 3H, SiMe) ppm; ^{13}C-NMR (100 MHz, $CDCl_3$, $CDCl_3$ = 77.16 ppm) δ 155.9 (s, C-Ar), 152.7 (s, NHCOO), 143.4 (s, C-Ar), 138.9 (s, C-Ar), 135.7 (d, C-Ar), 134.8 (d, C-7), 133.2 (s, C-Ar), 130.3 (d, C-6), 129.8 (s, C-Ar), 127.8 (d, C-Ar), 115.6 (d, C-Ar), 112.3 (d, C-Ar), 107.5 (d, C-Ar), 85.5 (d, C-4), 81.4 (d, C-10), 80.3 (s, *t*-Bu), 75.9 (d, C-9), 74.9 (d, C-5), 63.1 (t, C-1), 58.4 (q, 4-OMe), 57.1 (q, 10-OMe), 44.7 (t, C-13), 40.9 (d, C-8), 36.1 (t, C-11), 31.3 (d, C-12), 29.4 (t, C-2), 28.5 (q, *t*-Bu), 26.8 (t, C-3), 26.7 (q, Si*t*-Bu), 26.3 (q, Si*t*-Bu), 26.0 (q, Si*t*-Bu), 19.6 (s, Si*t*-Bu), 19.0 (q, 12-Me), 18.6 (s, Si*t*-Bu), 18.3 (q, 8-Me), -3.8 (q, SiMe), -4.0 (q, SiMe), -4.6 (q, SiMe) ppm; HRMS (ESI): The recording of high-resolution mass spectrometry could not be successfully performed for this molecule.

Aldehyde S25

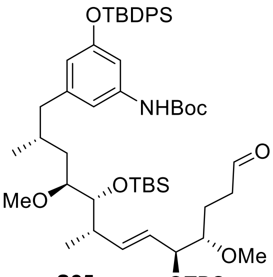

S25

Alcohol **S24** (30.0 mg, 30.2 µmol, 1.0 eq.) was dissolved in CH_2Cl_2 (8.0 mL), cooled to 0 °C, and $NaHCO_3$ (3.00 mg, 36.3 µmol, 1.2 eq.) was added followed by the Dess-Martin reagent (15.4 mg, 36.3 µmol, 1.2 eq.). The reaction mixture was warmed to room temperature and stirred for 1 h. The reaction was terminated by addition of an aqueous Na_2SO_3 solution. The phases were separated and the aqueous phase was extracted three times with CH_2Cl_2. The combined organic phases were washed with brine, dried over $MgSO_4$ and concentrated under reduced pressure. The crude product was purified by flash chromatography (PE:EtOAc = 50:1 → 10:1) to give aldehyde **S25** (21.0 mg, 21.2 µmol, 70%, d.r. = 4:1) as a colorless oil; Rf = 0.4 (PE:EtOAc = 9:1).

^{1}H-NMR (400 MHz, C_6D_6, C_6D_5H = 7.16 ppm): δ 9.44 (s, 1H, 1-H), 7.86-7.84 (m, 4H, SiPh), 7.21-7.17 (m, 6H, SiPh), 7.19 (s, 1H, 17-H), 6.65 (s, 1H, 15-H), 6.47 (s, 1H, 19-H), 5.97 (s, 1H, NH), 5.64 (dd, *J* = 15.5, 8.5 Hz, 1H, 7-H), 5.51 (dd, *J* = 15.5, 5.3 Hz, 1H, 6-H), 4.25-4.20 (m, 1H, 5-H), 3.67 (d, *J* = 8.5 Hz, 1H, 9-H), 3.31-3.25 (m, 1H, 10-H), 3.21 (s, 3H, 4-OMe), 3.18 (s, 3H, 10-OMe), 3.03-2.99 (m, 1H, H-4), 2.48 (dd, *J* = 12.6, 5.5 Hz, 1H, 13-Ha), 2.35-2.24 (m, 1H, 8-H), 2.18-2.08 (m, 2H, 2-H), 2.18-2.08 (m, 1H, H-13b), 2.01-1.92 (m, 1H, 12-H), 1.89-1.75 (m, 1H, 11-Ha), 1.89-1.75 (m, 1H, 3-Ha), 1.65-1.54 (m, 1H, 3-Hb), 1.39 (s, 9H, *t*-Bu), 1.19 (s, 9H, Si*t*-Bu), 1.16 (d, *J* = 6.8 Hz, 3H, 8-Me), 1.19-1.16 (m, 1H, 11-Hb), 1.07 (s, 9H, Si*t*-Bu), 1.01 (s, 9H, Si*t*-Bu), 0.75 (d, *J* = 6.5 Hz, 3H, 12-Me), 0.21 (s, 3H, SiMe), 0.15 (s, 6H, SiMe), 0.12 (s, 3H, SiMe) ppm; ^{13}C-NMR (100 MHz, C_6D_6, C_6D_6 = 128.06 ppm) δ 200.6 (d, C-1), 156.5 (s, C-Ar), 152.4 (s, NHCOO), 143.5 (s, C-Ar), 140.2 (d, C-Ar), 136.1 (s, C-Ar), 135.2 (d, C-7), 133.7 (d, C-Ar), 130.2 (s, C-Ar), 129.5 (d, C-6), 115.8 (d, C-19), 112.6 (d, C-15), 108.0 (d, C-17), 84.6 (d, C-4), 81.6 (d, C-10), 79.7 (s, *t*-Bu), 76.8 (d, C-9), 73.9 (d, C-5), 58.5 (q, 4-OMe), 57.1 (q, 10-OMe), 45.1 (t, C-13), 40.9 (d, C-8), 40.7 (t, C-2), 36.4 (t, C-11), 31.8 (d, C-12), 28.4 (q, *t*-Bu), 26.9 (q, Si*t*-Bu), 26.6 (q, Si*t*-Bu), 26.3 (q, Si*t*-Bu), 23.3 (t, C-3), 19.9 (q, 12-Me), 19.5 (s, Si*t*-Bu), 19.0 (q, 8-Me), 18.6 (s, Si*t*-Bu), -3.4 (q, SiMe), -4.1 (q, SiMe), -4.5 (q, SiMe) ppm; HRMS (ESI). The recording of high-resolution mass spectrometry could not be successfully performed for this molecule.

Ethyl thioester 29

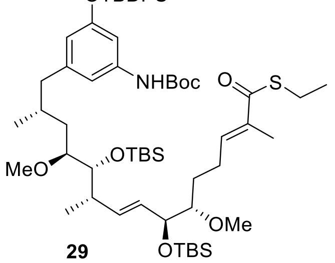

29

Aldehyde **S25** (14.0 mg, 14.1 µmol, 1.0 eq.) was dissolved in $CHCl_3$ (2.5 mL) and phosphorylide $Ph_3P{=}C(CH_3)C(O)SEt$ **28b** (6.20 mg, 16.9 µmol, 1.2 eq.) was added. The reaction mixture was heated to 40 °C and stirred for an additional 24 h at 40 °C. The solvent was removed under reduced pressure, the crude product was purified by flash chromatography (PE:EtOAc = 20:1 → 10:1), and thioester **29** (13.0 mg, 12.1 µmol, 86%, d.r. = 4:1) was obtained as a colorless oil; R_f = 0.4 (PE:EtOAc = 10:1).

^{1}H-NMR (400 MHz, $CDCl_3$, $CHCl_3$ = 7.26 ppm): δ 7.77-7.66 (m, 4H, SiPh), 7.45-7.29 (m, 6H, SiPh), 6.87 (ddd, *J* = 15.5, 9.3, 4.8 Hz, 1H, 3-H), 6.72 (s, 1H, Ph), 6.68 (s, 1H, Ph), 6.26 (s, 1H, NH), 6.16 (s, 1H, Ph), 5.54-5.31 (m, 1H, 9-H), 5.54-5.31 (m, 1H, 8-H), 4.12-4.08 (m, 1H, 7-H), 3.52 (dd, *J* = 8.6, 1.3 Hz, 1H, 11-H), 3.37 (s, 3H, 6-OMe), 3.21 (s, 3H, 12-OMe), 3.16-3.10 (m, 1H, 12-H), 3.05-2.97 (m, 1H, H-6), 2.97-2.87 (m, 2H, SCH_2CH_3), 2.39 (dd, *J* = 13.3, 5.7 Hz, 1H, 15-Ha), 2.35-2.26 (m, 1H, 4-Ha), 2.26-2.13 (m, 1H, 10-H), 2.26-2.13 (m, 1H, 4-Hb), 2.13-1.97 (m, 1H, H-15b), 1.80 (s, 3H, 2-Me), 1.77-1.60 (m, 1H, 14-H), 1.64-1.43 (m, 2H, 5-Ha), 1.63-1.50 (m, 1H, 13-Ha), 1.47 (s, 9H, *t*-Bu), 1.28-1.25 (m, 3H, SCH_2CH_3), 1.08 (s, 9H, Si*t*-Bu), 1.04 (d, *J* = 6.7 Hz, 3H, 10-Me), 1.05-1.00 (m, 1H, 13-Hb), 0.89 (s, 9H, Si*t*-Bu), 0.86 (s, 9H, Si*t*-Bu), 0.58 (d, *J* = 6.4 Hz, 3H, 14-Me), 0.05 (s, 3H, SiMe), 0.03 (s, 6H, SiMe), 0.02 (s, 3H, SiMe) ppm; ^{13}C-NMR (100 MHz, $CDCl_3$, $CDCl_3$ = 77.16 ppm) δ 190.3 (d, C-1), 155.9 (s, C-Ar), 152.6 (s, NHCOO), 145.3 (d, C-3), 143.3 (s, C-Ar), 139.0 (s, C-Ar), 135.9 (s, C-2), 135.7 (d, C-Ar), 135.1 (d, C-9), 133.2 (s, C-Ar), 130.2 (d, C-8), 129.9 (d, C-Ar), 127.8 (d, C-Ar), 115.6 (d, C-Ar), 112.3 (d, C-Ar), 107.6 (d, C-Ar), 84.4 (d, C-6), 81.3 (d, C-12), 80.3 (s, *t*-Bu), 75.9 (d, C-11), 74.7 (d, C-7), 58.5 (q, 6-OMe), 57.2 (q, 12-OMe), 44.7 (t, C-15), 40.8 (d, C-10), 36.0 (t, C-13), 31.2 (d, C-14), 28.6 (t, C-4), 28.6 (t, C-5), 28.5 (q, *t*-Bu), 26.7 (q, Si*t*-Bu), 26.3 (q, Si*t*-Bu), 26.0 (q, Si*t*-Bu), 23.2 (t, SCH_2CH_3), 19.6 (s, Si*t*-Bu), 19.0 (q, 14-Me), 18.6 (s, Si*t*-Bu), 18.3 (q, 10-Me), 15.0 (q, SCH_2CH_3), 12.4 (q, 2-Me), -3.8 (q, SiMe), -4.0 (q, SiMe), -4.6 (q, SiMe) ppm.

Ethyl thioester S26

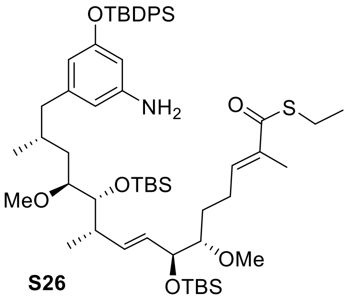

S26

Thioester **29** (11.0 mg, 10.2 µmol, 1.0 eq.) was dissolved in CH_2Cl_2 (6.0 mL) under argon atmosphere, cooled to 0 °C, and TFA (400 µL) was added. After 4 h, the reaction was terminated by addition of an aqueous $NaHCO_3$ solution. The phases were separated and the aqueous phase was extracted three times with CH_2Cl_2. The combined organic phases were washed with aqueous NaCl solution, dried over $MgSO_4$, filtered and concentrated under reduced pressure. The crude product was purified by flash chromatography (PE:EtOAc = 10:1 → 3:1) to afford amine **S26** (4.00 mg, 4.10 µmol, 40%, d.r. = 4:1) as a colorless oil; R_f = 0.2 (PE:EtOAc = 9:1).

^{1}H-NMR (400 MHz, $CDCl_3$, $CHCl_3$ = 7.26 ppm): δ 7.79-7.66 (m, 4H, SiPh), 7.45-7.29 (m, 6H, SiPh), 6.95-6.79 (m, 1H, 3-H), 6.05 (s, 1H, Ph), 6.01 (s, 1H, Ph), 5.94 (s, 1H, Ph), 5.54-5.34 (m, 1H, 9-H), 5.54-5.34 (m, 1H, 8-H), 4.19-4.02 (m, 1H, 7-H), 3.59-3.53 (m, 1H, 11-H), 3.37 (s, 3H, 6-OMe), 3.23 (s, 3H, 12-OMe), 3.20-3.10 (m, 1H, 12-H), 3.05-2.98 (m, 1H, H-6), 2.98-2.93 (m, 2H, SCH_2CH_3), 2.40 (dd, *J* = 13.3, 5.7 Hz, 1H, 15-Ha), 2.35-2.26 (m, 1H, 4-Ha), 2.26-2.13 (m, 1H, 10-H), 2.26-2.13 (m, 1H, 4-Hb), 2.07-1.97 (m, 1H, H-15b), 1.82 (s, 3H, 2-Me), 1.80-1.70 (m, 1H, 14-H), 1.65-1.43 (m, 2H, 5-Ha), 1.65-1.43 (m, 1H, 13-Ha), 1.31-1.22 (m, 3H, SCH_2CH_3), 1.07 (s, 9H, Si*t*-Bu), 1.04 (d, *J* = 6.8 Hz, 3H, 10-Me), 1.05-1.00 (m, 1H, 13-Hb), 0.89 (s, 9H, Si*t*-Bu), 0.88 (s, 9H, Si*t*-Bu), 0.62 (d, *J* = 6.5 Hz, 3H, 14-Me), 0.07 (s, 3H, SiMe), 0.05 (s, 6H, SiMe), 0.02 (s, 3H, SiMe) ppm.

3.3.5 Macrolactamizations

Progeldanamycin derivate S27 [S12]

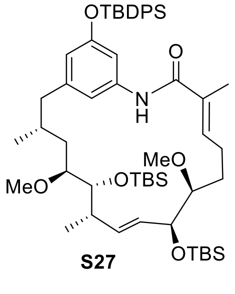

S27

Thioester **S26** (4.0 mg, 4.1 µmol, 1.0 eq.) was dissolved in THF (6.0 mL) under an argon atmosphere and 2,6-lutidine (40 µL) and silver nitrate (11 mg, 62 µmol, 15 eq.) were added. The reaction mixture was stirred at 55 °C for 1.5d and then cooled to room temperature. The reaction was terminated by addition of an aqueous $CuSO_4$ solutionThe phases were separated and the aqueous layer was extracted three times with EtOAc. The combined organic phases were washed with brine, dried over $MgSO_4$ and concentrated under reduced pressure. The crude product was purified by flash chromatography

(PE:EtOAc= 10:1→3:1) to afford progeldanamycin derivative **S27** (2.0 mg, 2.2 µmol, 53%, d.r. = 4:1) as a colorless oil; R_f = 0.2 (PE:EtOAc = 9:1).
^{1}H-NMR (400 MHz, $CDCl_3$, $CHCl_3$ = 7.26 ppm): δ 7.77-7.64 (m, 4H, SiPh), 7.47-7.34 (m, 6H, SiPh), 6.97-6.84 (m, 1H, 3-H), 6.47 (s, 1H, Ph), 6.40 (s, 1H, Ph), 6.28 (s, 1H, Ph), 5.43 (dd, *J* = 15.3, 8.5 Hz, 1H, 8-H), 5.30 (dd, *J* = 15.3, 5.9 Hz, 1H, 9-H), 4.37-4.30 (m, 1H, 7-H), 3.58-3.53 (m, 1H, 11-H), 3.42 (s, 3H, 6-OMe), 3.28 (s, 3H, 12-OMe), 3.20-3.12 (m, 1H, 12-H), 3.12-3.07 (m, 1H, H-6), 2.91-2.81 (m, 1H, 15-Ha), 2.28-2.15 (m, 1H, 10-H), 2.15-2.00 (m, 2H, 4-H), 2.15-2.00 (m, 1H, H-15b), 1.77 (s, 3H, 2-Me), 1.76-1.70 (m, 1H, 14-H), 1.65-1.43 (m, 2H, 5-H), 1.65-1.43 (m, 1H, 13-Ha), 1.13 (s, 9H, Si*t*-Bu), 1.09-1.00 (m, 1H, 13-Hb), 1.09 (d, *J* = 6.4 Hz, 3H, 10-Me), 0.92 (s, 9H, Si*t*-Bu), 0.90 (s, 9H, Si*t*-Bu), 0.56 (d, *J* = 6.2 Hz, 3H, 14-Me), 0.09 (s, 3H, SiMe), 0.06 (s, 6H, SiMe), 0.04 (s, 3H, SiMe) ppm. The crude material was directly employed in the next step.

Progeldanamycin derivate 30

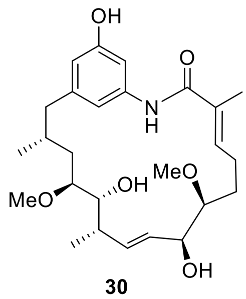

30

Progeldanamycin derivative **S27** (2.0 mg, 2.2 µmol, 1.0 eq.) was dissolved in THF (2.0 mL) under argon atmosphere and pyridine (500 µL) and HF*Py (500 µL) were added. After 7 days, the reaction was terminated by addition of an aqueous $NaHCO_3$ solution. The phases were separated and the aqueous phase was extracted three times with EtOAc. The combined organic phases were washed with $CuSO_4$ solution, dried over $MgSO_4$, filtered and concentrated under reduced pressure. The crude product was purified by preparative HPLC (C18 ISIS SP) (H_2O:MeOH = 90:10 {5 min}, gradient H_2O:MeOH = 90:10 → 40:60 {65 min}, H_2O:MeOH = 40:60 → 0:100 {20 min}, H_2O:MeOH = 0:100 {10 min}, 2.5 mL/min) (R_t = 36.5 min). Lactam **30** (0.6 mg, 1.3 µmol, 60%) and starting lactam **S27** (0.2 mg, 0.5 µmol, 20%) were obtained as colorless oils.
30: ^{1}H-NMR (500 MHz, MeOD, MeOD = 3.31 ppm): δ 6.89-6.80 (m, 1H, 3-H), 6.49 (s, 1H, Ph), 6.42 (s, 1H, Ph), 6.37 (s, 1H, Ph), 5.52-5.40 (m, 1H, 8-H), 5.52-5.40 (m, 1H, 9-H), 4.23 (d, *J* = 5.4 Hz, 1H, 7-H), 3.65-3.54 (m, 1H, 11-H), 3.41 (s, 3H, 6-OMe), 3.34 (s, 3H, 12-OMe), 3.34-3.20 (m, 1H, 12-H), 3.13-3.05 (m, 1H, H-6), 2.97-2.81 (m, 1H, 15-Ha), 2.46-2.34 (m, 1H, 4-Ha), 2.24-2.15 (m, 1H, 10-H), 2.15-2.10 (m, 1H, H-15b), 2.10-1.96 (m, 1H, 4-Hb), 1.94 (s, 3H, 2-Me), 1.80-1.70 (m, 1H, 14-H), 1.73-1.59 (m, 1H, 5-Ha), 1.73-1.59 (m, 1H, 13-Ha), 1.48-1.36 (m, 1H, 5-Hb), 1.27-1.19 (m, 1H, 13-Hb), 1.12 (d, *J* = 6.4 Hz, 3H, 10-Me), 0.76 (d, *J* = 6.2 Hz, 3H, 14-Me); ^{13}C-NMR (125 MHz, MeOD, MeOD = 49.0 ppm): δ 166.4 (d, C-1), 159.4 (s, C-Ar), 146.5 (d, C-3), 139.4 (s, C-Ar), 137.2 (d, C-9), 137.0 (s, C-2), 133.8 (s, C-Ar), 130.4 (d, C-8), 120.7 (d, C-Ar), 115.2 (d, C-Ar), 109.3 (d, C-Ar), 86.3 (d, C-6), 83.3 (d, C-12), 74.0 (d, C-11), 74.0 (d, C-7), 58.1 (q, 6-OMe), 58.1 (q, 12-OMe), 44.8 (t, C-15), 41.9 (d, C-10), 35.8 (t, C-13), 33.5 (d, C-14), 30.6 (t, C-4), 28.9 (t, C-5), 19.9 (q, 14-Me), 19.1 (q, 10-Me) 13.7 (q, 2-Me) ppm; HRMS (ESI) *m/z* for $C_{26}H_{39}NO_6Na$ $[M+Na]^+$: calculated: 484.2675, found: 484.2669.

4. Biotransformation of SNAC ester 27b using amide synthase *Sh*GdmF

SNAC ester **27b** (0.2 mg, 0.4 µmol,) in Hepes-buffer (25 mM Hepes, 150 nM NaCl, pH 6.8) was incubated with *Sh*GdmF for 24 h at 37°C and the reaction mixture was extracted with ethylacetate (5 mL). The phases were separated and the ethyl acetate extract was analyzed by mass spectrometry that revealed formation of macrolactam **30** as well as the hydrolysis product **29** (Figure S7).

Figure S8: Mass spectra of the enzymatic reaction with SNAC ester **27b**; a: negative control, b: progeldanamycin derivative **30** [M+Na], c: *seco* acid **31** [M+H], d: SNAC ester **27b** [M+H].

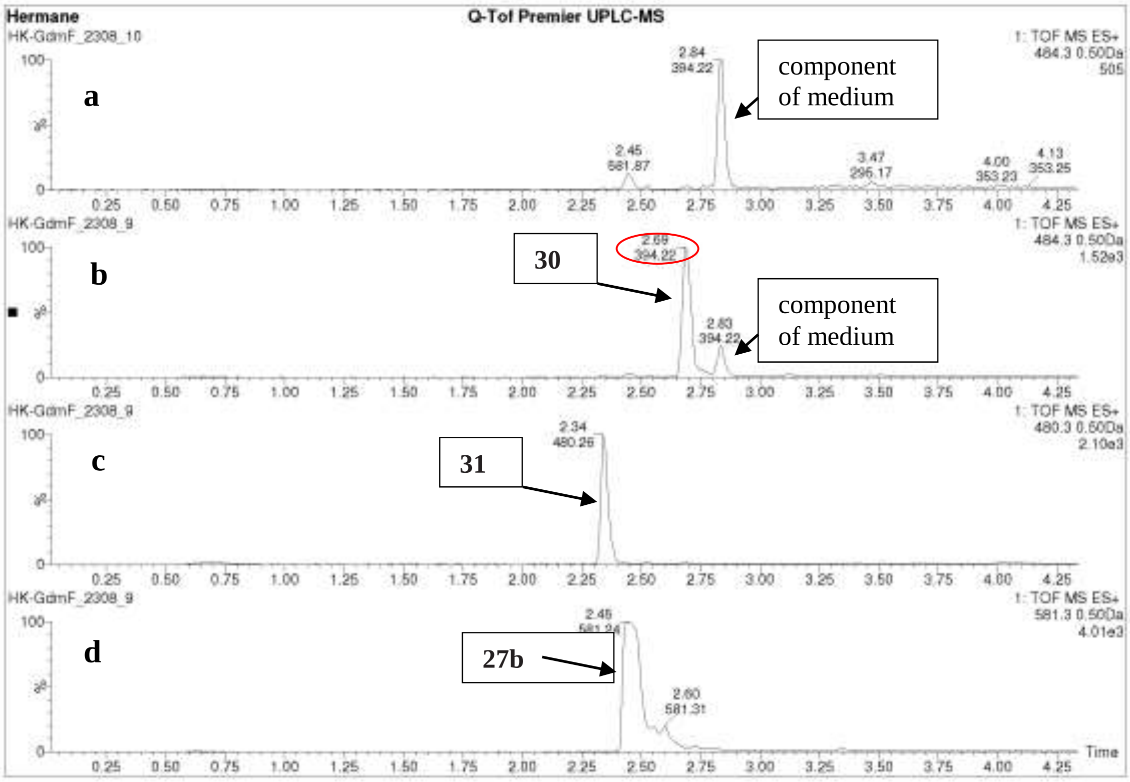

The structure of progeldanamycin derivative **30** was confirmed by MS/MS fragmentation experiment (Figure S8).

Figure S9: MS-MS fragmentation experiment of progeldanamycine derivative **30**.

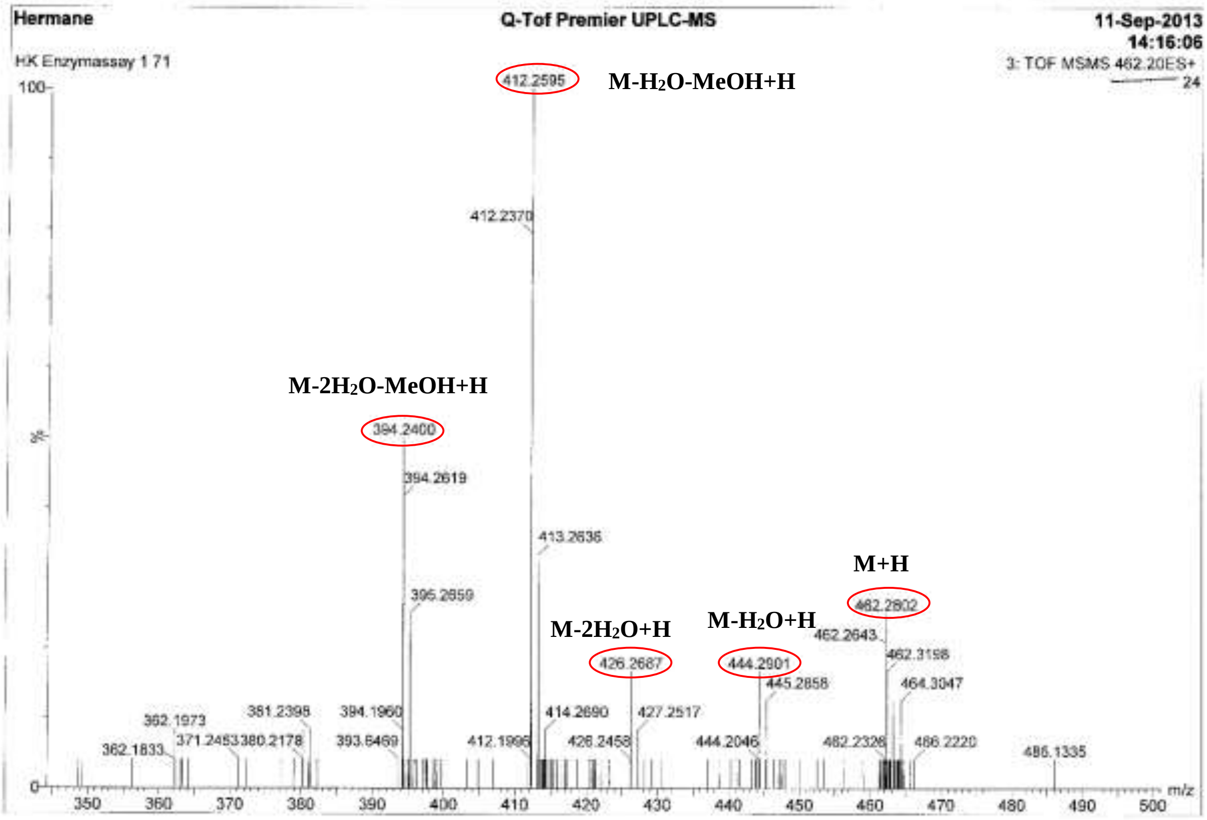

5. References (Supporting information)

[S1] N. M. Gaudelli, C. A. Townsend, *J. Org. Chem.* **2013**, *78*, 6412–6426.

[S2] D. M. Roberts, C. Bartel, A. Scott, D. Ivison, T. J. Simpson, R. J. Cox, *Chem. Sci.* **2017**, *8*, 1116–1126.

[S3] F. Pfrengle, V. Dekaris, L. Schefzig, R. Zimmer, H. U. Reissig, *Synlett* **2008**, 2965–2968.

[S4] P. D. Walker, C. Williams, A. N. M. Weir, L. Wang, J. Crosby, P. R. Race, T. J. Simpson, C. L. Willis, M. P. Crump, *Angew. Chem. Int. Ed.* **2019**, *58*, 12446–12450.

[S5] O. Vergnolle, F. Hahn, A. Baerga-Ortiz, P. F. Leadlay, J. N. Andexer, *ChemBioChem* **2011**, *12*, 1011–1014.

[S6] W. Seubert, F. Lynen, *J. Am. Chem. Soc.* **1953**, *75*, 2787–2788.

[S7] K. K. Sharma, C. N. Boddy, *Bioorg. Med. Chem. Lett.* **2007**, *17*, 3034–3037.

[S8] A. Dey, M. T. Kirchner, V. R. Vangala, G. R. Desiraju, R. Mondal, J. A. K. Howard, *J. Am. Chem. Soc.* **2005**, *127*, 10545–10559.

[S9] U. Ravid, R. M. Silverstein, L. R. Smith, *Tetrahedron* **1978**, *34*, 1449-1452.

[S10] R. A. Pilli, M. M. Victor, A. de Meijere, *J. Org. Chem.* **2000,** *65*, 5910-5916.

[S11] T. R. Hoye, C. S. Jeffrey, F. Shao, *Nat. Protoc.* **2007**, 2451-2458.

[S12] G. E. Keck, J. A. Murry, *J. Org. Chem.* **1991**, *56*, 6606-6611.

Attachment: Copies of ^{1}H- and ^{13}C-NMR spectra

Figure S10: ^{1}H NMR spectrum of compound **S15**.

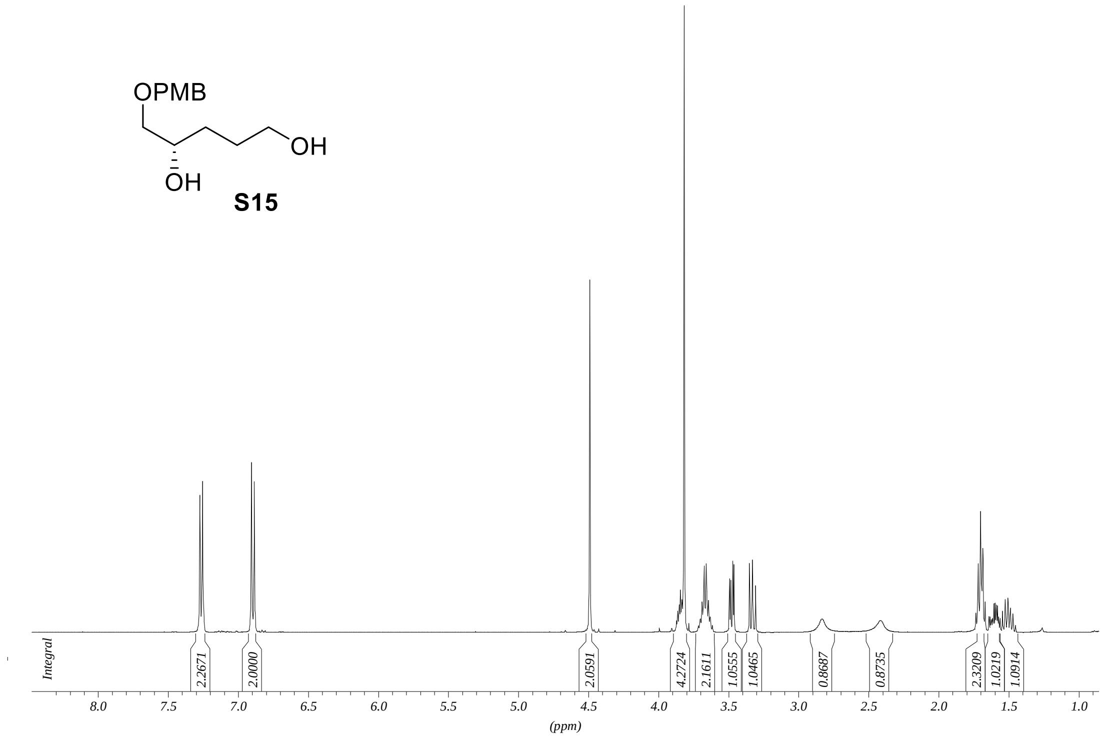

Figure S11: ^{13}C NMR spectrum of compound **S15**.

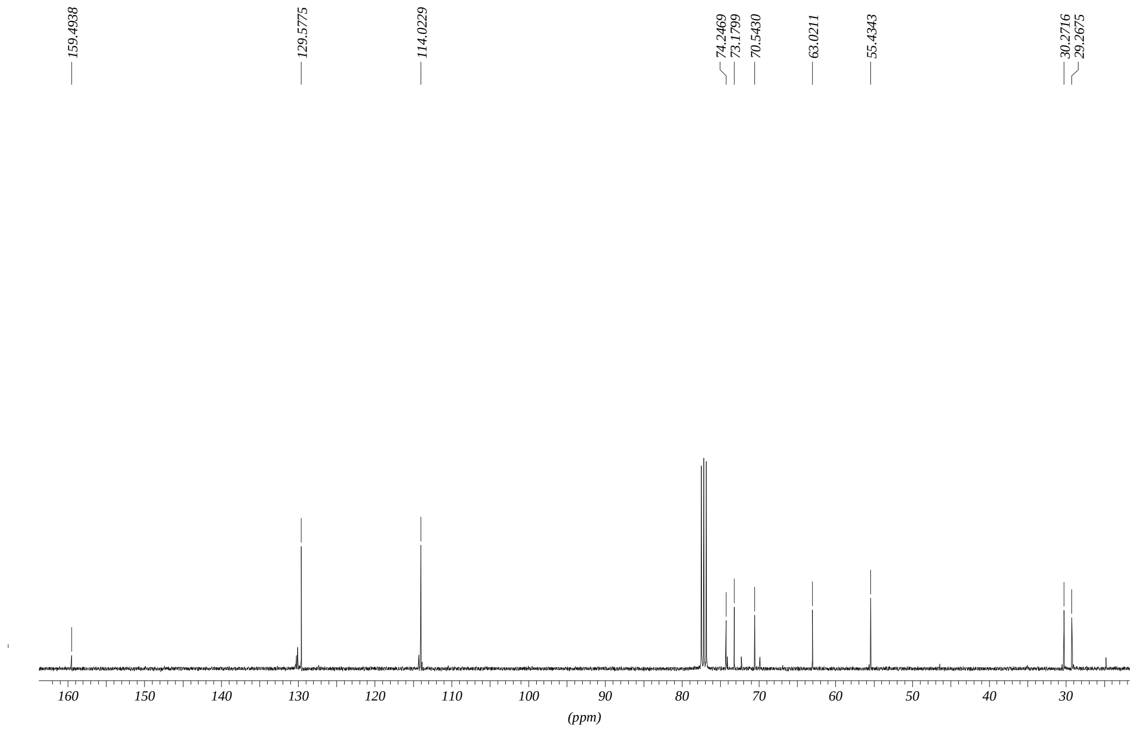

Figure S12: ^{1}H NMR spectrum of compound **S16**.

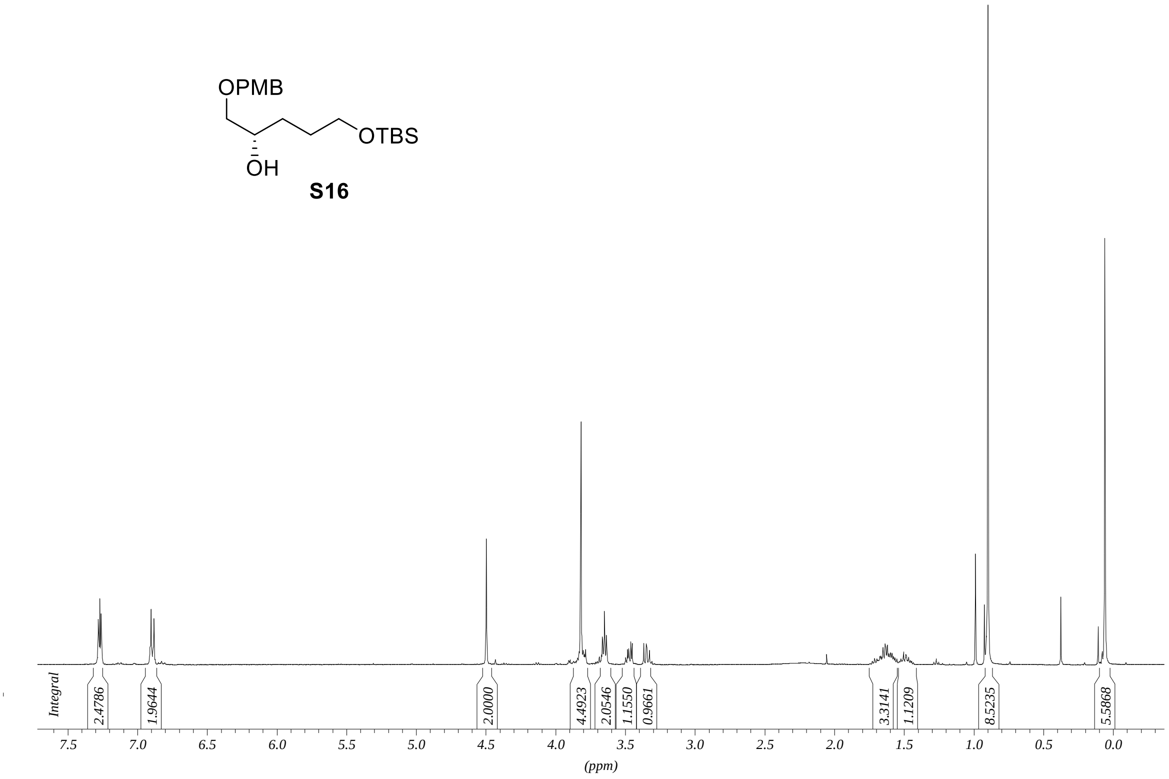

Figure S13: ^{13}C NMR spectrum of compound **S16**.

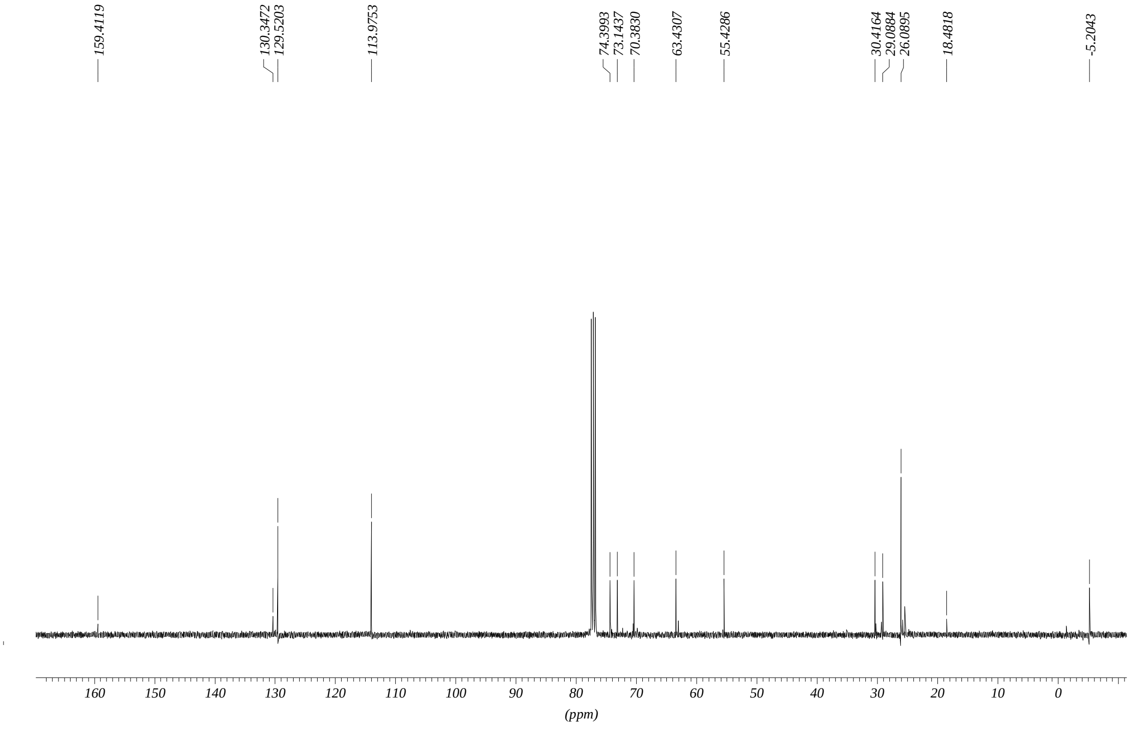

Figure S14: ^{1}H NMR spectrum of compound **S17**.

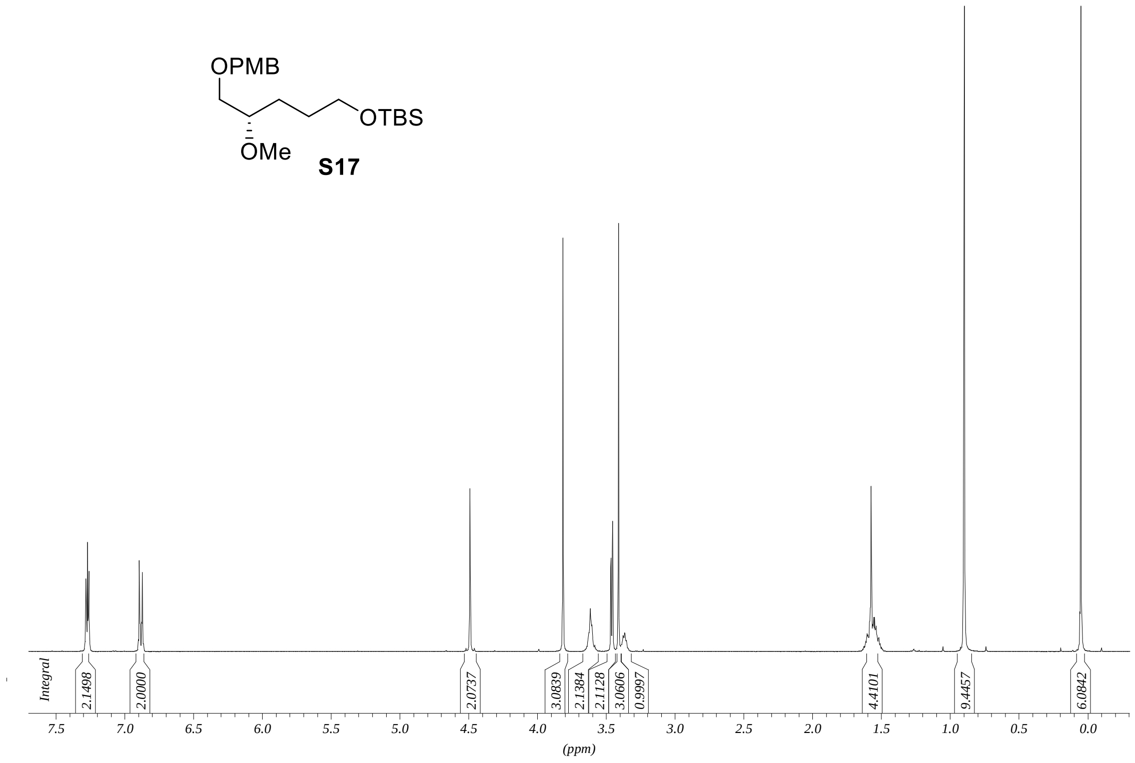

Figure S15: ^{13}C NMR spectrum of compound **S17**.

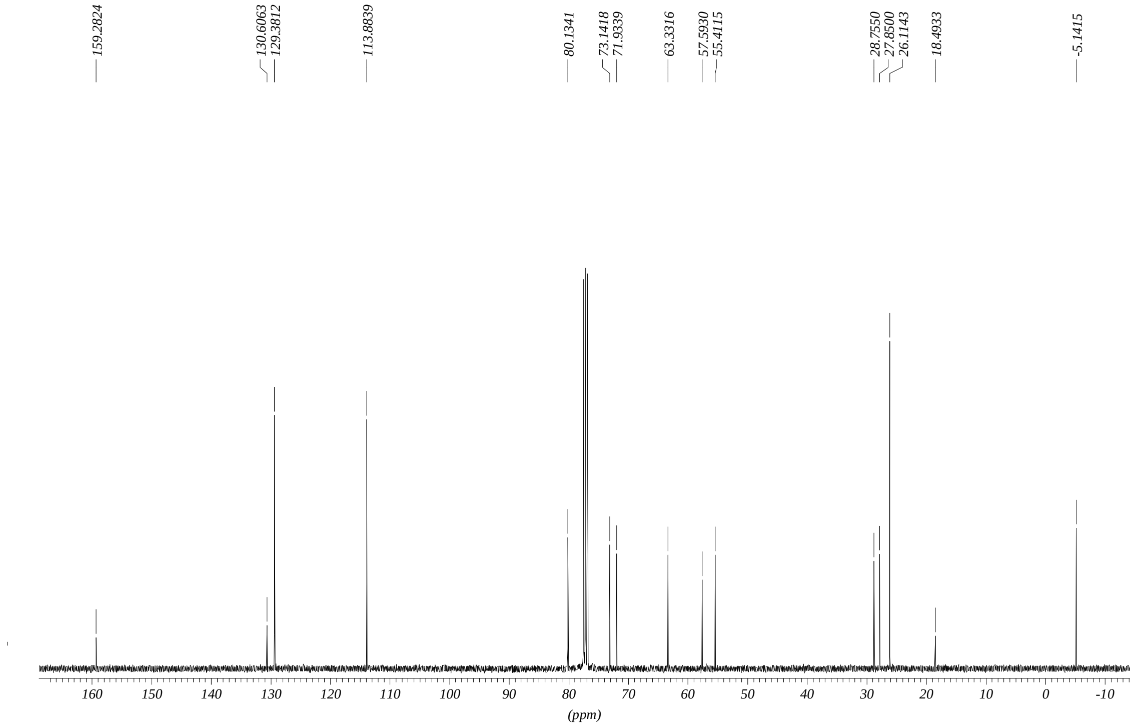

Figure S16: ^{1}H NMR spectrum of compound **S18**.

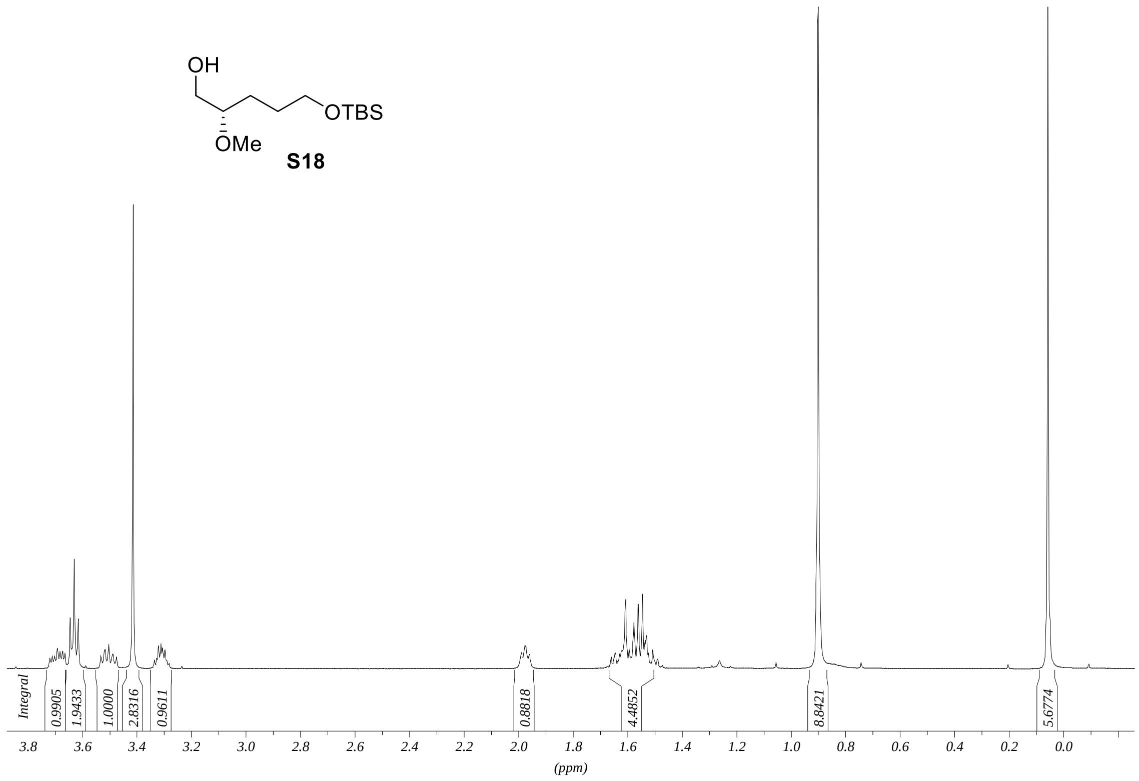

Figure S17: ^{13}C NMR spectrum of compound **S18**.

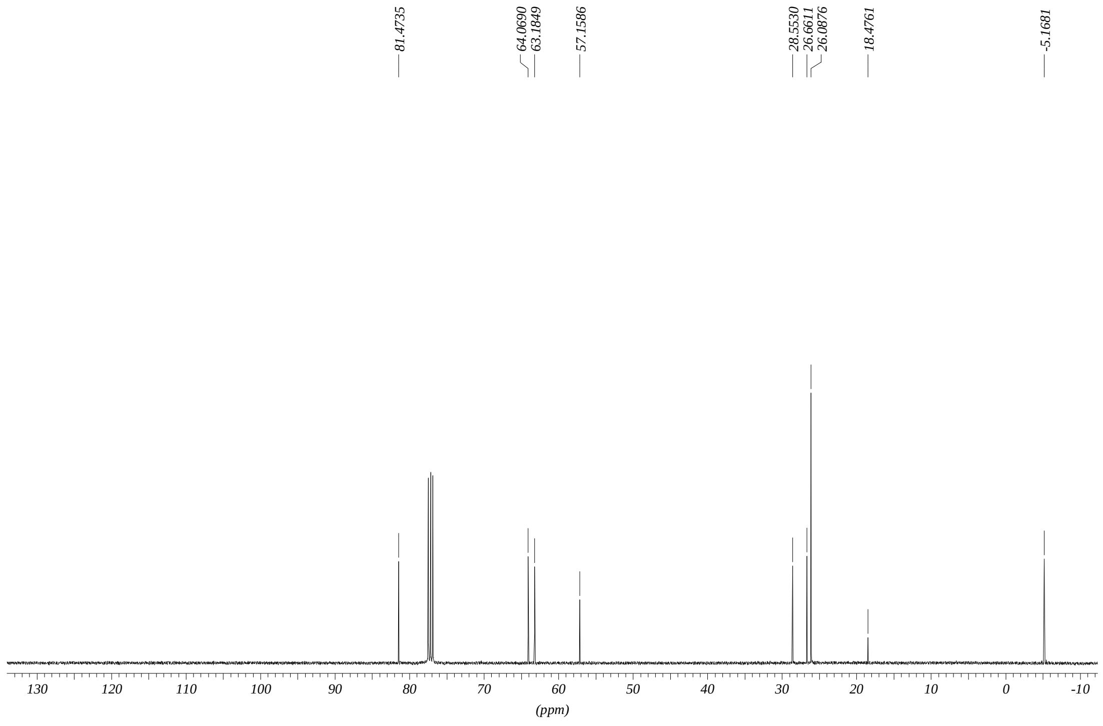

Figure S18: ^{1}H NMR spectrum of compound **S19a**.

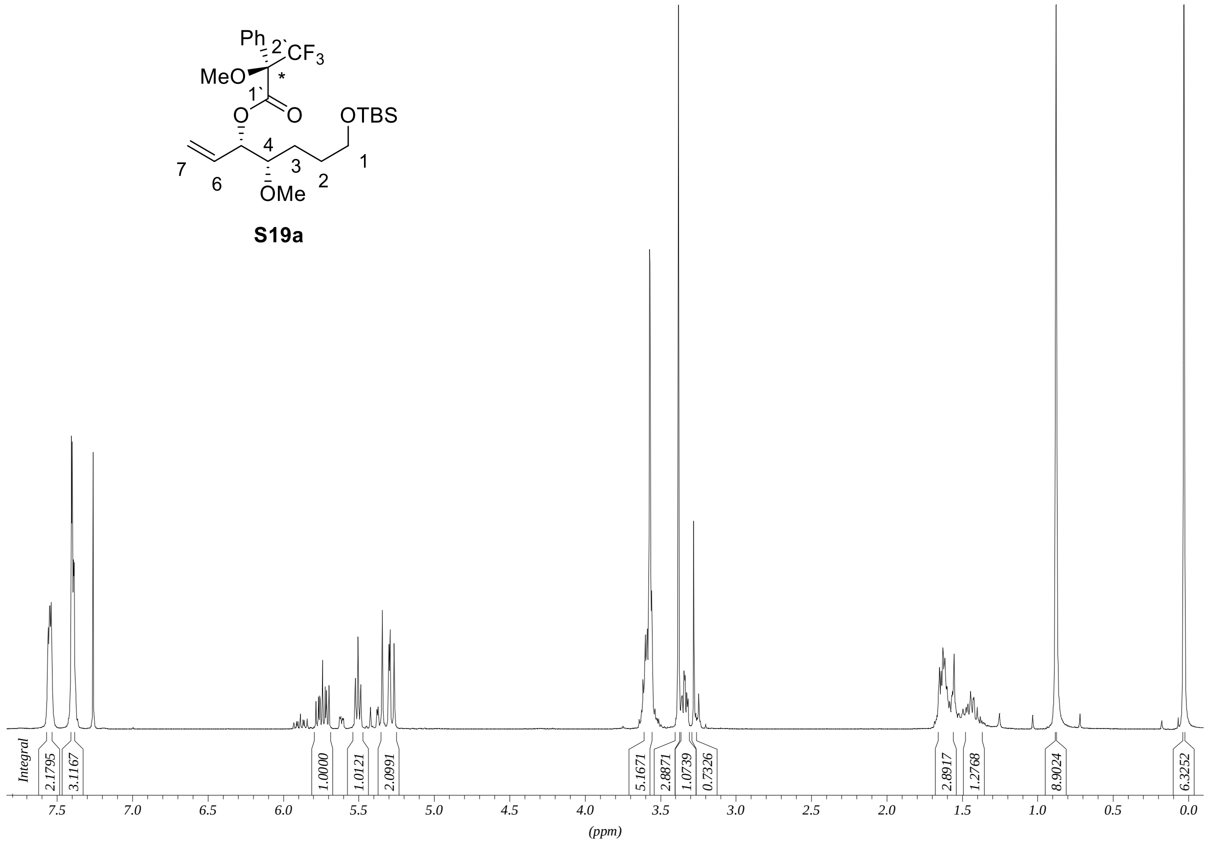

Figure S19: ^{1}H NMR spectrum of compound **S19b**.

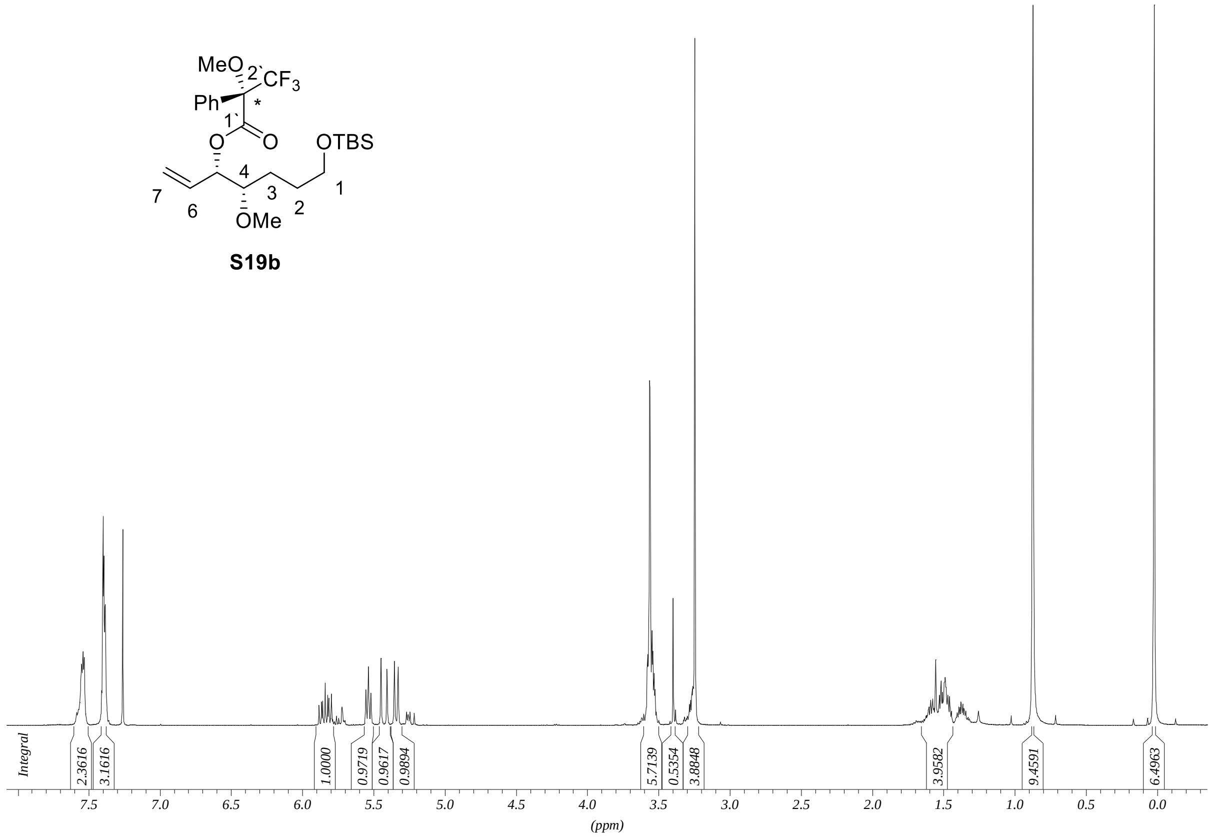

Figure S20: ^{1}H NMR spectrum of compound **24**.

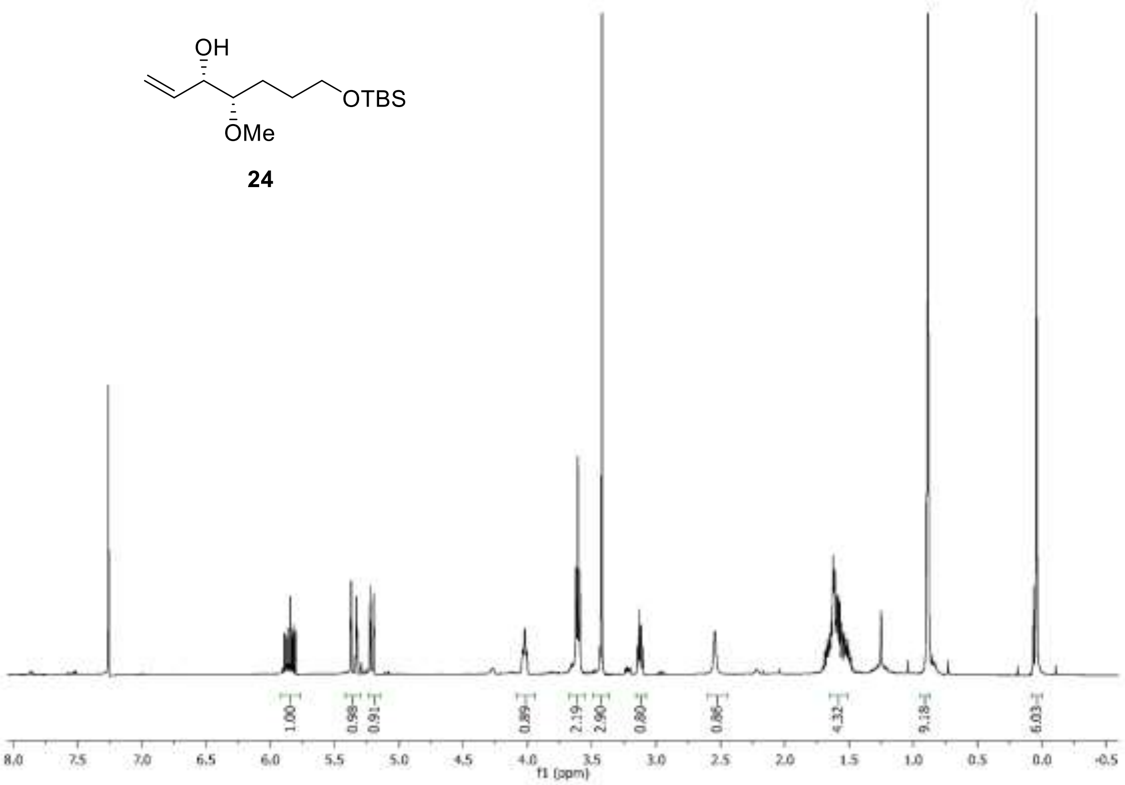

Figure S21: ^{13}C NMR spectrum of compound **24**.

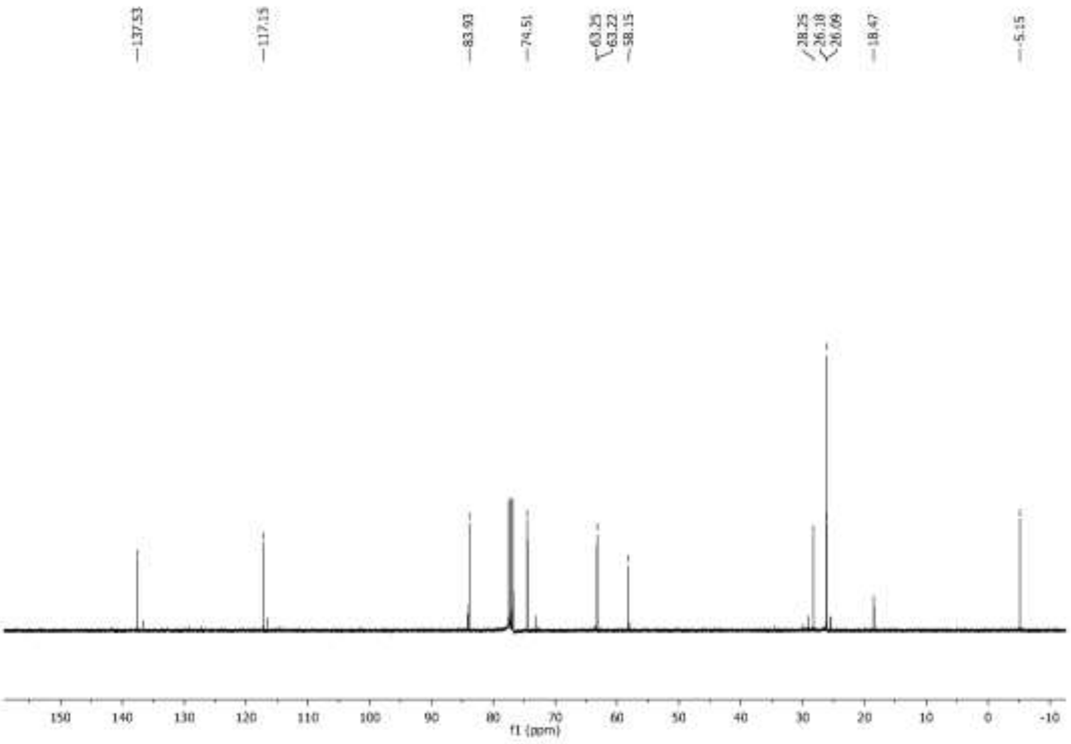

Figure S22: ^{1}H NMR spectrum of compound **19**.

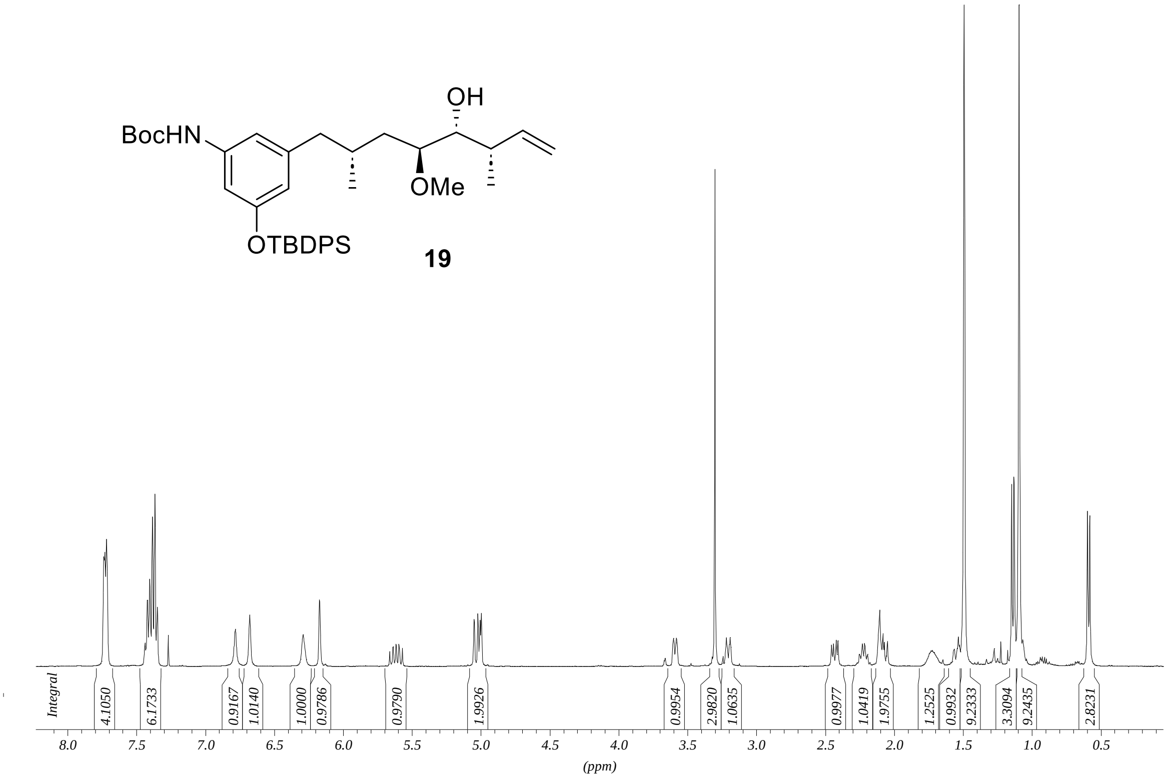

Figure S23: ^{13}C NMR spectrum of compound **19**.

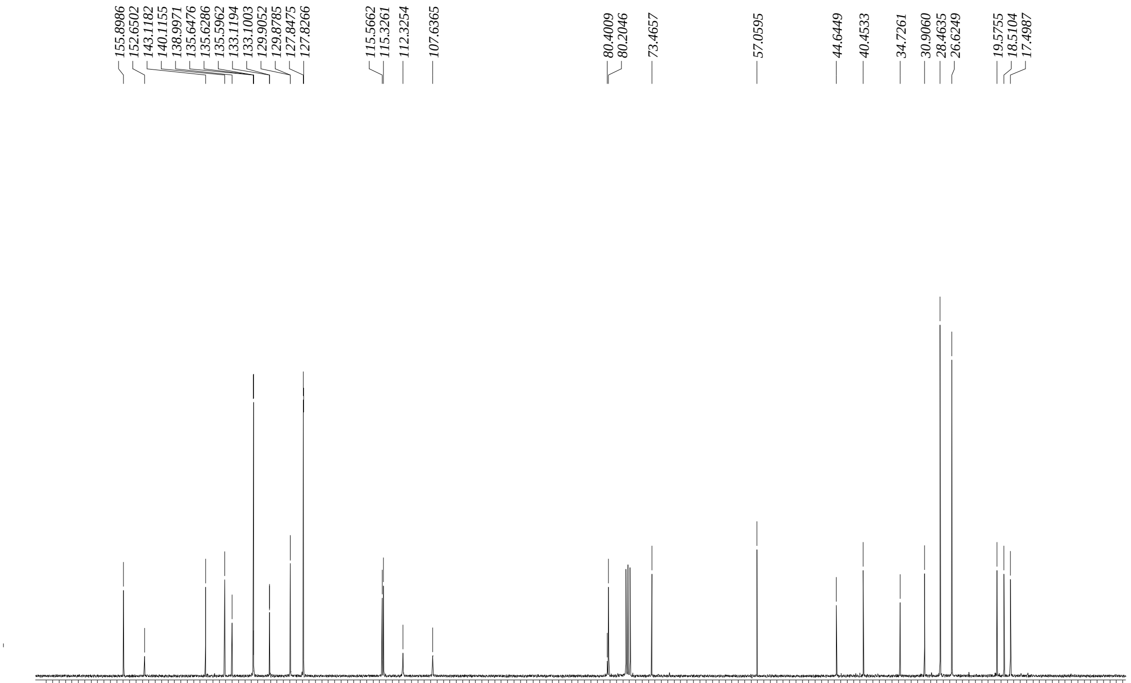

Figure S24: ^{1}H NMR spectrum of compound **25**.

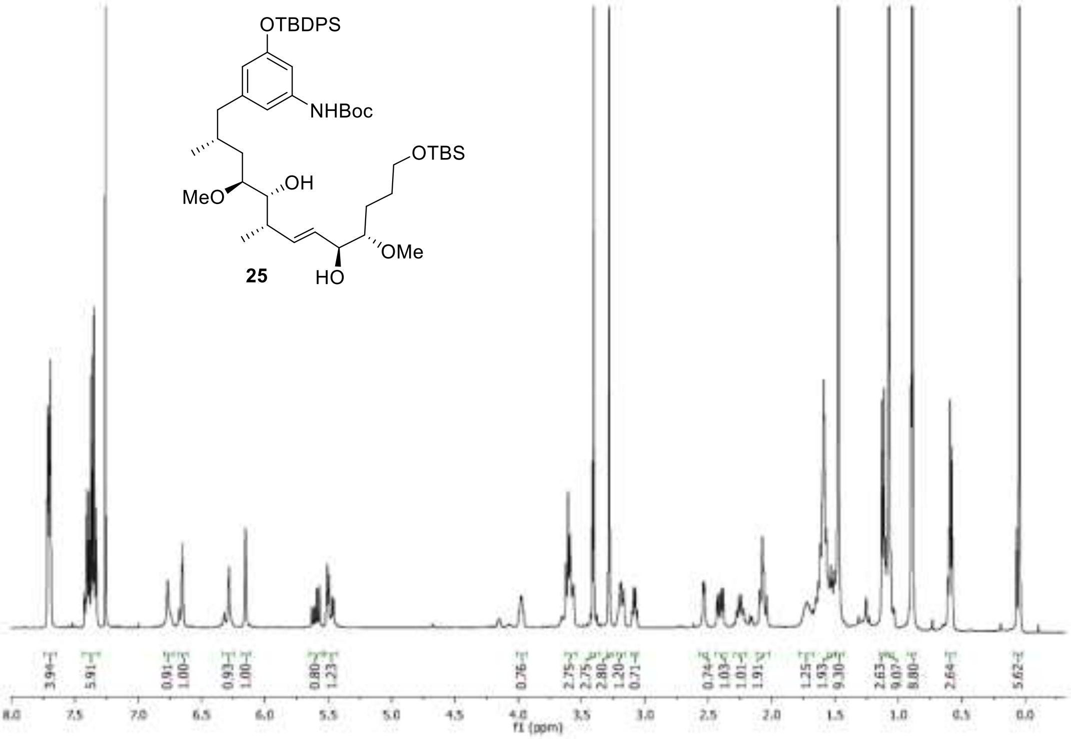

Figure S25: ^{13}C NMR spectrum of compound **25**.

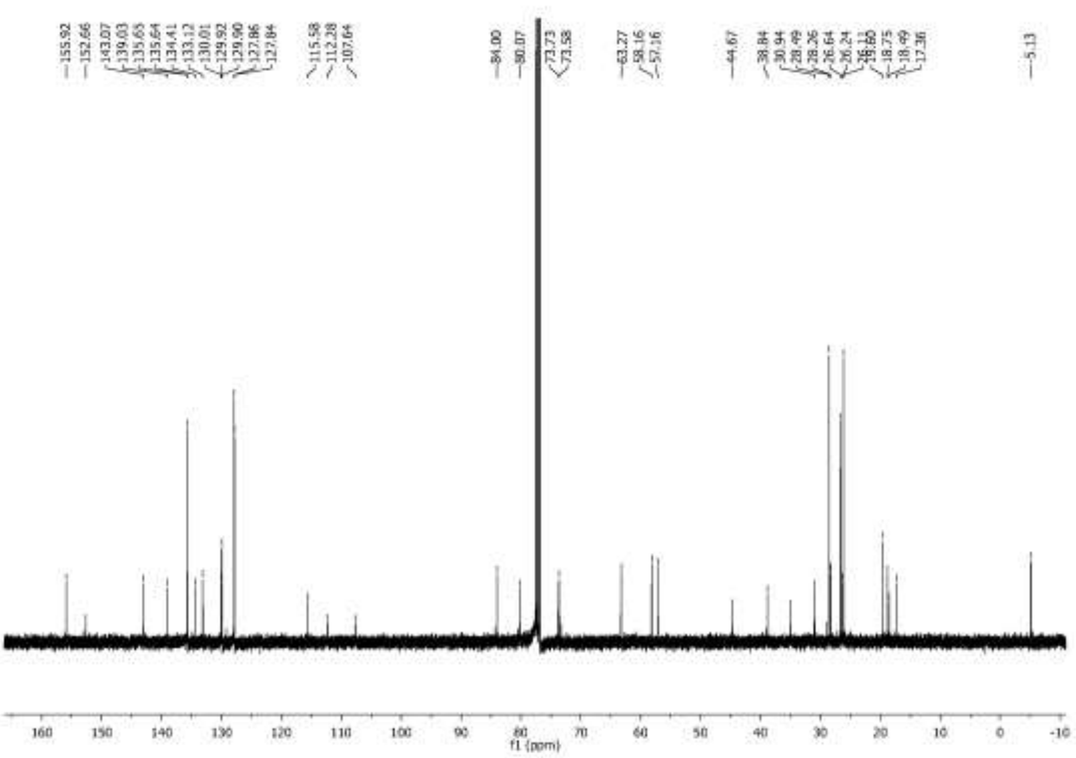

Figure S26: ^{1}H NMR spectrum of compound **S20**.

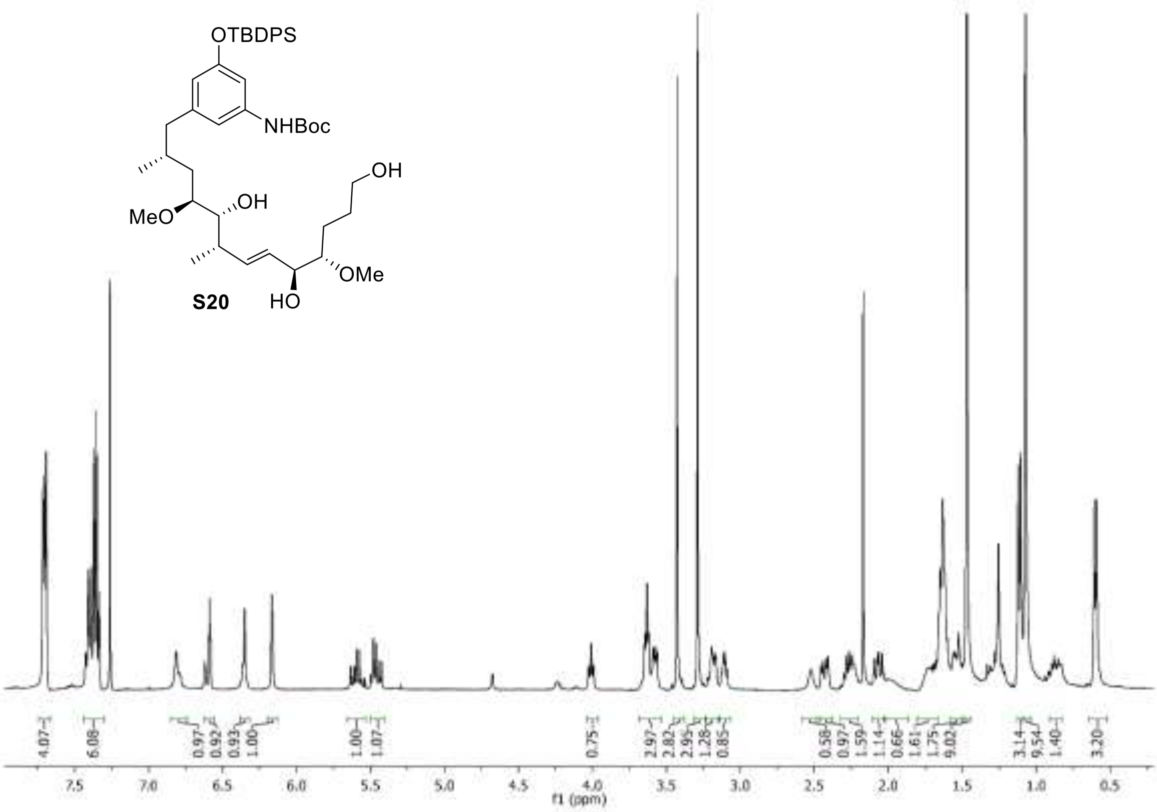

Figure S27: ^{13}C NMR spectrum of compound **S20**.

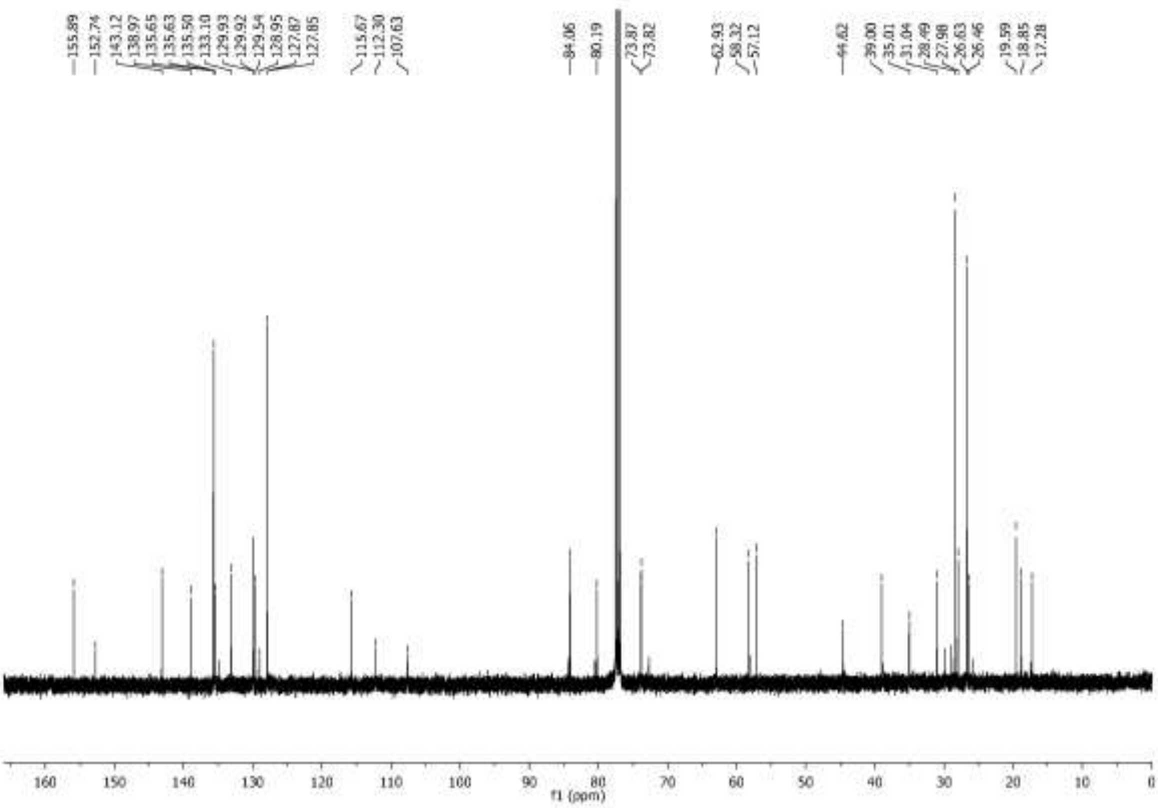

Figure S28: ^{1}H NMR spectrum of compound **S21**.

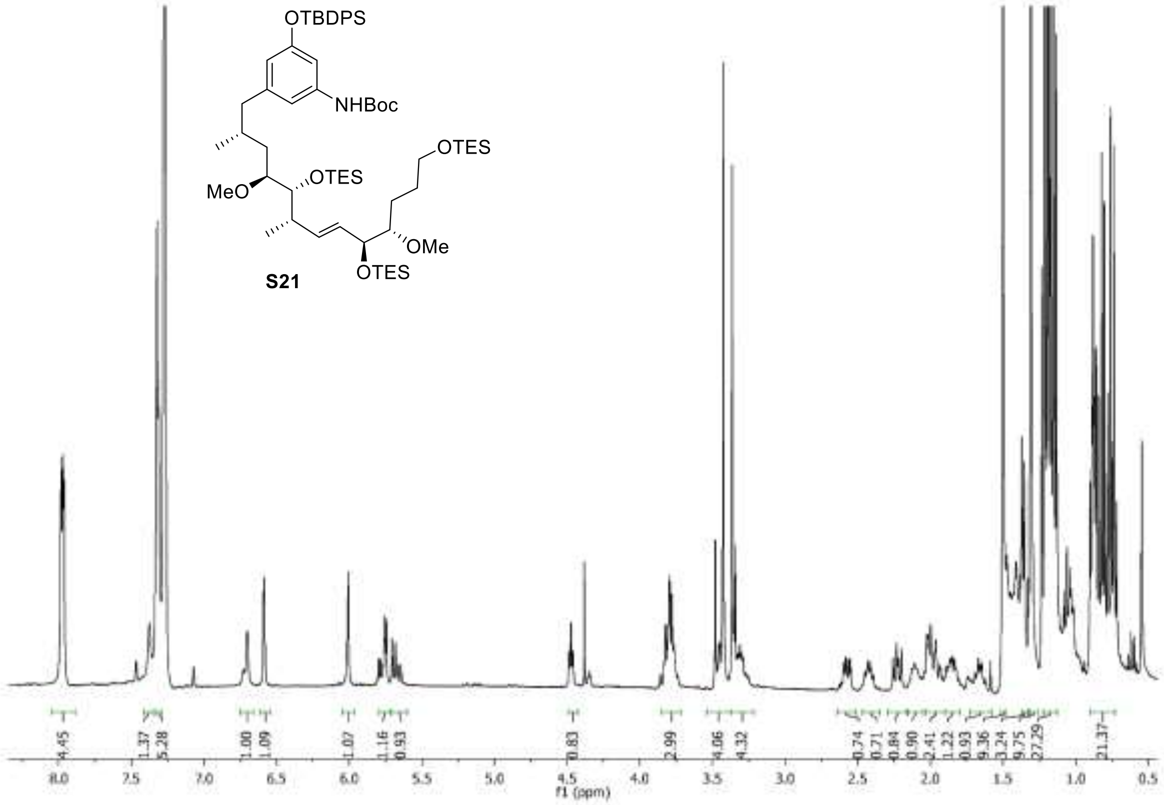

Figure S29: ^{13}C NMR spectrum of compound **S21**.

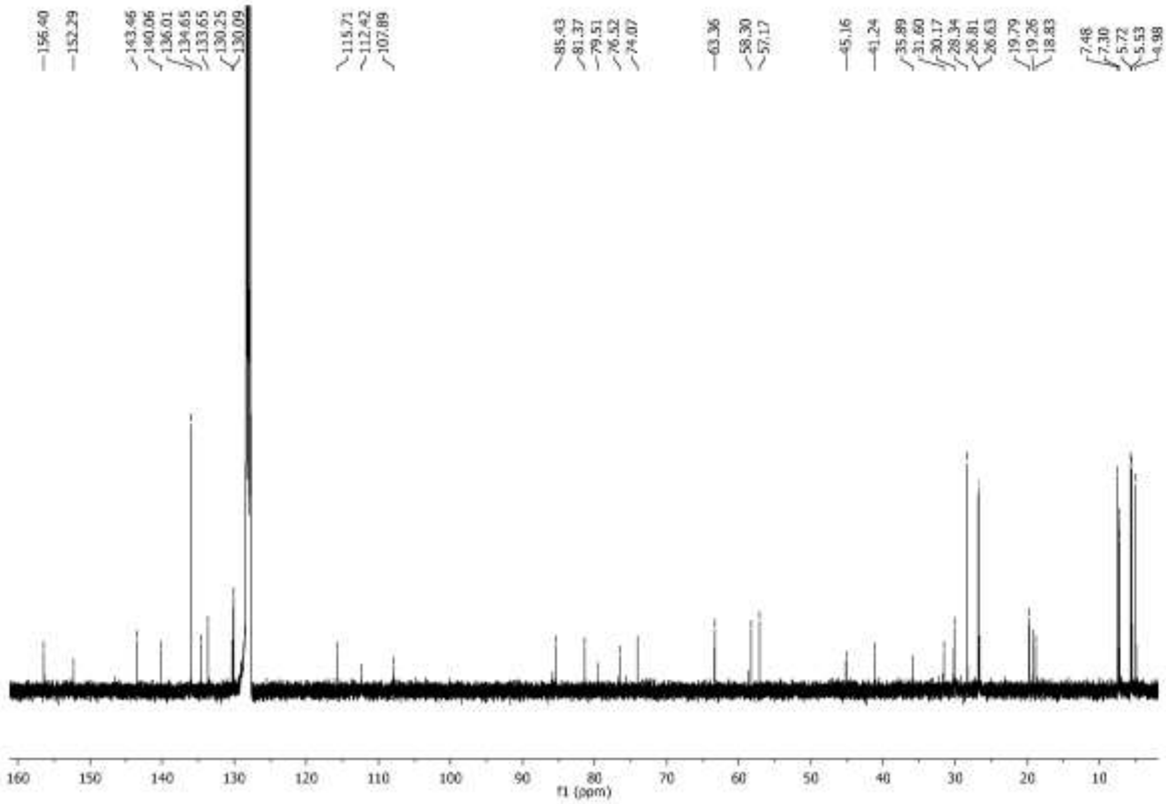

Figure S30: ^{1}H NMR spectrum of compound **S22**.

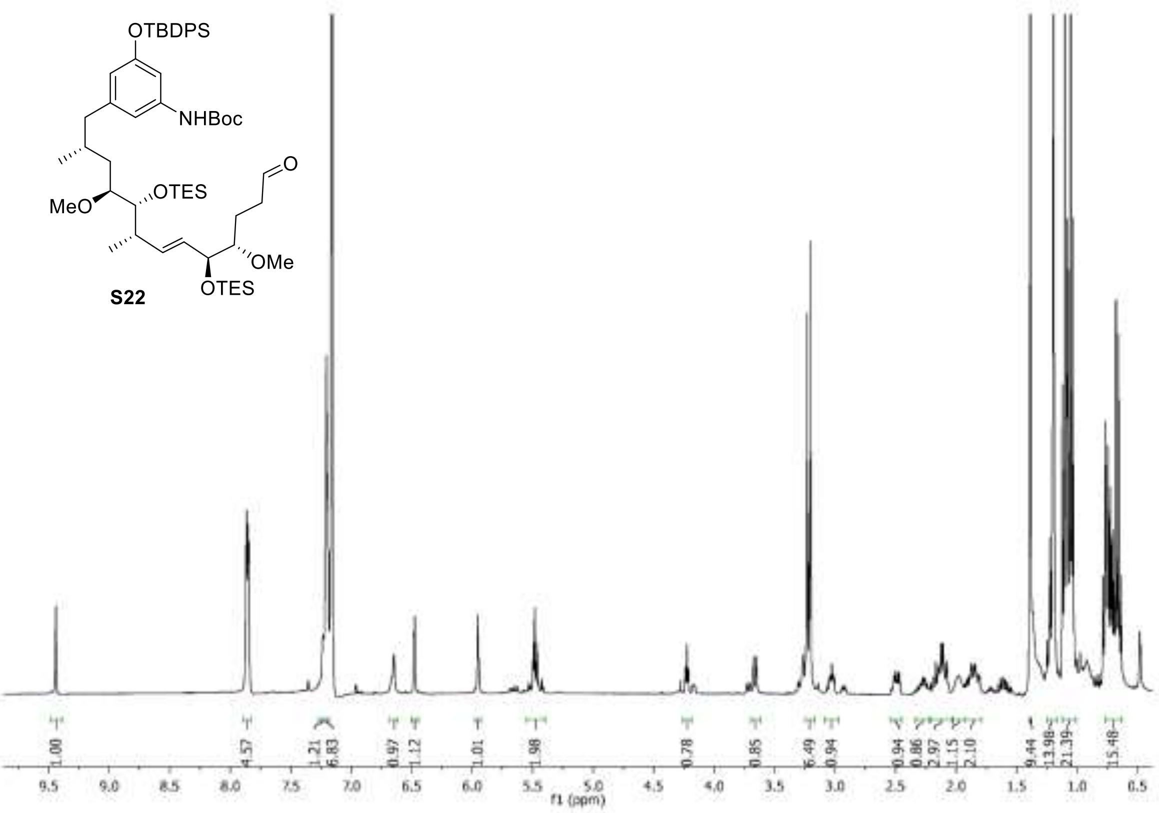

Figure S31: ^{13}C NMR spectrum of compound **S22**.

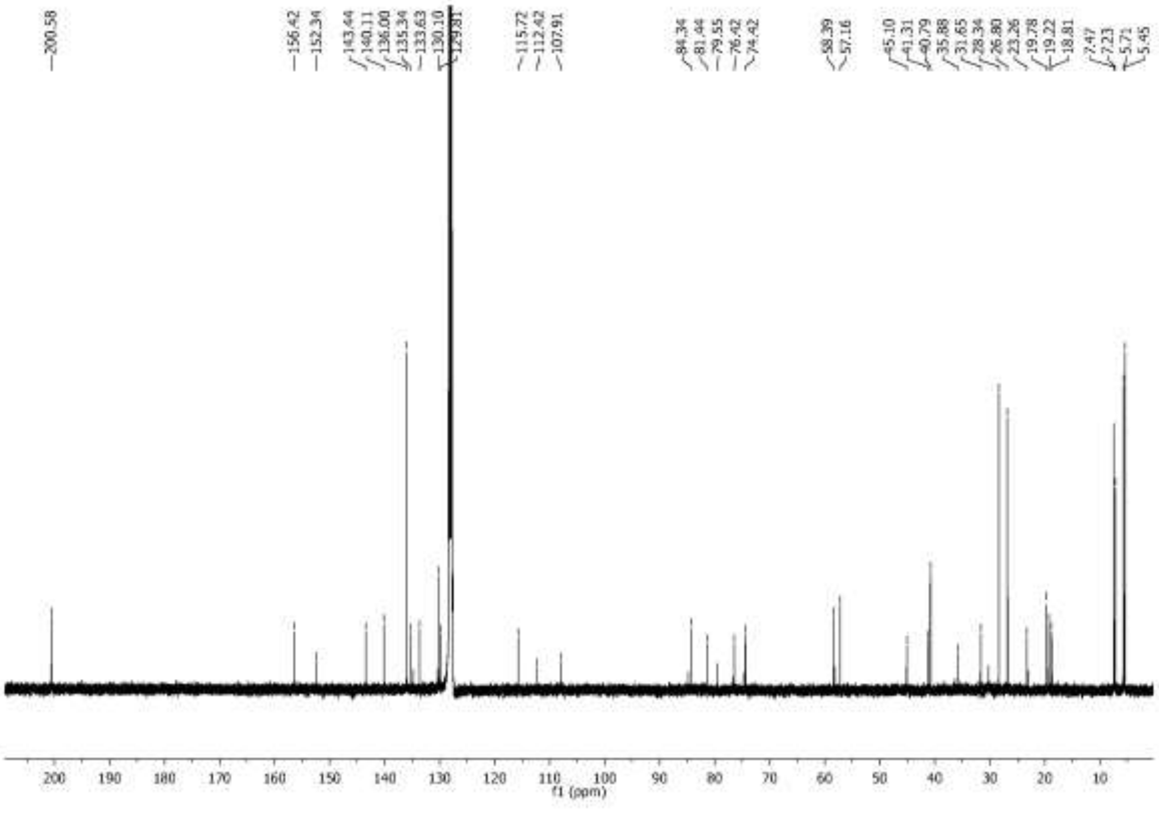

Figure S32: ^{1}H NMR spectrum of compound **27a**.

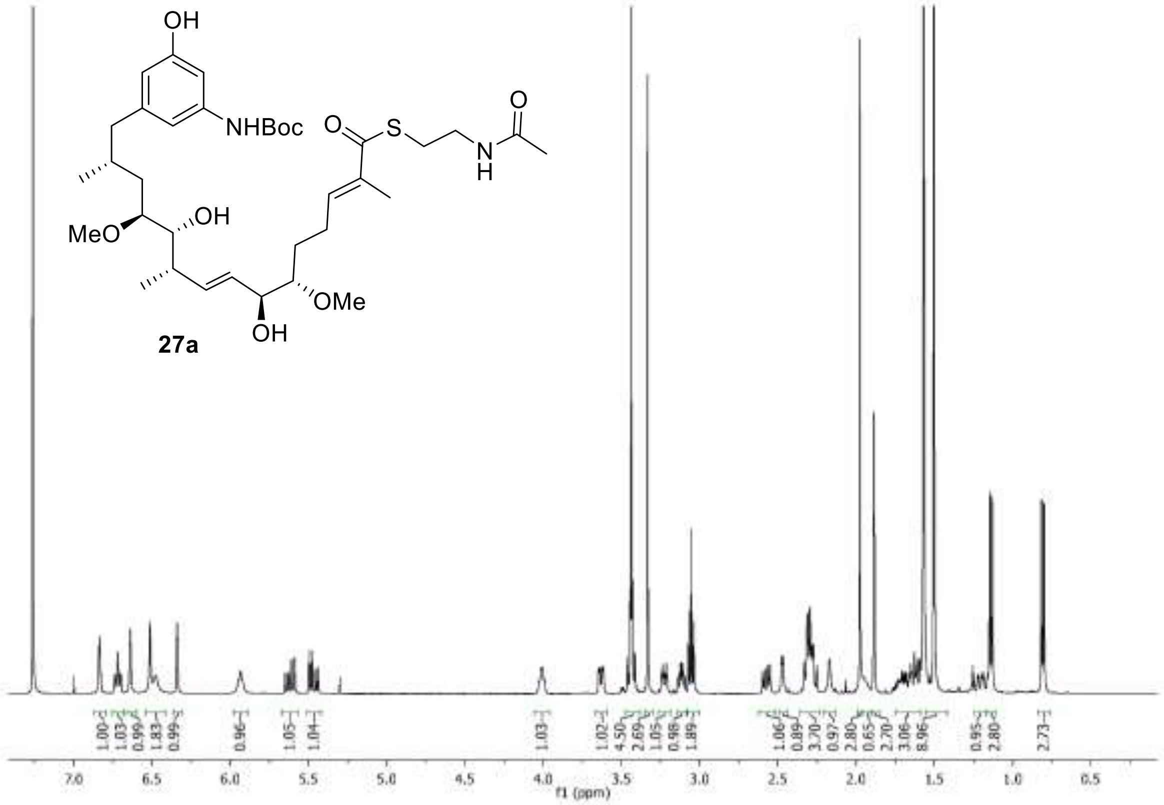

Figure S33: ^{13}C NMR spectrum of compound **27a**.

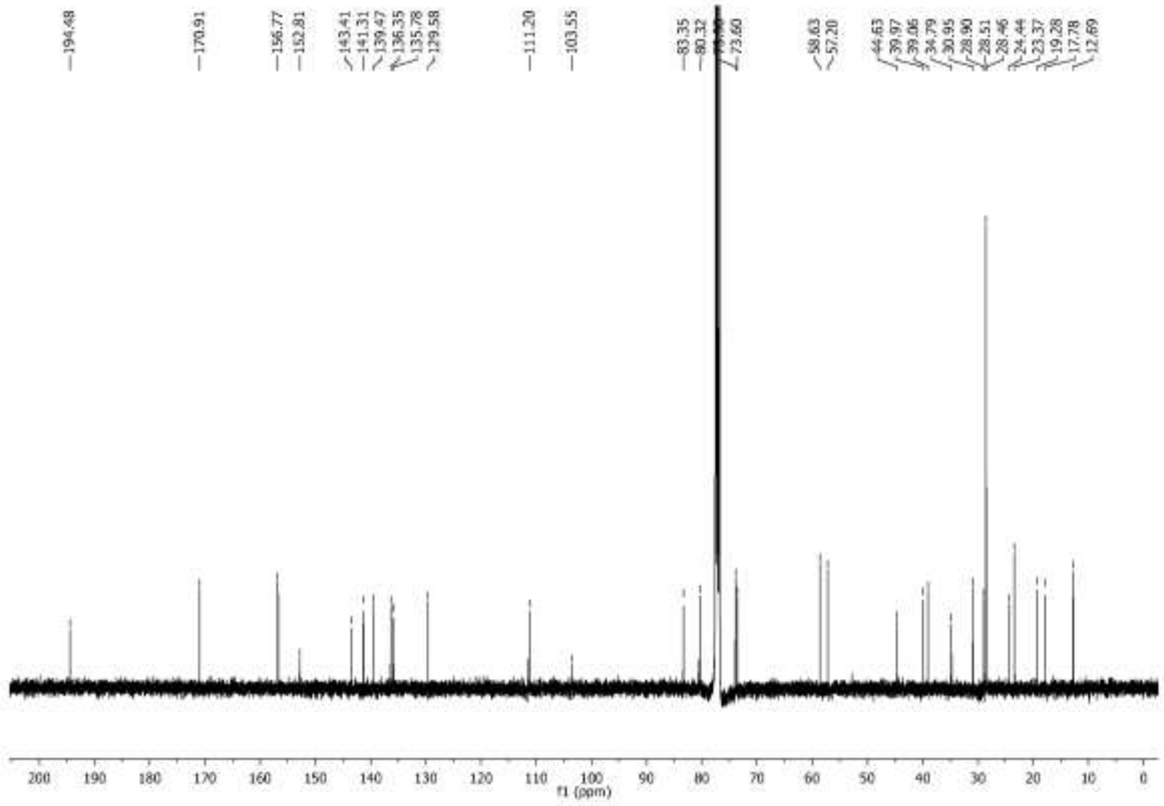

Figure S34: ^{1}H NMR spectrum of compound **27b**.

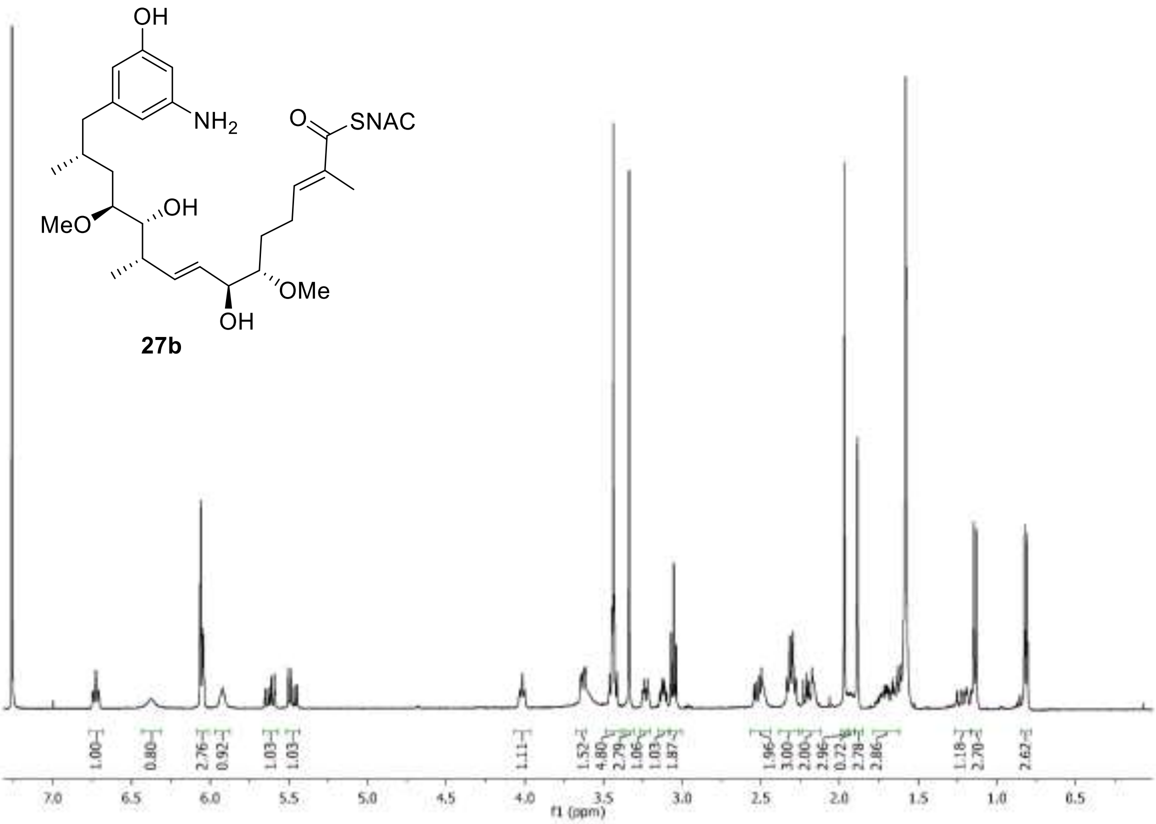

Figure S35: ^{13}C NMR spectrum of compound **27b**.

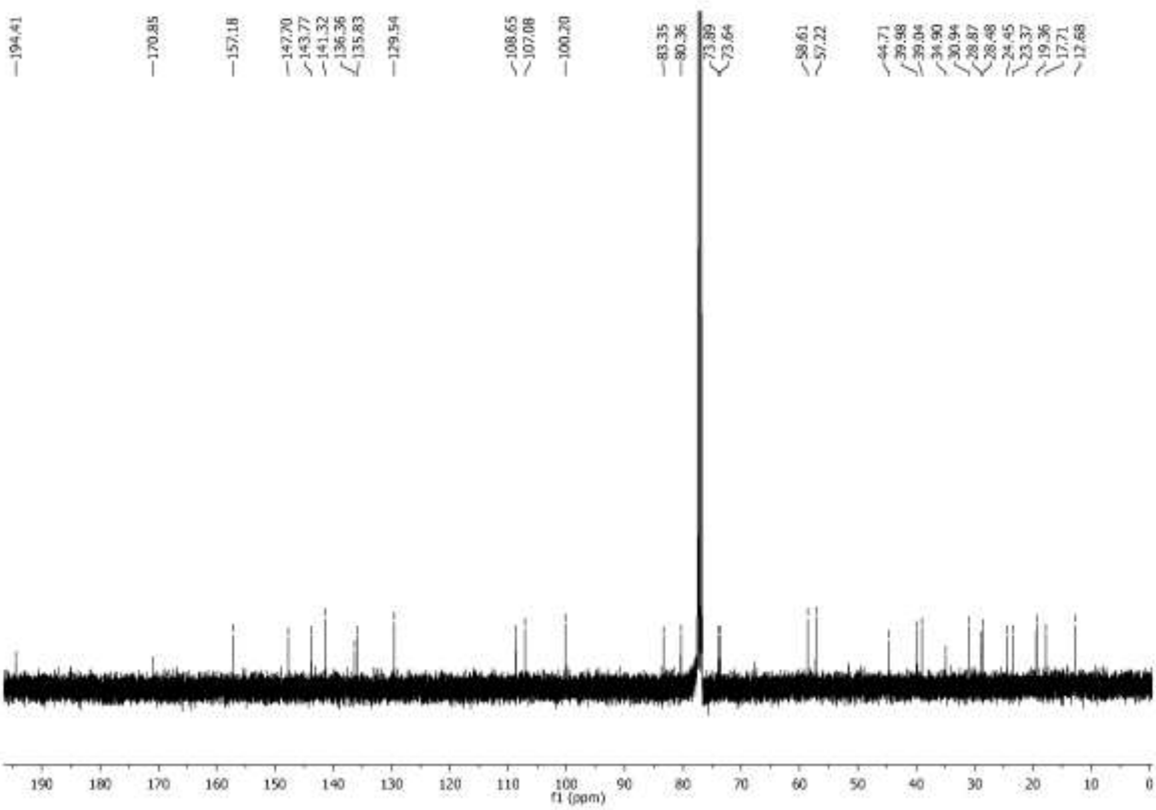

Figure S36: ^{1}H NMR spectrum of compound **S23**.

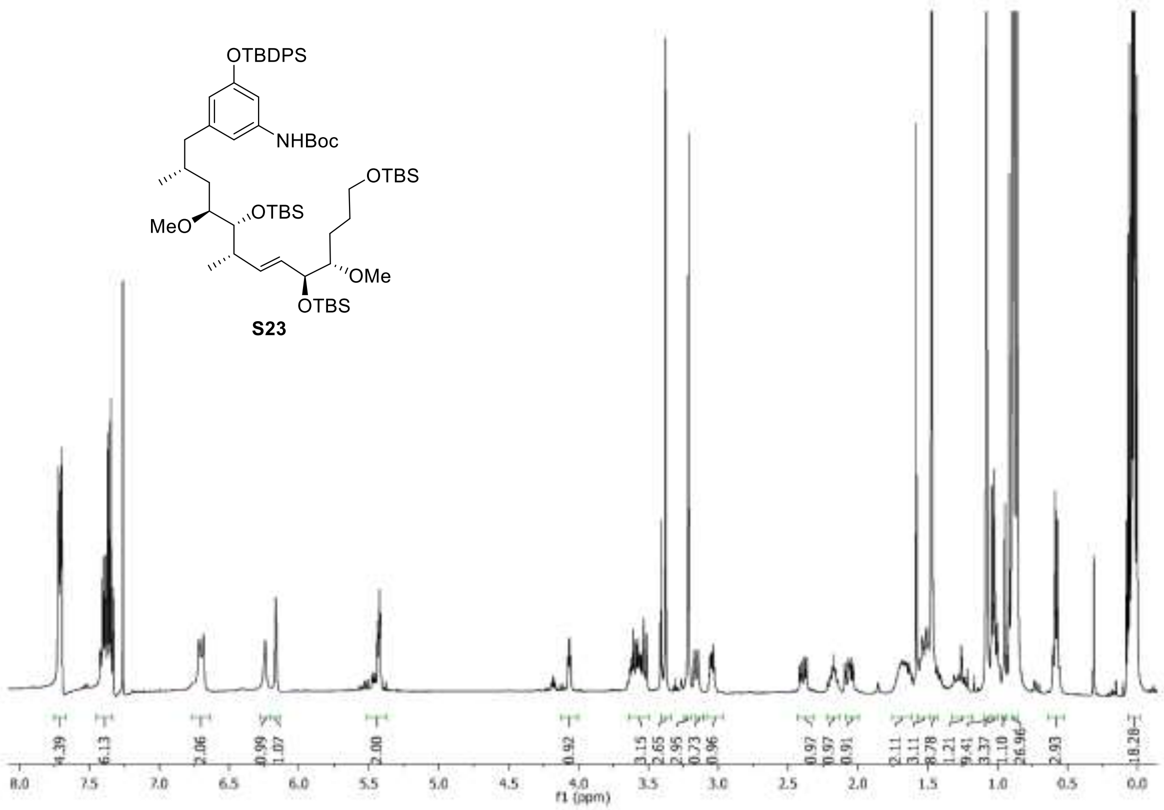

Figure S37: ^{13}C NMR spectrum of compound **S23**.

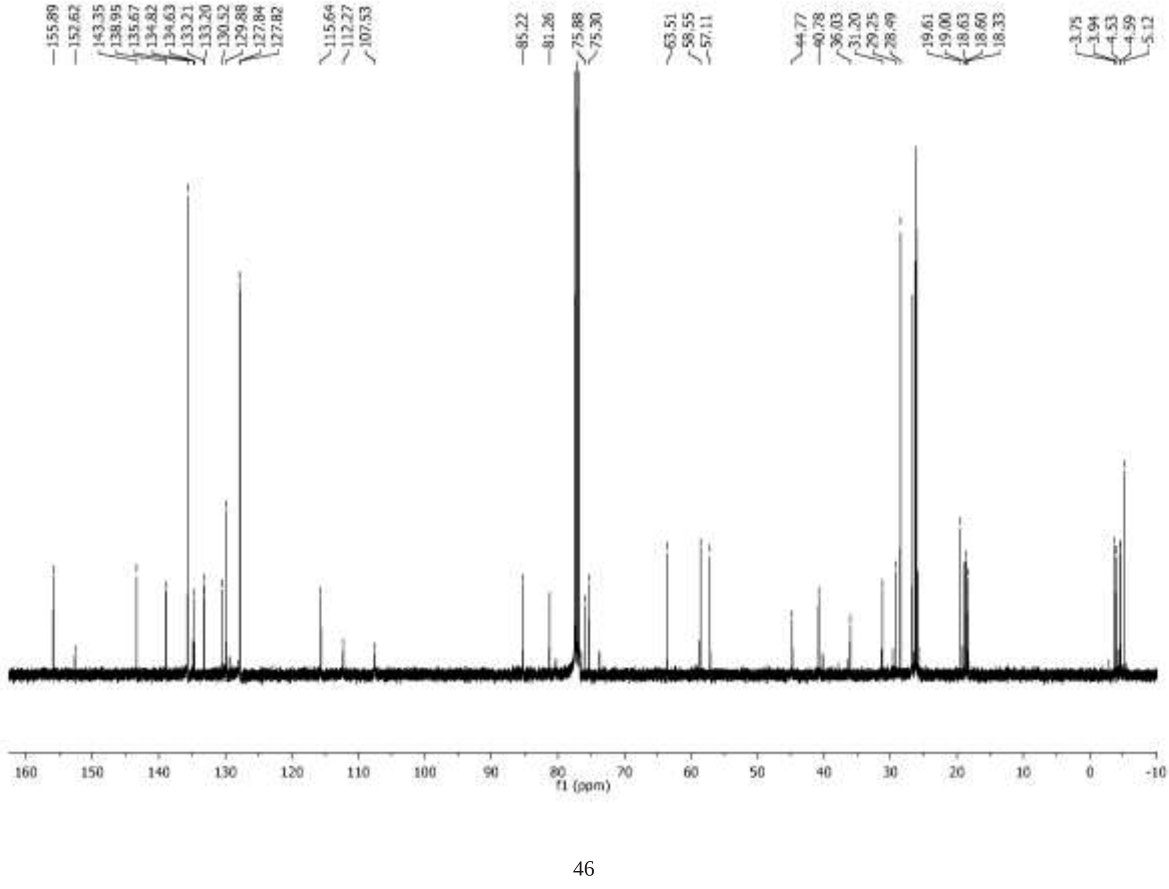

Figure S38: ^{1}H NMR spectrum of compound **S24**.

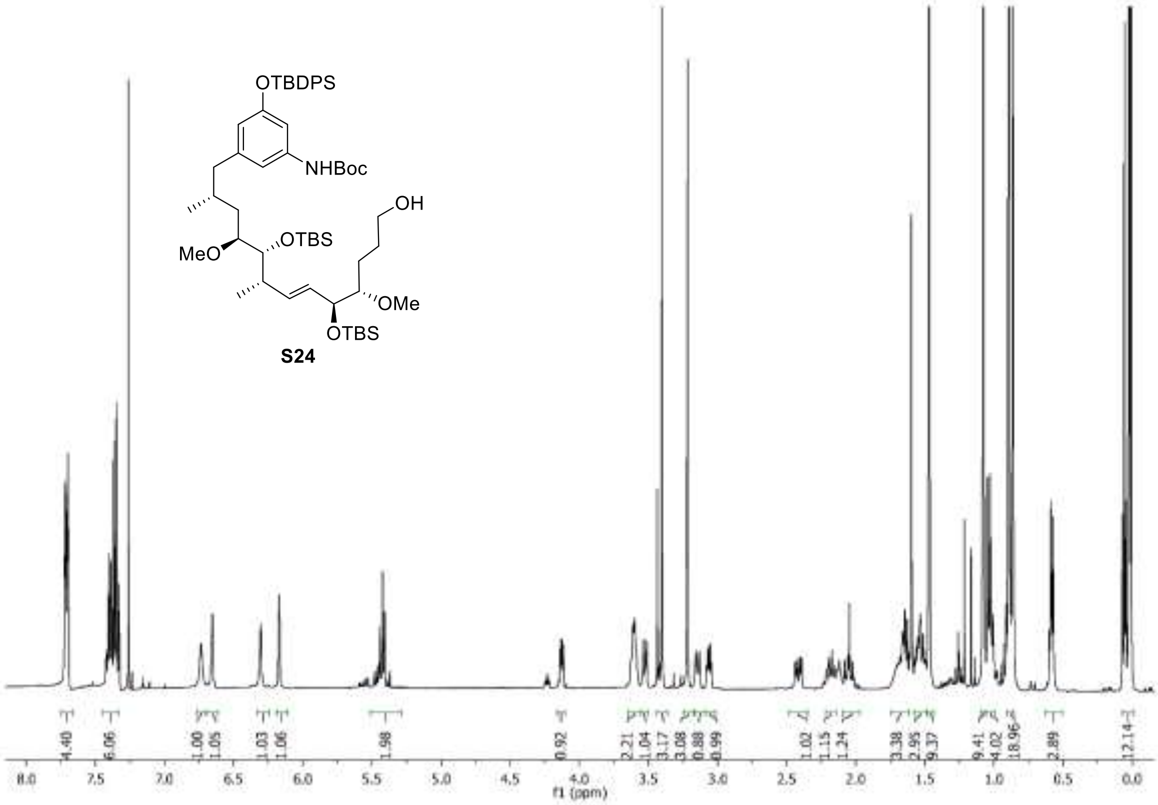

Figure S39: ^{13}C NMR spectrum of compound **S24**.

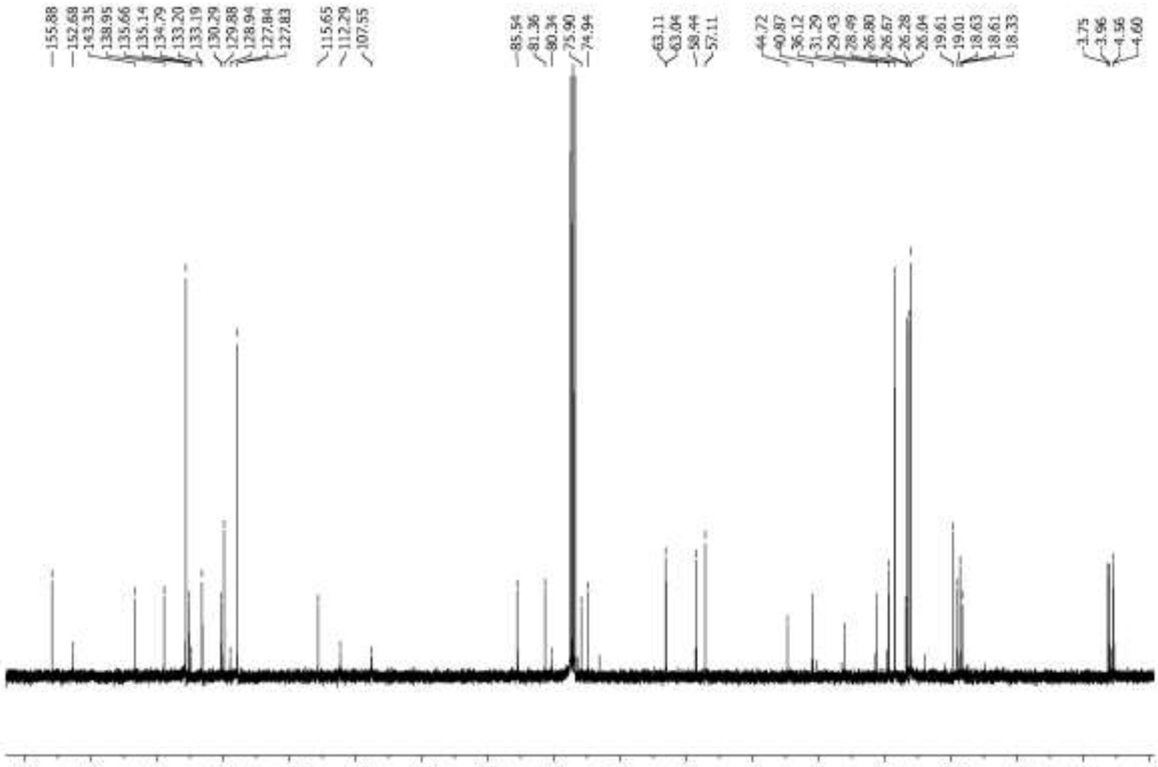

Figure S40: ^{1}H NMR spectrum of compound **S25**.

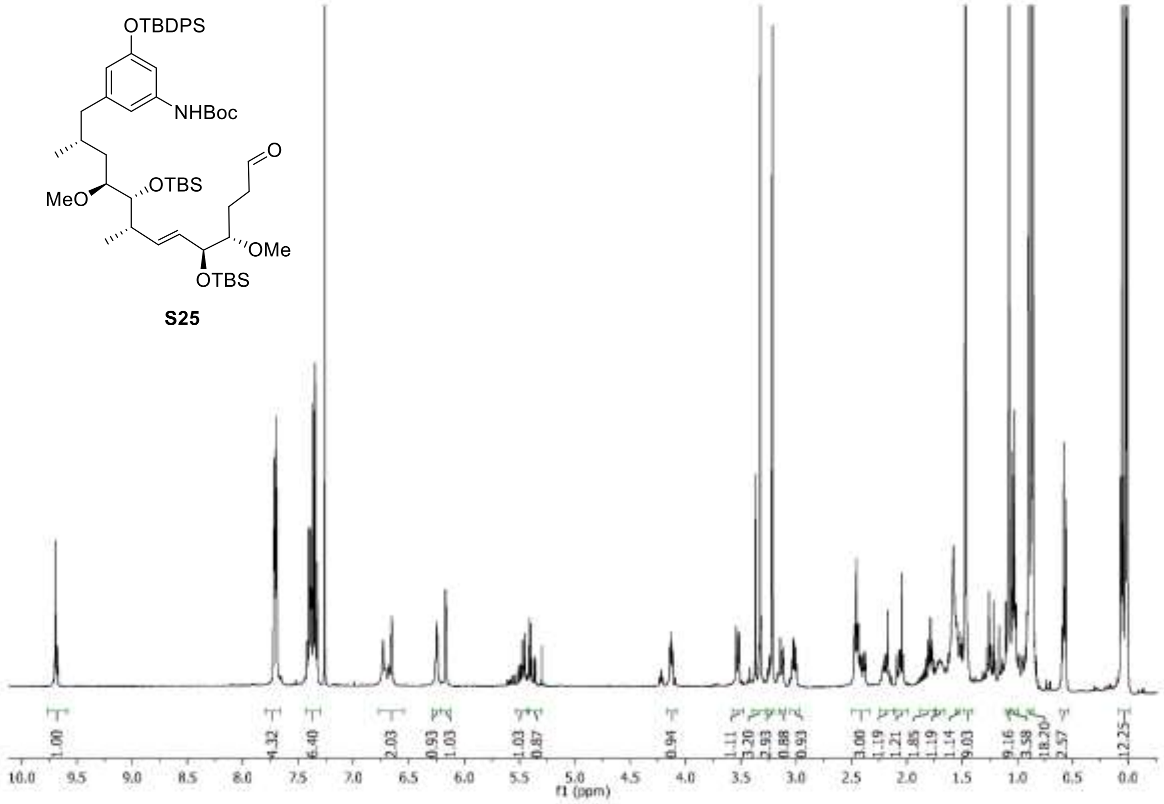

Figure S41: ^{13}C NMR spectrum of compound **S25**.

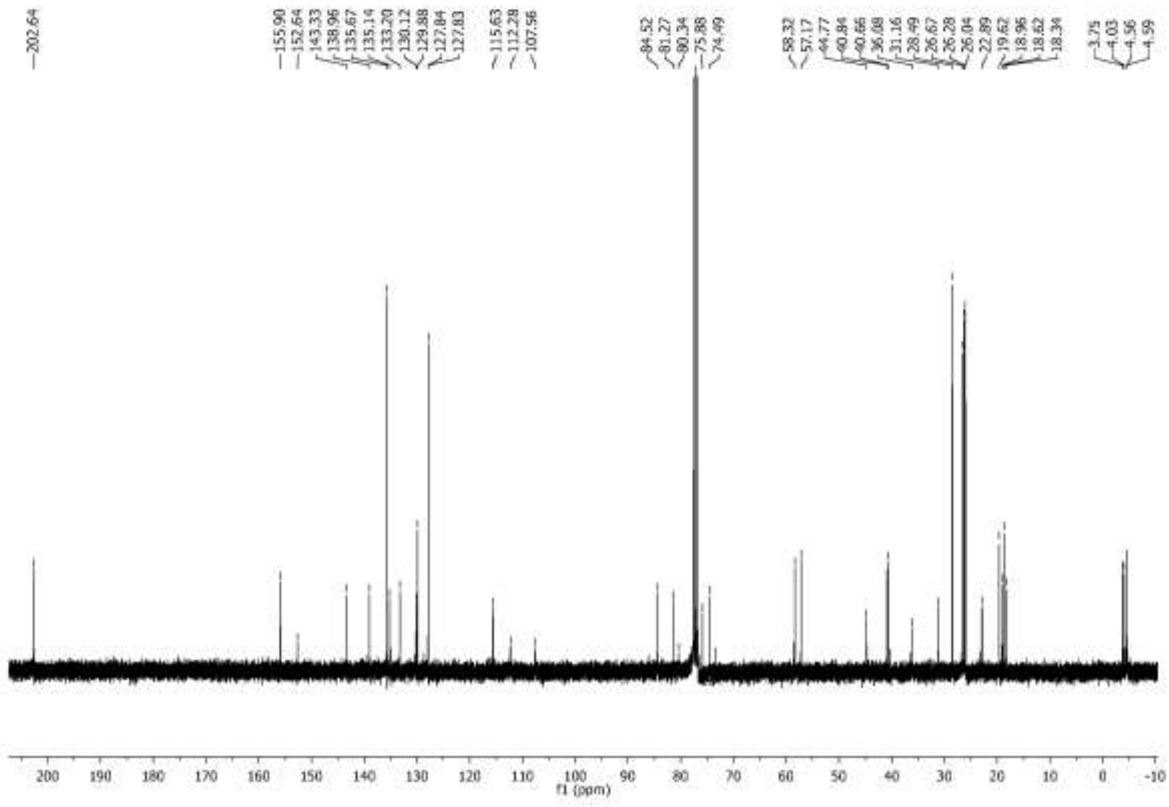

Figure S42: ^{1}H NMR spectrum of compound **29**.

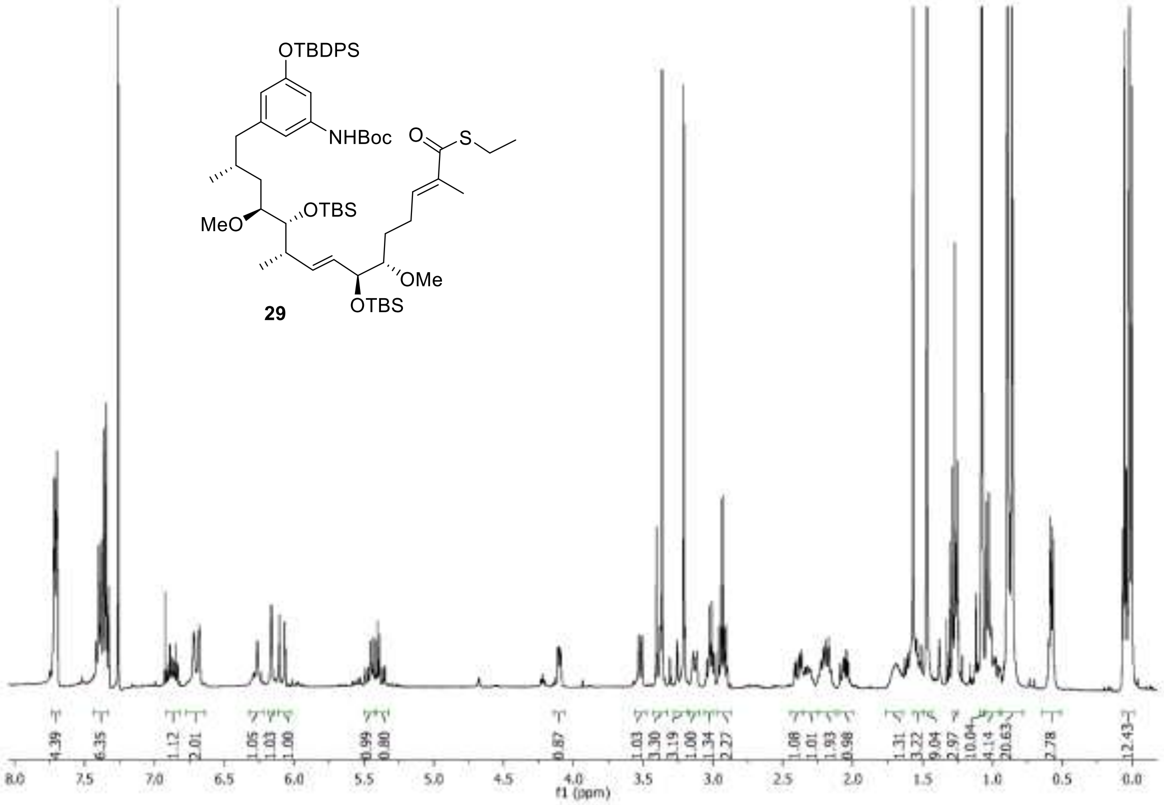

Figure S43: ^{13}C NMR spectrum of compound **29**.

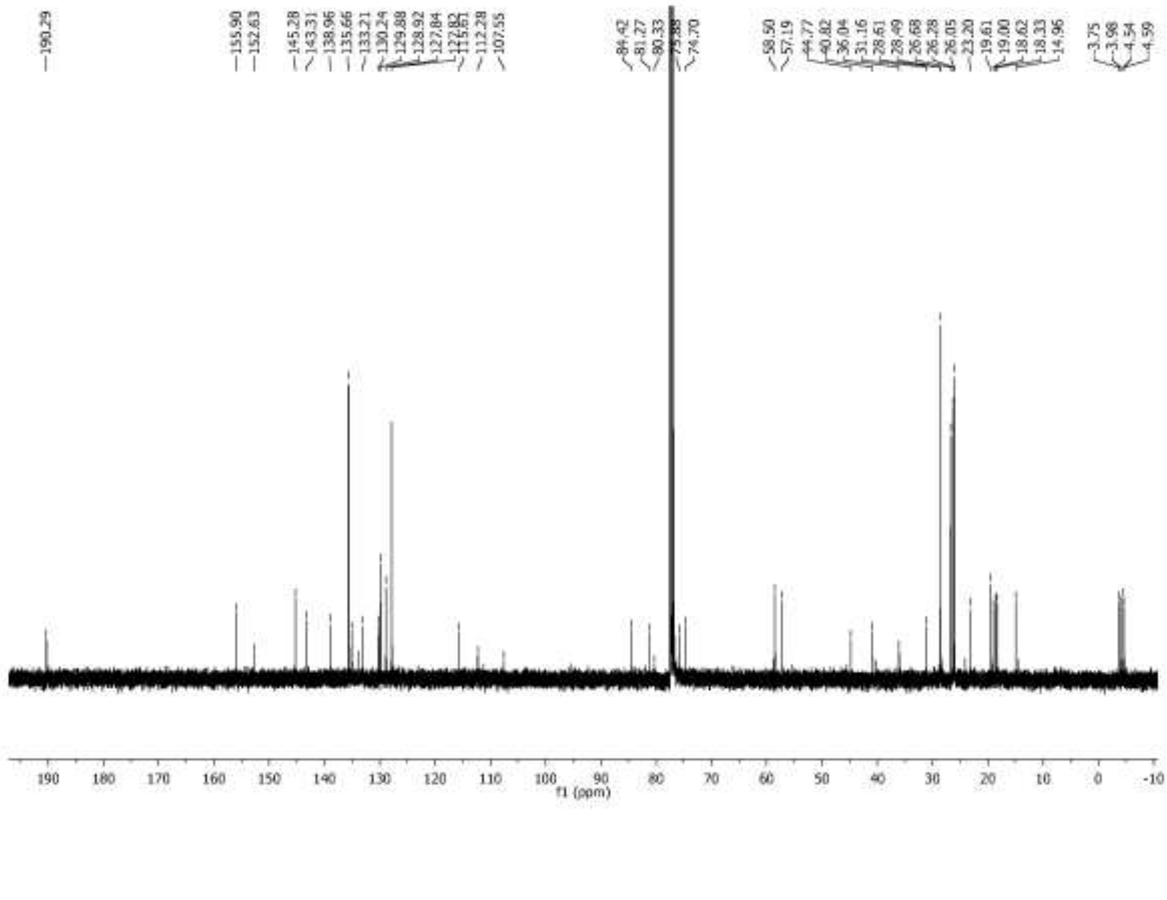

Figure S44: ^{1}H NMR spectrum of compound **30**.

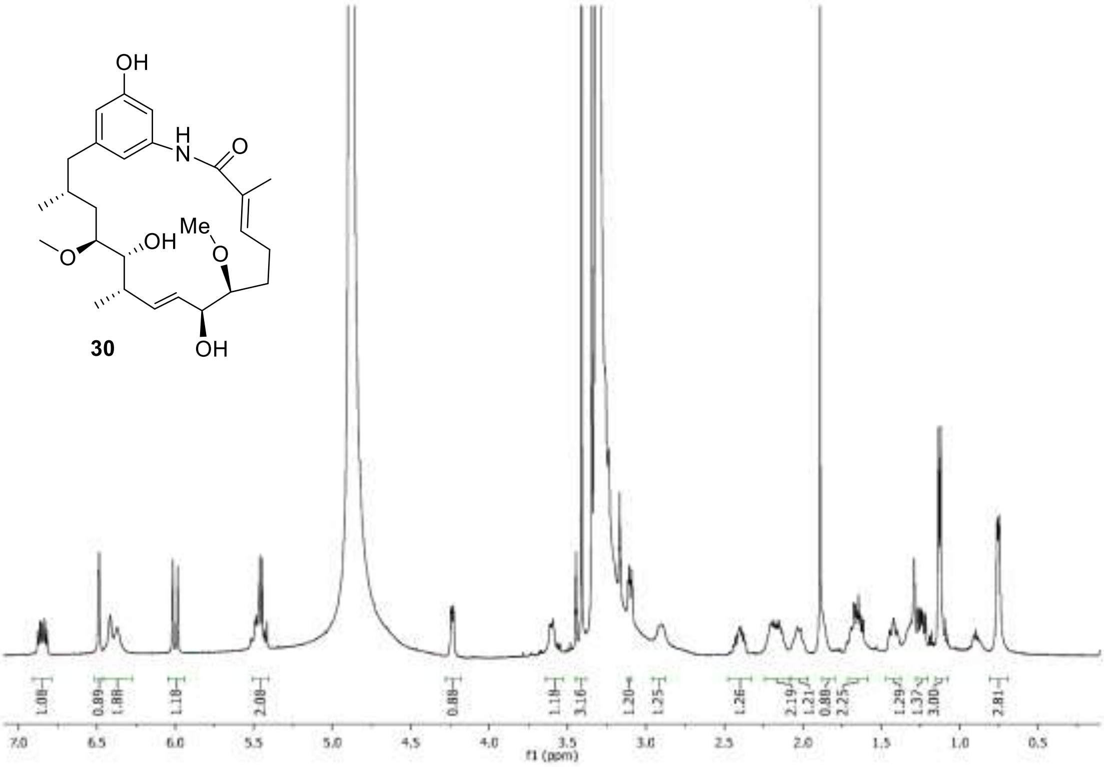

Figure S45: ^{13}C NMR spectrum of compound **30**.

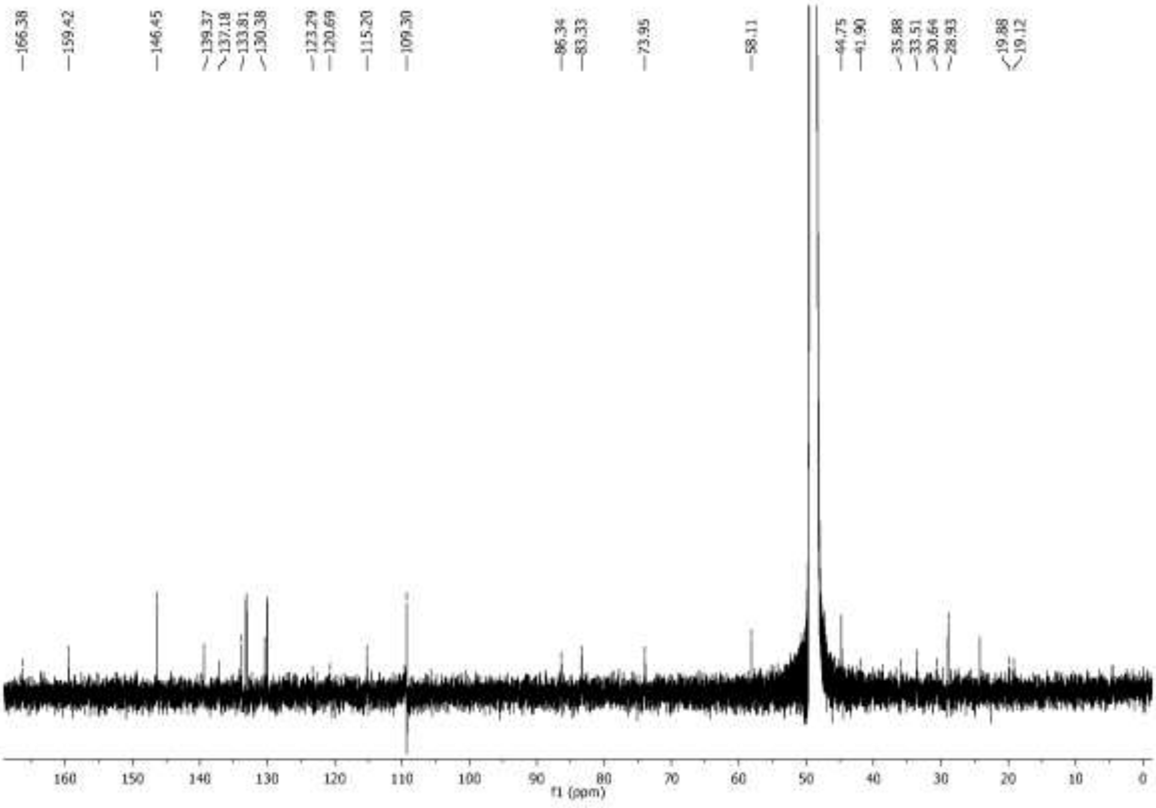

Figure S46: Uncropped scan of SDS-PAGE gel of the purified full-length *Sh*GdmF after gel filtration.

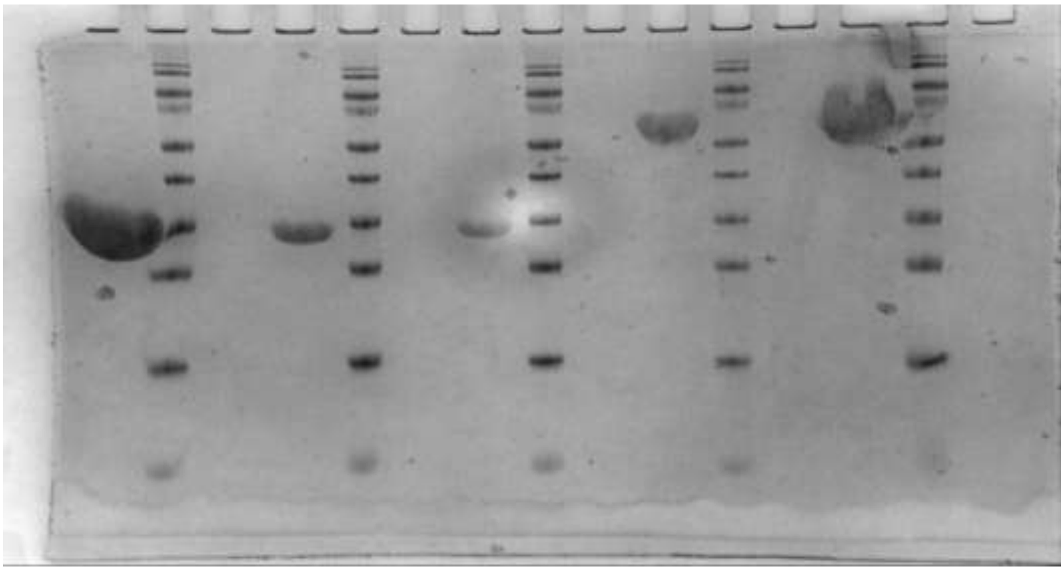

Supplement: Supplementary file 1 — Supplementary Information [file 41467_2025_57013_MOESM1_ESM.pdf]
