# Supplementary material for: Structure and function of the geldanamycin amide synthase from Streptomyces hygroscopicus
Source: Nat Commun. 2025 Mar 12;16:2464. doi: 10.1038/s41467-025-57013-3 (PMC11903869; doi:10.1038/s41467-025-57013-3)
Supplement: Supplementary file 2 — Reporting Summary [file 41467_2025_57013_MOESM2_ESM.pdf]

nature portfolio

Corresponding author(s): Matthias Preller, Carsten Zeilinger, Andreas Kirschning

Last updated by author(s): Nov 27, 2024

Reporting Summary

Nature Portfolio wishes to improve the reproducibility of the work that we publish. This form provides structure for consistency and transparency in reporting. For further information on Nature Portfolio policies, see our Editorial Policies and the Editorial Policy Checklist.

Statistics

For all statistical analyses, confirm that the following items are present in the figure legend, table legend, main text, or Methods section.

n/a	Confirmed	
☐	☒	The exact sample size (n) for each experimental group/condition, given as a discrete number and unit of measurement
☐	☒	A statement on whether measurements were taken from distinct samples or whether the same sample was measured repeatedly
☒	☐	The statistical test(s) used AND whether they are one- or two-sided<br>*Only common tests should be described solely by name; describe more complex techniques in the Methods section.*
☒	☐	A description of all covariates tested
☒	☐	A description of any assumptions or corrections, such as tests of normality and adjustment for multiple comparisons
☐	☒	A full description of the statistical parameters including central tendency (e.g. means) or other basic estimates (e.g. regression coefficient) AND variation (e.g. standard deviation) or associated estimates of uncertainty (e.g. confidence intervals)
☒	☐	For null hypothesis testing, the test statistic (e.g. F, t, r) with confidence intervals, effect sizes, degrees of freedom and P value noted<br>*Give P values as exact values whenever suitable.*
☒	☐	For Bayesian analysis, information on the choice of priors and Markov chain Monte Carlo settings
☒	☐	For hierarchical and complex designs, identification of the appropriate level for tests and full reporting of outcomes
☒	☐	Estimates of effect sizes (e.g. Cohen's d, Pearson's r), indicating how they were calculated

Our web collection on statistics for biologists contains articles on many of the points above.

Software and code

Policy information about availability of computer code

Data collection	X-ray data were collected at Synchrotron Radiation Facilities Soleil (France) and PetraIII (DESY, Germany); Multiskan FC Microplate Photometer (Thermo Fisher Scientific); GeSim Nano-PlotterTM (GeSim); GenePix-4000B Laser Scanner (Molecular Devices, Inc.); Bruker Ultrashield 500 MHz with Avance-II HD console; Ascend 400 MHz with Avance-III console; Ascend 400 MHz with Avance-III HD console; Ultrashield 400 MHz with Avance-I console; Ascend 600 MHz with Avance Neo console; Micromall LCT with lockspray source; PerkinElmer Polarimeter 341

Data analysis

XDS
Aimless (CCP4 suite)
Balbes (CCP4 web server)
Arp/wARP server
Phaser (Phenix)
Coot 0.8.9.2
phenix.refine 1.17.1_3660
Ima-Gene5 (BioDiscovery, Inc.)
NAMD2.13
CHARMM36 Force Field
Pymol 2.6.0a0 open-source
VMD 1.9.4
Origin2017G
NT analysis software (NanoTemper Technologies)

For manuscripts utilizing custom algorithms or software that are central to the research but not yet described in published literature, software must be made available to editors and reviewers. We strongly encourage code deposition in a community repository (e.g. GitHub). See the Nature Portfolio guidelines for submitting code & software for further information.

Data

Policy information about availability of data

All manuscripts must include a data availability statement. This statement should provide the following information, where applicable:

- Accession codes, unique identifiers, or web links for publicly available datasets
- A description of any restrictions on data availability
- For clinical datasets or third party data, please ensure that the statement adheres to our policy

Protein coordinates and structure factors are submitted to the Protein Data Bank (PDB) with accession codes: 8btm, 8oom, 8osv.
We added the following to our manuscript: "Data availability: The authors declare that data supporting the findings of this study are available within the paper (and its supplementary information files). Additional figures, chemical synthesis, analytical data and copies of NMR spectra as well as any associated references are available in the supporting information. These are available in the online version of the paper. The X-ray crystallographic data (final models and structure factor amplitudes) generated in this study have been deposited in the Protein Data Bank (www.rcsb.org) under accession codes 8btm, 8oom, and 8osv. Refinement statistics are listed in Table S1. Source data are provided with this paper. Reprints and permissions information is available online at http://www.nature.com/reprints/index.html. Correspondence and requests for matierials should be addressed to M.P., C.Z. or A.K."

Research involving human participants, their data, or biological material

Policy information about studies with human participants or human data. See also policy information about sex, gender (identity/presentation), and sexual orientation and race, ethnicity and racism.

Reporting on sex and gender	n/a
Reporting on race, ethnicity, or other socially relevant groupings	n/a
Population characteristics	n/a
Recruitment	n/a
Ethics oversight	n/a

Note that full information on the approval of the study protocol must also be provided in the manuscript.

Field-specific reporting

Please select the one below that is the best fit for your research. If you are not sure, read the appropriate sections before making your selection.

☒ Life sciences ☐ Behavioural & social sciences ☐ Ecological, evolutionary & environmental sciences

For a reference copy of the document with all sections, see nature.com/documents/nr-reporting-summary-flat.pdf

Life sciences study design

All studies must disclose on these points even when the disclosure is negative.

Sample size

Activity assays were performed in three independent measurements.
MST.based binding assays were performed in triplicates.
Microarray-based binding was measured from 10 individual spots.
MD simulations were done in triplicates.

Data exclusions	No data were excluded
Replication	Activity of GdmF was reproducibly confirmed from three measurements for the different substrates.<br>Binding was successfully reproduced in 10 spots.
Randomization	n/a
Blinding	n/a

Reporting for specific materials, systems and methods

We require information from authors about some types of materials, experimental systems and methods used in many studies. Here, indicate whether each material, system or method listed is relevant to your study. If you are not sure if a list item applies to your research, read the appropriate section before selecting a response.

Materials & experimental systems

n/a	Involved in the study	
☒	☐	Antibodies
☒	☐	Eukaryotic cell lines
☒	☐	Palaeontology and archaeology
☒	☐	Animals and other organisms
☒	☐	Clinical data
☒	☐	Dual use research of concern
☒	☐	Plants

Methods

n/a	Involved in the study	
☒	☐	ChIP-seq
☒	☐	Flow cytometry
☒	☐	MRI-based neuroimaging

Plants

Seed stocks	n/a
Novel plant genotypes	n/a
Authentication	n/a
